# Novel hydroxytyrosol esters as potential anti-amyloid and neuroprotective agents for Alzheimer's disease

**DOI:** 10.1039/d6md00265j

**Published:** 2026-06-04

**Authors:** Ioanna Kalpaktsi, Anthi Panara, Barbara Mavroidi, Giorgos Garcia Niforos, Amalia D. Kalampaliki, Ioanna C. Vlachogianni, Eleftheria A. Georgiou, Elizabeth Fragopoulou, Anthony Tsarbopoulos, Alexios-Leandros Skaltsounis, Maria Pelecanou, Kontantinos Palikaras, Evagelos Gikas, Ioannis K. Kostakis

**Affiliations:** a Division of Pharmaceutical Chemistry, Department of Pharmacy, National and Kapodistrian University of Athens Panepistimiopolis Zografou 15771 Athens Greece ikkostakis@pharm.uoa.gr; b Laboratory of Analytical Chemistry, Department of Chemistry, National and Kapodistrian University of Athens Panepistimiopolis, Zografou Athens 15771 Greece; c Institute of Biosciences & Applications, National Centre for Scientific Research “Demokritos” 15310 Athens Greece; d Department of Physiology, Medical School, National and Kapodistrian University of Athens Athens 11527 Greece; e Department of Nutrition & Dietetics, School of Health Sciences and Education, Harokopio University Athens Greece; f Department of Pharmacology, Medical School, National and Kapodistrian University of Athens 11527 Athens Greece; g Division of Pharmacognosy and Natural Products Chemistry, Department of Pharmacy, National and Kapodistrian University of Athens Panepistimiopolis Zografou 15771 Athens Greece

## Abstract

Alzheimer's disease (AD) is associated with the aggregation of β-amyloid (Aβ) peptides and oxidative stress, two interconnected processes that contribute to neuronal dysfunction and cognitive decline. Natural polyphenols such as oleuropein and its metabolite hydroxytyrosol display antioxidant and anti-amyloidogenic properties, but oleuropein suffers from limited stability due to glycosidic hydrolysis. To develop more robust and potent oleuropein analogs, we synthesized a series of hydroxytyrosol-based esters in which the secoiridoid glucoside scaffold of oleuropein was replaced by lipophilic substituents designed to enhance molecular stability and interactions with Aβ peptide. The compounds were evaluated for their ability to interact with Aβ40 using ESI-MS, circular dichroism (CD), and thioflavin-T fluorescence (ThT), along with complementary antioxidant assays. Most of the compounds formed stable non-covalent complexes with Aβ40, inhibited early aggregation events, and prevented the peptide's conformational transition from random coil to β-sheet. To assess biological efficacy and safety *in vivo*, the most promising analog (3b) was evaluated in *Caenorhabditis elegans* models of amyloid-β toxicity. Treatment with 3b exhibited no detectable toxicity in wild-type animals, as evidenced by normal development, growth, and reproductive efficacy. Importantly, 3b rescued lifespan shortening and locomotor deficits in transgenic nematodes expressing human Aβ42 pan-neuronally, while having no effect on control strains lacking Aβ42 expression. These findings demonstrate that 3b confers functional protection against amyloid-induced toxicity *in vivo*. Overall, our results identify the newly synthesized hydroxytyrosol-derived esters as promising multifunctional scaffolds that combine potent anti-aggregation activity with strong antioxidant properties and *in vivo* neuroprotective efficacy, supporting their further development as anti-amyloidogenic agents for AD therapy.

## Abstract 2

Hydroxytyrosol-derived esters emerge as stable oleuropein mimetics that inhibit Aβ aggregation and confer *in vivo* neuroprotection.

## Graphical abstract

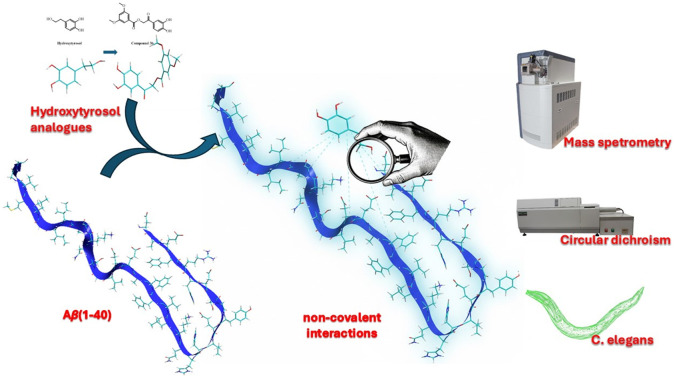

## 1. Introduction

The demographic shift towards an increasingly older society has resulted in a rising prevalence of chronic age-related diseases across industrialized countries. The development of degenerative diseases, such as Alzheimer's disease (AD), which is associated with neurodegeneration, impaired synaptic function, and massive brain cell loss, decline of cognitive ability and premature death, has significant health and economic implications in Western societies.^1–3^ While the exact cause of Alzheimer's disease remains not fully understood, it is believed to arise from a complex interplay of genetic, environmental, and lifestyle factors. The pathophysiology of AD is characterized by the accumulation of beta-amyloid plaques and neurofibrillary tangles in the brain. These pathological formations disrupt communication between brain cells, leading to neuronal loss and the progressive decline of cognitive ability.^4–6^ Although the aggregation of beta-amyloid and tau proteins is the main characteristic feature of the disease, other contributing factors, such as neuroinflammation or genetic predisposition, may act in concert with these protein abnormalities to promote the progression of AD.^7–9^

The beta-amyloid peptide (Aβ) is the major proteinaceous component of senile plaques that characterize Alzheimer's disease (AD).^8,10–12^ The main mechanisms proposed to justify Aβ's neurotoxicity involve oxidative stress and dysregulated inflammatory/immune responses, which are considered to play pivotal roles in AD pathogenesis.^13^ Growing evidence suggests that reducing beta-amyloid (Aβ) accumulation in the brain could positively impact the progression of AD.^9^ Various strategies are being explored to achieve this goal, including the modulation of Aβ production, enhancement of Aβ clearance, inhibition of Aβ aggregation, promotion of Aβ degradation, and prevention of Aβ-induced neuronal damage.^14–16^ Given the association between free radicals and neurodegeneration, leading to cell death, strategies aiming to reduce reactive oxygen species (ROS) generation may be beneficial in AD treatment.^17–19^

**Fig. 1:**
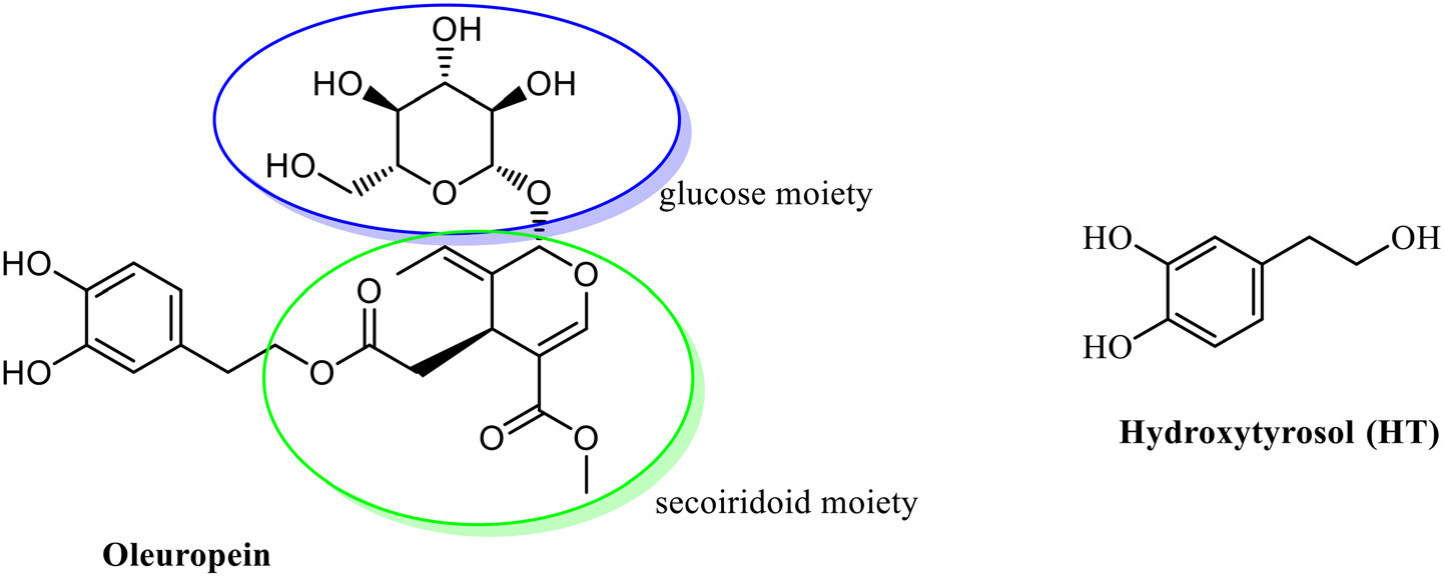
Structure of oleuropein (OE) and hydroxytyrosol (HT).

Natural polyphenols have attracted considerable interest as multifunctional agents capable of modulating oxidative stress, metal homeostasis, inflammation, and amyloid aggregation.^20–23^ Oleuropein (OE), a major phenolic secoiridoid in olive leaves and pulp, and its metabolite hydroxytyrosol (HT) exhibit potent antioxidant and radical-scavenging activity (Fig. 1).^24–28^ Prior work from our group demonstrated that OE forms strong non-covalent complexes with Aβ, thereby modulating early aggregation events.^29,30^ However, the therapeutic potential of OE is limited by its instability, primarily due to glycosidic hydrolysis of the secoiridoid glucoside scaffold.^31^

To overcome these limitations, we designed and synthesized a series of hydroxytyrosol-based analogs in which the secoiridoid glucoside scaffold of OE was replaced with simple lipophilic substituents. This strategy aims to (i) enhance molecular stability, (ii) preserve or improve antioxidant properties, and (iii) strengthen interactions with Aβ. The present study evaluates these new derivatives through a comprehensive panel of biophysical and biological assays to identify scaffolds with improved anti-amyloidogenic potential.

## 2. Results and discussion

### 2.1 Design

Building on previous observation of our group that oleuropein (OE) forms stable non-covalent complexes with Aβ, we sought to develop hydroxytyrosol (HT) derivatives that retain the beneficial catechol scaffold while addressing the chemical and enzymatic instability of OE. The instability of OE arises primarily from hydrolysis of its glycosidic bond, which limits its therapeutic potential. To address this limitation, the glycosidic moiety was replaced with simple lipophilic ester substituents, yielding HT esters that are expected to be more resistant to hydrolysis while preserving the core antioxidant properties of hydroxytyrosol. Although hydroxytyrosol esterification has previously been explored to enhance lipophilicity and biological activity,^32–34^ the present derivatives introduce key distinctions – replacement of the oleuropein glycosidic scaffold with lipophilic esters and strategic α-carbon modifications designed to probe structure–activity relationships and optimize Aβ interactions.

These features collectively extend beyond prior studies and substantiate the potential improved performance of the present derivatives. In addition to improving stability, specific structural modifications were introduced to probe structure–activity relationships and enhance Aβ interactions. Lipophilic ester substituents were designed to increase hydrophobicity, hypothesized to strengthen interactions with hydrophobic patches of Aβ aggregates, potentially inhibiting β-sheet formation and aggregation. Substitutions at the α-carbon of the catechol side chain were included to modulate steric and hydrogen-bonding properties, allowing exploration of how small structural variations influence Aβ binding and anti-aggregation activity. Retention of the catechol core ensures that antioxidant and radical-scavenging properties are preserved, which may mitigate oxidative stress associated with Aβ toxicity. This design strategy integrates stability, physicochemical optimization, and potential Aβ-binding interactions. The resulting derivatives were evaluated *in vitro* for their ability to modulate Aβ aggregation using electrospray ionization mass spectrometry (ESI-MS), circular dichroism (CD), and thioflavin T (ThT) assays. Finally, the *in vivo* efficacy against amyloid-β toxicity was evaluated using a *Caenorhabditis elegans* model. The goal of this study was to identify lead compounds with enhanced anti-amyloidogenic activity suitable for further optimization.

### 2.2 Synthesis

**Scheme 1:**
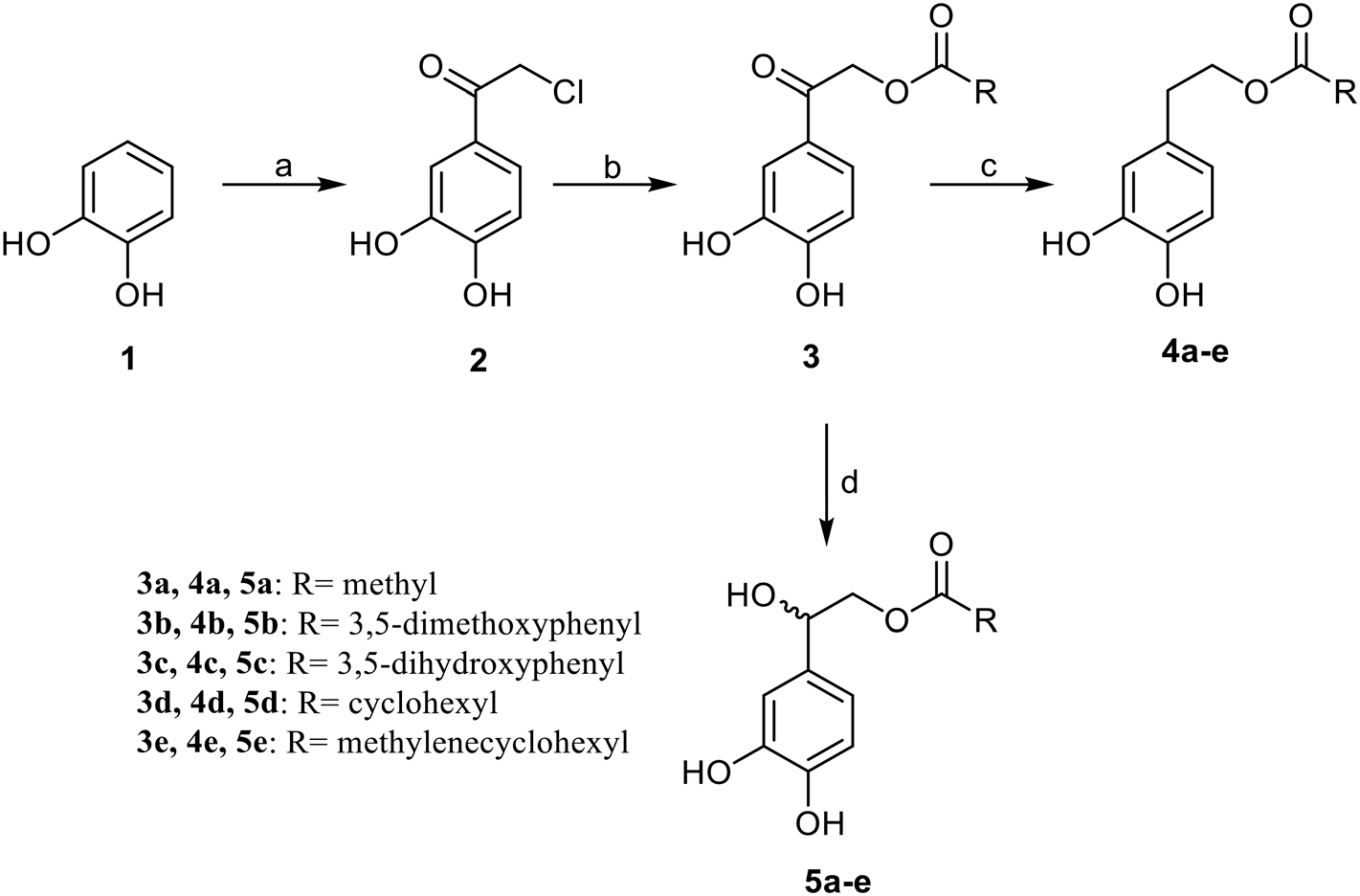
Reagents and conditions: a) chloroacetylchloride, POCl_3_, 110 °C, 12 h, 92%; b) appropriate acid, NaH, 34–87%; c) triethylsilane, CF_3_CO_2_H, 70 °C, 4 h, 65–95%; d) H_2_, Pd/C, 50 psi, rt, 4 h, 74–92%.

Catechol (1) was used as starting material and converted to the corresponding chloroacetonide 2 by reaction with chloroacetyl chloride in POCl_3_ (Scheme 1). Nucleophilic substitution with sodium salts of the appropriate acids yielded keto analogs 3a–f in yields up to 80%, with excellent reproducibility and scalability to gram quantities. Treatment of the later compounds with triethylsilane in trifluoroacetic acid, provided the desired, fully reduced, lipophilic esters 4a–e, while partial reduction with hydrogenation over Pd/C, provided the hydroxy derivatives 5a–e, as racemic mixtures.

### 2.3 Biophysical studies

Aβ aggregation and oxidative stress are intimately linked in AD progression. Small molecules capable of both inhibiting Aβ self-assembly and mitigating oxidative insults represent promising multifunctional therapeutic candidates. Accordingly, the synthesized derivatives were evaluated for their effect on Aβ40 aggregation using mass spectrometry, circular dichroism, and thioflavin-T fluorescence.

#### 2.3.1 Mass spectrometry studies of Aβ40 aggregation inhibition

To investigate the ability of the newly synthesized HT analogs to form non-covalent complexes with Aβ, ESI Q-ToF high-resolution mass spectrometry (HRMS) was employed. The ESI source parameters were optimized to preserve non-covalent interactions, with the capillary voltage maintained at 3.5 kV and the desolvation temperature set at 120 °C. The presence of peptide–ligand complexes was verified by monitoring mass shifts corresponding to the sum of the molecular weight of Aβ40 and each of the tested analogs. As Aβ can adopt multiple charge states, all the ionic species of the multicharged complex were examined. Under these ionization conditions, the +5 charge state of Aβ predominated and was therefore selected for the study of peptide–HT analog interactions. The results were expressed as the percentage intensity of the +5 charge state of Aβ40. This approach allows direct comparison of complexed and non-complexed Aβ without altering the charge-state distribution of the peptide. To benchmark interaction strength and ensure reproducibility of the experimental conditions, the monitored interactions were bracketed by infusion of an Aβ–oleuropein complex, which served as a positive control (Fig. S1).

In addition to 1 : 1 complexes, several HT derivatives were observed to bind two ligand molecules per Aβ peptide, forming Aβ–2X complexes (where X is HT derivative). As such multivalent interactions may contribute to inhibition of Aβ aggregation, the overall complexation capacity (intensity_complex_), was calculated as the intensity sum of the Aβ–X and Aβ–2X species for the +5 charge state, expressed as intensity_complex_ = [∑(Aβ–X + Aβ–2X)]. The specificity and stability of the peptide–ligand complexes were further evaluated under varying experimental conditions, including increased capillary voltage, acidic pH, and increasing Aβ concentrations ranging from 5 to 100 μM. Notably, ESI signals corresponding to the non-covalent complex between Aβ and ester 3b were consistently observed across all concentration levels, indicating the high robustness of this interaction under the specific conditions.

**Fig. 2:**
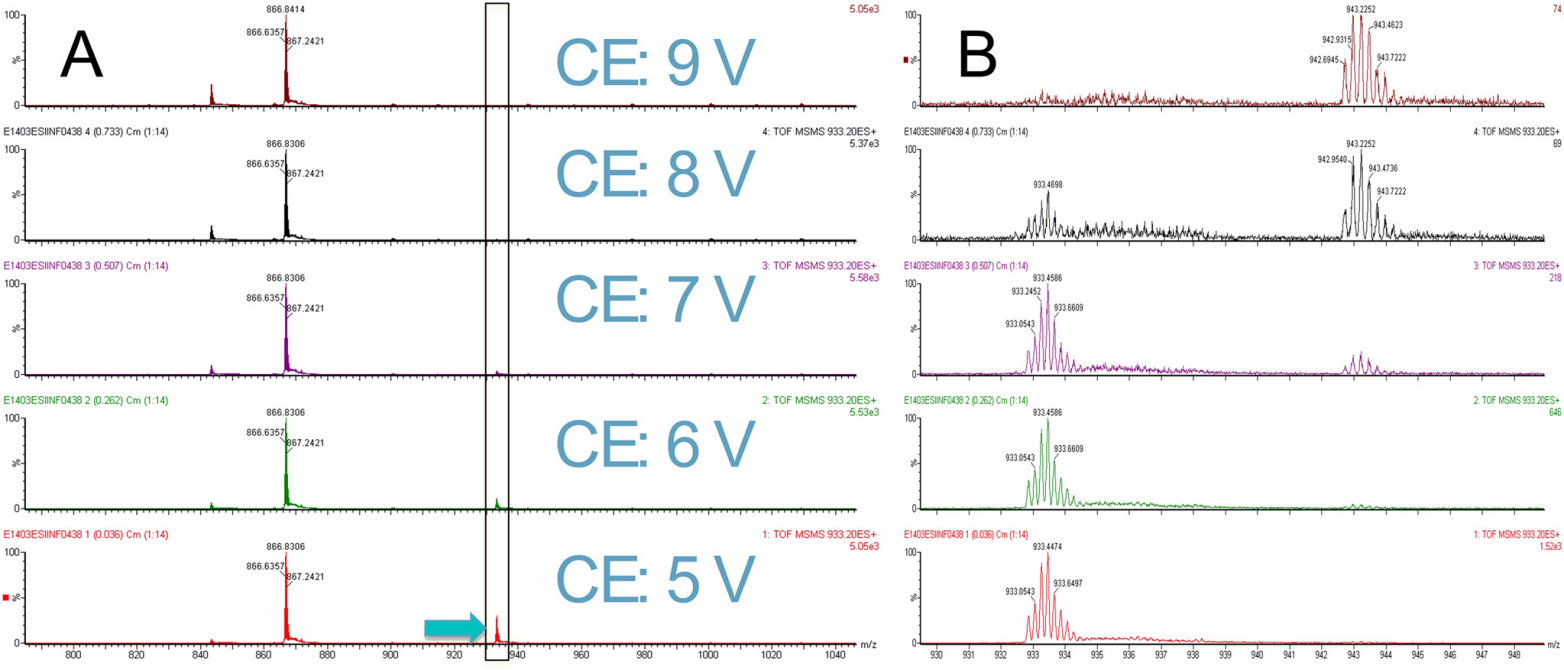
Positive ion ESI MS/MS of the complex of Aβ with one molecule of the compound 3b (1 : 1 complex) at charge state +5. Panel A shows the fragment envelope (with higher ion at 866.8) and the green arrow indicates the molecular ion. Panel B shows the molecular ion indicated by the green arrow. The results show that the complex fragments almost completely at a collision energy of 9 V (CE = collision energy).

Complex stability was further assessed by MS/MS experiments. The Aβ–HT complex at the +5 charge state, which exhibited the most intense signal, was selected as the precursor ion and subjected to collision-induced dissociation (CID). Fragmentation experiments revealed comparable dissociation behavior for all complexes, with a 50% reduction in signal intensity observed at approximately 12.5 V and breakdown energies ranging from 4.8 to 5.7 V. These results suggest that, once formed, the stability of the complexes is similar across the series (Fig. 2).

Analysis of the complexation data revealed that among the tested compounds, derivatives 3b and 3c exhibited the highest complexation efficiency with Aβ. These analogs are characterized by the presence of a carbonyl group at the α-position of the aliphatic side chain of HT and an aromatic ester substituent, highlighting the importance of both features for effective interaction. In contrast, derivatives bearing hydroxyl substitution at the same position (compounds 5a, c–e) displayed moderate complexation ability, while the corresponding HT analogs (compounds 4a, c–e) lacking additional substitution showed the weakest interaction with Aβ. Overall, the data suggest that incorporation of a carbonyl group enhances significantly the peptide–ligand complexation, presumably through additional hydrogen bonding or dipolar interactions. Moreover, ester substituents containing aromatic rings led consistently to stronger complexation (as observed for compounds 3b, 4b and 5b) compared with aliphatic analogs, indicating that π–π stacking and/or hydrophobic interactions play a crucial role in stabilizing the complexes. Nevertheless, the presence of polar hydroxy substituents to the aromatic ring appears to reduce complexation efficiency (Table 1).

**Table 1:** Relative complexation efficiency of hydroxytyrosol (HT) derivatives with Aβ, as determined by ESI-HRMS. Values represent the summed relative intensities of the Aβ–X complexes for the +5 charge state

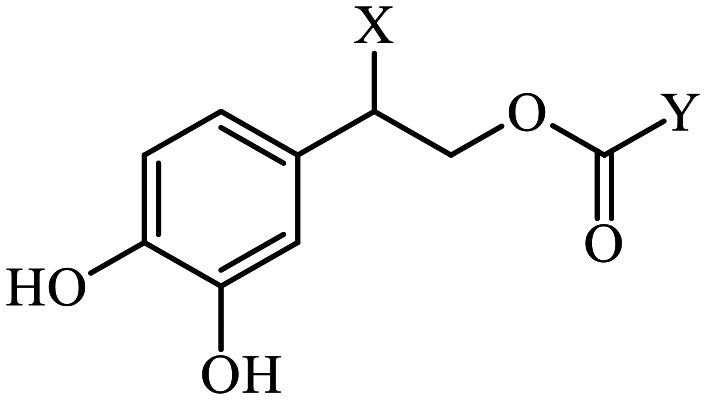		Y				
		[Aβ–X] + 5				
		CH_3_	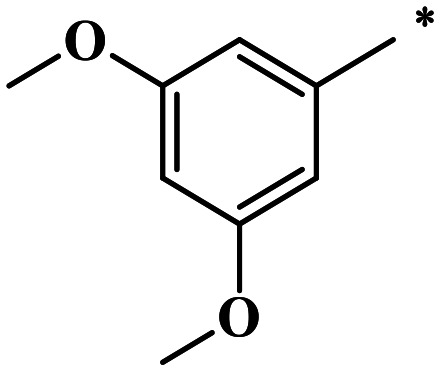	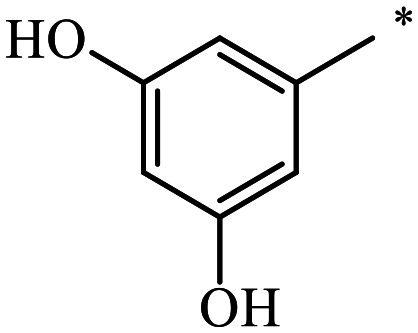	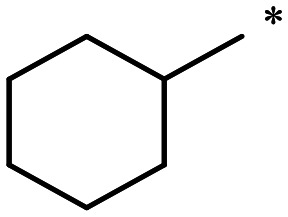	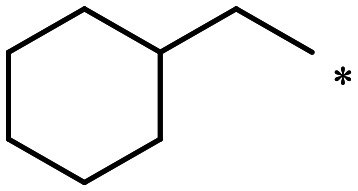
X	C=O	3a	3b	3c	3d	3e
		—	50.4%	29.5%	7.4%	7.4%
	H	4a	4b	4c	4d	4e
		—	18.4%	4.3%	0.6%	—
	OH	5a	5b	5c	5d	5e
		1.4%	25.8%	8.4%	—	0.5%

#### 2.3.2 QSAR modeling for Aβ complexation

To gain a deeper insight into the properties that govern the activity of the HT analogs, a QSAR model was developed correlating the complexation potency with a set of molecular descriptors. 3D molecular descriptors were calculated after geometry optimization of all HT analogs at the semi-empirical level using MOPAC 22.10 and the PM7 Hamiltonian.^35^ For the purposes of the current methodology, which relies on the topological representation, the PM7 Hamiltonian offers sufficient structural accuracy (typically within a fraction of an Å compared with higher level accuracy). On the other hand, geometry optimization of molecular structures at higher accuracy level utilizing the DFT level of theory, is significantly high computationally cost and it is essential of studies of accurate electronic structure representations.^36^ The descriptors employed were derived from the Dragon 5.5 suite as implemented in OCHEM, selected for their established interpretability and adequate coverage of the explored chemical space.^37^ Multivariate statistical analysis was performed using SIMCA 14.1 (Umetrics, Umeå, Sweden) employing partial least squares regression (PLS-R). All variables were normalized using Pareto scaling. Due to the limited number of available compounds, a statistically meaningful division into independent training and test sets was not feasible. Accordingly, all analogs were included in a single modeling set and internally validated using cross-validation procedures.

Analogs exhibiting zero or near-zero complexation capability were excluded from the primary model to avoid introducing variance that does not contribute to the dependent variable. Specifically, five compounds with different side-chains exhibited no detectable complexation ability; their inclusion would lead to pronounced aliasing effects due to a lack of discriminatory power. To validate this approach, three models were interrogated. UV scaling has been employed to all models investigated. The initial inclusive model (model 1), containing all descriptors and samples, yielded an *R*_2_ of 0.69 and a *Q*_2_ of 0.51. Performance improved significantly when focusing on samples exhibiting complexity (model 2: *R*_2_ = 0.99, *Q*_2_ = 0.85). The final refined model (model 3) utilized selected descriptors and samples with complexing activity (*R*_2_ = 0.94, *Q*_2_ = 0.998). The statistical power of model 1 suggests that a valid underlying QSAR structure exists even in the presence of noise. Subsequently the refinement of models 2 and 3, by excluding non-informative compound and variables, provide excellent QSAR models. The descriptors identified as most significant in model 3 were consistently present in the broader model 1, confirming that the structure–activity trends remain stable while effectively isolating the signal from the structural noise of inactive analogs. Five principal components (PCs) found to explain the largest part of variability without loss of the predicted capacity as determined by a scree plot. Although these high predictive metrics may suggest a risk of overfitting, the robustness of the reduced model is supported by permutation testing and CV-ANOVA analysis, and cross validation testing (CV). The correlation between experimental and calculated complexation activity values, derived from the reduced QSAR model, is shown in Fig. S2 of the SI. Permutation testing (*n* = 100, 250, 500 random permutations Fig. S3–S5) confirmed that the model was not the result of randomness, while the CV-ANOVA *p*-value (0.025) indicated statistical significance. CV testing was performed with number of groups (*k* = 2, 5, 7, 10, 16) and the corresponding metrics are tabulated on Table S2 of the SI. No outliers were identified based on the DModX (Fig. S6) and Hotelling's *T*^2^ tests (Fig. S7).

From the initial set of 434 descriptors, 11 were identified with coefficient values above 1.0, while all others laid below 0.5. The descriptors found to be important include SCBO, EEig03x, EEig06x, EEig13x, EEig02d, EEig03d, EEig04d, EEig09d, EEig11d, AEigv and AEigp. Those were selected as the primary drivers of complexation activity to form a parsimonious reduced model. Among these, eight belong to the edge-adjacency eigenvalue (EEig) descriptor family, two correspond to adjacency matrices weighted by atomic properties (AEig), and SCBO represents a bond-order descriptor. The EEig03x, EEig06x, and EEig13x descriptors encode the distribution of electronegativity across the molecular bond network, thereby reflecting charge distribution, polarization effects, and potential hydrogen-bonding or receptor interactions. The EEig(x)d family of descriptors correspond to different eigenvalue orders of polarizability and are associated with dispersion interactions, π-stacking potential, or non-covalent binding patterns. The AEigv represents the overall valence distribution within the molecular graph and is closely related to branching and substitution, whereas AEigp depicts the induced polarization to the molecular graph through the imposed interactions. Among these descriptors, AEigp exhibits the largest coefficient and is positively correlated with molecular polarity, reflecting induced charge distribution across the structure. This is followed by AEigv, which is associated with molecular shape and branching, and SCBO, which correlates with bond order and thus captures the contribution of aromatic character within the side chain. The relative contribution of each descriptor across the five principal components was evaluated using w*c weights and the results are tabulated on Table 2, with the component of the highest contribution for each descriptor is highlighted in bold. The first two components account for the primary variance for 8 out of the 11 descriptors, only 3 descriptors are explained by higher components (PCs 3–5). Although the EEig descriptor family contributes less prominently to the first principal component, exclusion of these descriptors results in a model with substantially reduced fitting and descriptive capability. Overall, a quantitative relationship between molecular structure and Aβ-complexation efficiency of the HT analogs has been established, providing a helpful basis for considering the design of future compounds with targeted binding properties. A limitation of the current that considers only a limited number of compounds showing activity through one mode of action (*i.e.* the complexing of Aβ).

**Table 2:** Statistical weights for the investigated descriptors along the model's principal components

Descriptor	M6.w*c [1]	M6.w*c [2]	M6.w*c [3]	M6.w*c [4]	M6.w*c [5]
SCBO	0.25	**0.79**	0.29	0.019	0.28
EEig03x	0.040	0.28	**0.43**	0.31	−0.018
EEig06x	0.041	**0.13**	−0.23	−0.45	−0.16
EEig13x	0.058	**0.11**	−0.37	−0.36	0.11
EEig02d	0.031	0.21	0.33	**0.83**	0.77
EEig03d	0.035	**0.053**	−0.46	−0.26	0.031
EEig04d	0.063	**0.32**	0.090	−0.066	−0.55
EEig09d	0.055	0.20	−0.27	**0.38**	−0.42
EEig11d	0.071	**0.11**	−0.29	0.0083	0.088
AEigv	**0.64**	−0.27	−0.30	0.069	0.12
AEigp	**0.71**	0.020	0.30	−0.049	−0.15

#### 2.3.3 Circular dichroism studies of Aβ40 aggregation inhibition

Circular dichroism spectropolarimetry (CD) provides a sensitive tool to monitor the aggregation process of Aβ *in vitro*. The structural changes that Aβ40 undergoes as it transitions from random coil monomers, to β-sheet assemblies, progressively leading to formation of amyloid fibrils, are reflected in the CD spectra over time.^38^ CD is commonly employed in the screening for Aβ aggregation inhibitors as alterations in the typical aggregation profile provide evidence on interaction with Aβ and interference with the fibrillization process.^39^

**Fig. 3:**
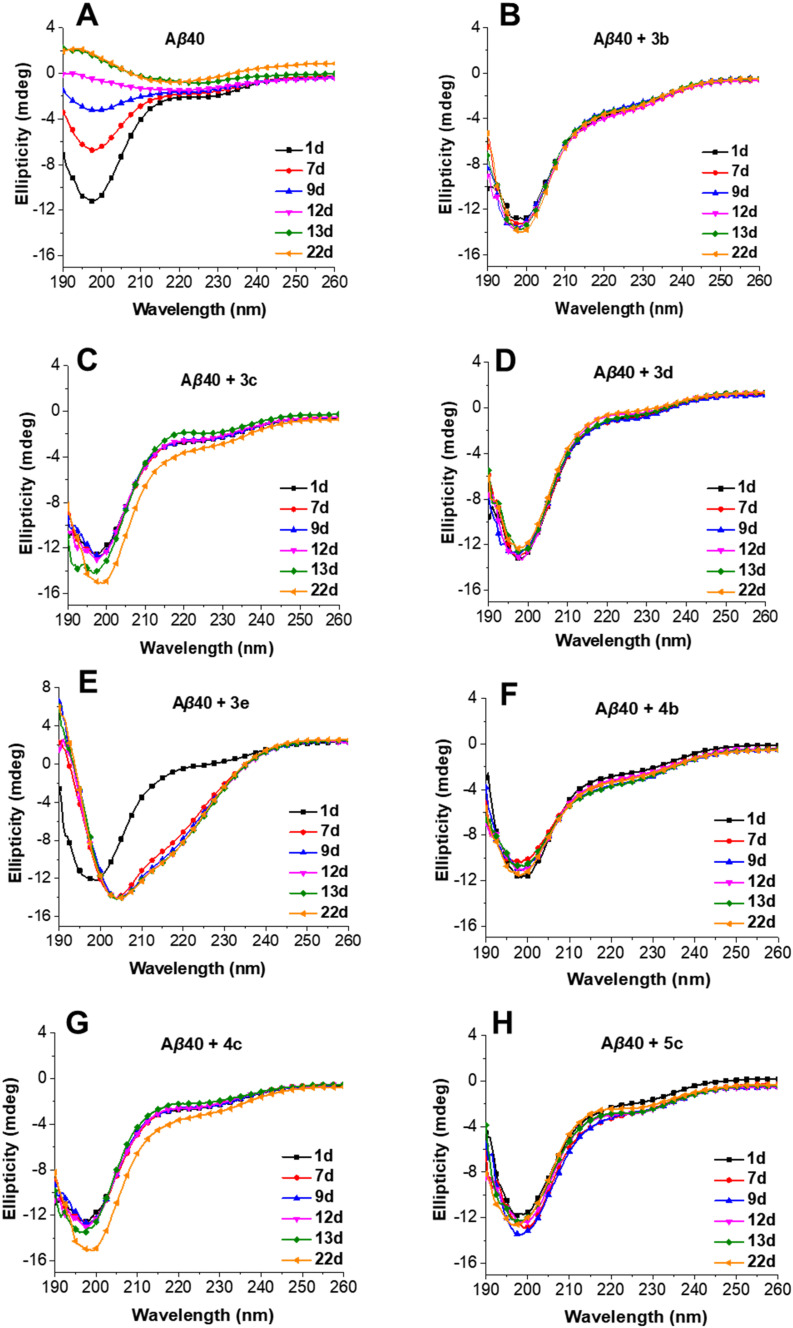
CD spectra of Aβ40 in the absence (A) and in the presence of 3b, 3c, 3d, 3e, 4c, 4d and 5c. (B–H) in phosphate buffer, pH 7.4. Spectra were collected for a period of 22 days at 33 °C. Representative spectra from *n* = 3 independent experiments are shown.

A representative CD profile associated with Aβ40 aggregation (phosphate buffer, pH 7.4, 33 °C) is shown in Fig. 3A. The initial (day 1) random coil spectrum of Aβ40 (negative band near 200 nm) evolves as the peptide progressively associates into β-sheet-rich oligomeric and higher-order structures co-existing in solution, ultimately yielding (day 22) the characteristic β-sheet spectrum of amyloid fibrillar forms (negative minimum near 217 nm, positive maximum around 195 nm). The reduced intensity observed at later stages of aggregation is attributed to precipitation of mature fibrils from solution.

The impact of the 3b, 3c, 3d, 3e, 4b, 4c, and 5c derivatives, which display complexation efficiency in the ESI-MS study (section 2.3.1) on the CD aggregation profile of Aβ40 was evaluated to gain insight on their potential anti-aggregation activity. As can be seen in Fig. 3(B–H) all compounds had a profound impact on the aggregation profile of Aβ40 (displayed in Fig. 3A) under the same conditions. More specifically, compounds 3b, 3c, 3d, 4b, 4c, and 5c effectively inhibited the transition toward β-sheet-rich fibrillar structures, maintaining the peptide in a predominantly random-coil state for 22 days. In the case of 3e, the CD profile (Fig. 3E) indicates the coexistence of random coil and extended β-sheet structures that remain soluble and do not precipitate from solution; in other words, they do not proceed to formation of typical amyloid fibrils. The pronounced red shift of the random coil negative band to 204 nm further supports the presence of strong interactions with the peptide. In addition, the deepening of the negative random coil band observed in some derivatives (*e.g.*, 3c and 4c; Fig. 3C and G), accompanied by a red shift of about 2 nm provides additional evidence of interaction with Aβ40 that affects its three-dimensional structure.

Even though differences are noted in the CD profiles obtained in the presence of the various derivatives, the general conclusion is that all compounds studied have an impressive effect on the Aβ40 aggregation pathway. Neither the structure of the R substituent nor the structure of the alpha-carbon of the hydroxytyrosol side chain appear to influence the inhibition of amyloid fibril formation. Overall, the CD results strongly indicate that the newly generated scaffold possesses a geometry that enables effective interaction with Aβ40. These findings are in full agreement with the MS-ESI complexation data (section 2.3.1) which show that the complexes, once formed, display similar stability across the series.

#### 2.3.4 Thioflavin T assay

**Fig. 4:**
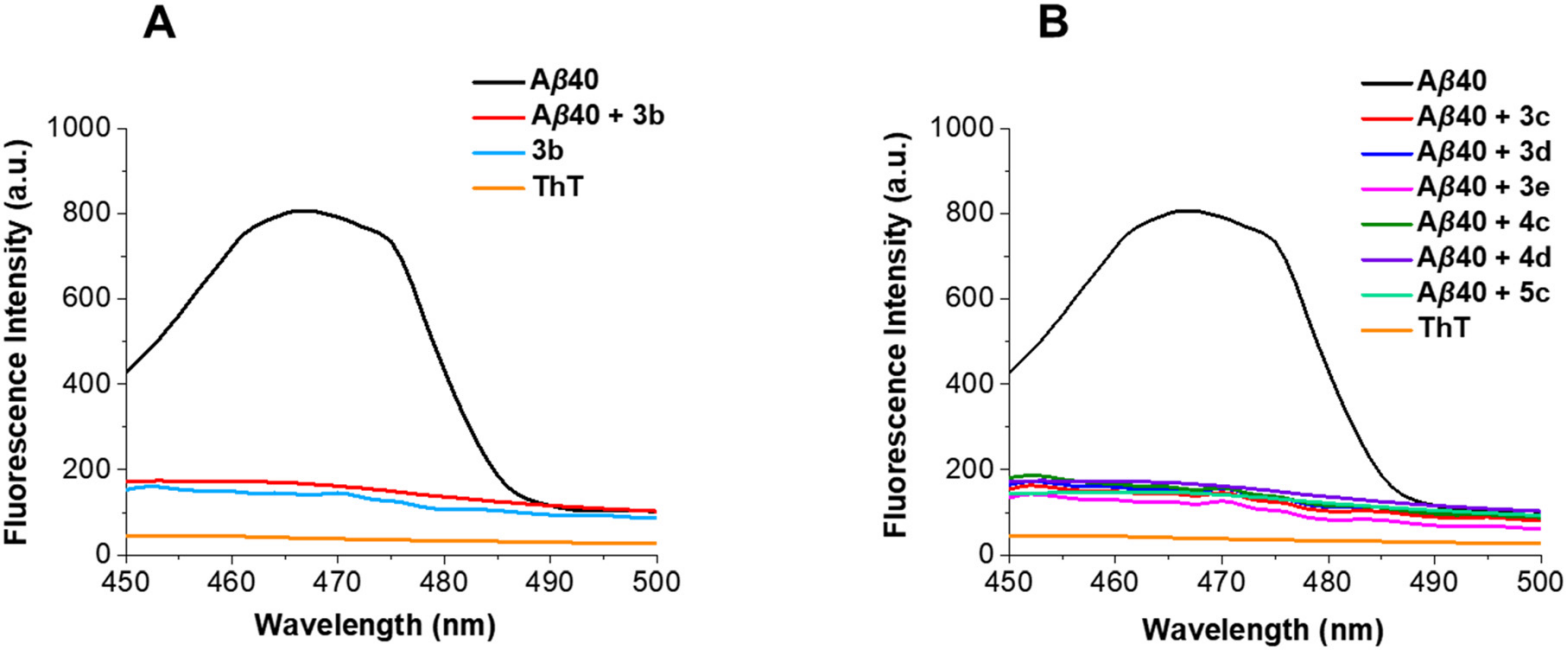
(A) ThT spectra of Aβ40 in the absence and presence of 3b. For comparison purposes, the fluorescence spectra of 3b alone and of ThT alone are included in the graph. (B) ThT spectra of Aβ40 in the absence and presence of 3b and 3c, 3d, 3e, 4c, 4d and 5c. Fluorescence was monitored following excitation at 440 nm. Representative spectra from *n* = 3 independent experiments are displayed.

To validate the inhibitory action on Aβ40 aggregation observed by CD, the incubation mixtures of Aβ with derivatives 3b, 3c, 3d, 3e, 4b, 4c, and 5c were analysed using the thioflavin-T (ThT) fluorescence assay, a widely used method for selectively detecting and quantifying amyloid fibrils.^40^Fig. 4A shows representative results from the ThT assay for 3b. The control Aβ40 sample produced a strong fluorescence signal characteristic of ThT binding to amyloid fibrils. In marked contrast, the Aβ40 sample incubated with 3b displayed dramatically reduced fluorescence intensity, indicative of substantial suppression of fibril formation. Notably, the fluorescence intensity of the Aβ40 + 3b mixture is nearly identical to that of 3b alone, indicating that no additional fluorescence arises from ThT interaction with fibrillar species in solution. All CD incubation mixtures (collectively presented in Fig. 4B) generated similar results, demonstrating that in the presence of the newly synthesized derivatives the fluorescence signal is suppressed to baseline levels.

The excellent agreement between the ThT results and the CD data confirms that the new derivatives effectively interfere with the progression of Aβ40 from soluble monomers to typical amyloid fibrils.

Collectively, the ESI-HRMS, circular dichroism, and thioflavin-T fluorescence assays provide convergent evidence that the newly synthesized HT derivatives directly modulate Aβ aggregation. ESI-HRMS revealed the formation of stable non-covalent complexes between Aβ and the tested compounds, with 3b exhibiting the highest complexation strength. The CD and ThT data revealed that all tested derivatives suppressed the formation of amyloid fibrils, through direct interactions with Aβ that interfere with the structural rearrangements required for Aβ fibrillization. The strong agreement among these orthogonal techniques identifies 3b as the most prominent compound in this series and supports a mechanism in which direct peptide–ligand interactions interfere with the structural rearrangements required for Aβ fibrillization.

### 2.4 Biological evaluation

#### 2.4.1 *In vitro* antioxidant activity

Given the close connection between oxidative stress and Aβ aggregation in Alzheimer's disease, the antioxidant properties of the compounds were evaluated using a panel of complementary assays, each probing a distinct mechanism relevant to neuronal protection (Tables 3 and 4). In the DPPH free-radical scavenging assay, several compounds demonstrated a markedly enhanced ability to neutralize stable free radicals. As shown in Table 3, compounds 3a, 3c, 3d, 3e, and 5f exhibited significantly lower EC_50_ values than hydroxytyrosol, reflecting superior radical-scavenging activity that may help mitigate oxidative stress associated with Aβ-induced neurotoxicity. The potential of the compounds to inhibit enzymatic lipid peroxidation was assessed through lipoxygenase activity. At 200 μM, hydroxytyrosol produced only modest (∼20%) inhibition, with no further increase at higher concentrations, whereas compounds 4d and 5c displayed stronger inhibitory effects, indicating an improved capacity to curb the enzymatic generation of lipid peroxides implicated in oxidative neuronal damage.

**Table 3:** DPPH radical scavenging activity of the synthesized compounds. Results are expressed as mean ± standard deviation (SD) of at least three independent experiments performed in duplicate

	% scavenging of DPPH mean ± SD					
	Concentration of the synthesized compounds					
Cpd.	1 μM	5 μM	15 μM	35 μM	50 μM	EC_50_ (μM)
3a	4.33 ± 2.61^a^	16.47 ± 3.83^a^	20.68 ± 12.31	45.56 ± 3.63^a^	79.34 ± 6.30^a^	33.27 ± 5.77^a^
3b	3.57 ± 1.86^a^	8.08 ± 3.80	16.58 ± 1.96	23.99 ± 5.93	35.21 ± 8.41^a^	72.94 ± 13.32
3c	10.67 ± 3.17^a^	18.93 ± 1.90^a^	36.89 ± 5.25^a^	45.35 ± 4.52^a^	80.11 ± 4.97^a^	30.55 ± 5.59^a^
3d	8.91 ± 3.33^a^	19.10 ± 5.22^a^	42.92 ± 4.21^a^	63.47 ± 4.29^a^	83.44 ± 2.16^a^	26.01 ± 3.77^a^
3e	0.56 ± 3.45	16.78 ± 5.88^a^	37.12 ± 3.57^a^	55.21 ± 2.55^a^	81.91 ± 2.59^a^	29.23 ± 4.15^a^
3f	2.18 ± 5.75^a^	21.78 ± 2.41^a^	48.79 ± 2.00^a^	67.19 ± 2.33^a^	82.69 ± 3.29^a^	25.04 ± 5.60^a^
4b	4.36 ± 1.56^a^	11.69 ± 2.44	20.99 ± 2.45	28.62 ± 5.32	64.87 ± 4.19^a^	43.40 ± 8.37
4c	6.90 ± 3.05^a^	8.26 ± 2.58	18.50 ± 3.59	25.46 ± 3.68	60.59 ± 6.25^a^	47.49 ± 9.99
4d	7.54 ± 2.37^a^	3.90 ± 7.85	23.84 ± 5.26	26.70 ± 3.68	50.88 ± 3.20^a^	52.67 ± 12.19
4e	0.89 ± 2.36	10.46 ± 3.93	22.04 ± 0.96	36.42 ± 4.77^a^	71.24 ± 3.67^a^	38.29 ± 5.36
4f	4.58 ± 2.54^a^	9.41 ± 4.35	21.69 ± 2.83	29.09 ± 1.92	69.41 ± 6.66^a^	41.06 ± 8.25
5a	−2.68 ± 2.05	7.51 ± 1.74	23.36 ± 2.69	36.63 ± 14.45^a^	68.88 ± 5.20^a^	38.88 ± 6.86
5b	9.03 ± 1.65^a^	7.71 ± 1.05	21.25 ± 0.98	23.40 ± 3.35	66.39 ± 2.36^a^	44.58 ± 11.36
5c	1.17 ± 2.67^a^	9.56 ± 4.52	16.22 ± 4.08	29.05 ± 4.03	60.07 ± 3.87^a^	46.08 ± 6.53
5d	0.48 ± 4.11	10.45 ± 4.52	24.27 ± 6.15^a^	32.16 ± 5.89	71.62 ± 3.73^a^	38.93 ± 7.58
5e	2.93 ± 2.50^a^	9.06 ± 2.67	29.16 ± 2.30^a^	41.93 ± 5.48^a^	80.12 ± 2.48^a^	33.61 ± 4.92^a^
5f	3.07 ± 2.86^a^	5.04 ± 2.65	19.30 ± 2.79	24.83 ± 3.91	51.42 ± 7.17	53.56 ± 9.25
HT	−7.26 ± 4.84	3.73 ± 3.78	14.84 ± 2.64	22.56 ± 3.75	48.81 ± 2.91	55.00 ± 8.55

a *p* < 0.05 compared to hydroxytyrosol (HT) based on one way ANOVA.

**Table 4:** Inhibitory activity of the synthesized compounds against lipoxygenase and Cu-induced serum oxidation

	% inhibition LOX	Lagtime (min)
Control	—	39.16 ± 2.05
3a	7.15 ± 4.06^a^	58.74 ± 6.02
3b	−8.16 ± 2.30^a^	48.13 ± 9.04^b^
3c	17.12 ± 3.46	59.16 ± 7.50
3d	12.92 ± 0.43^a^	81.35 ± 6.66
3e	3.93 ± 0.09^a^	141.73 ± 6.09^b^
3f	16.02 ± 5.50	142.26 ± 19.32^b^
4b	21.81 ± 4.45	80.34 ± 7.50
4c	19.68 ± 5.88	81.55 ± 6.97
4d	45.32 ± 2.23^a^	77.44 ± 6.41
4e	23.19 ± 4.24	91.85 ± 7.36^b^
4f	25.78 ± 3.82	85.71 ± 5.81^b^
5a	−11.86 ± 2.97^a^	79.34 ± 6.94
5b	−3.26 ± 3.74^a^	70.79 ± 7.69
5c	36.88 ± 5.11^a^	88.51 ± 5.77^b^
5d	−3.89 ± 4.16^a^	107.32 ± 10.22^b^
5e	0.55 ± 0.58^a^	81.36 ± 9.46
5f	3.26 ± 1.76^a^	65.96 ± 2.23
HT	19.95 ± 4.31	67.36 ± 3.45

a *p* < 0.000.

b *p* < 0.005 compared to hydroxytyrosol (HT) based on one way ANOVA.

Finally, the compounds were evaluated for their ability to protect serum lipoproteins against Cu^2+^-induced oxidation, a model of physiologically relevant oxidative processes (Table 4). Hydroxytyrosol prolonged the oxidation lag phase by approximately 30 minutes at 200 μM, while derivatives 3b, 3e, 3f, 4e, 5c, and 5d produced substantially longer lag times, demonstrating enhanced protection against oxidative modification of serum lipoproteins, particularly LDL. Collectively, these results highlight that the tested derivatives possess robust antioxidant activity across multiple mechanisms, which may complement their anti-aggregation effects and contribute to neuroprotection in the context of AD.

#### 2.4.2 *In vivo* validation of compound 3b reveals protection against amyloid-β-induced dysfunction

Collectively, biophysical and biochemical evaluation of the HT derivatives identified compound 3b as the most promising candidate. In ESI-HRMS, CD, and ThT assays, 3b exhibited the highest affinity and specificity toward Aβ and most effectively inhibited β-sheet-rich fibril formation, maintaining Aβ in a predominantly random-coil conformation. However, even though fibril formation is suppressed, soluble toxic species, like Aβ oligomers, may still be present in solution. In addition to its strong *in vitro* performance, the dimethoxy and α-oxo substitutions of 3b enhance chemical stability and metabolic robustness compared with the related analogs 4 and 5, providing a data- and chemically-driven rationale for its further *in vivo* evaluation using *C. elegans* models of amyloid-β toxicity.

To evaluate the therapeutic potential of 3b (20 μM) at the organismal level, we employed two well-characterized *C. elegans* strains that model Aβ–associated toxicity. The GRU102 strain (genotype: *gnaIs2*[*p*_myo-2_YFP; *p*_unc-119_Aβ_1–42_]) expresses human Aβ(1–42) pan-neuronally and exhibits impaired neuromuscular and sensorimotor behavior, recapitulating key aspects of Alzheimer's disease-like pathology. As a genetic control, we used the GRU101 strain (genotype: *gnaIs1*[*p*_myo-2_YFP]), which lacks Aβ expression and displays a wild-type phenotype with YFP restricted to the pharynx, as previously described.

**Fig. 5:**
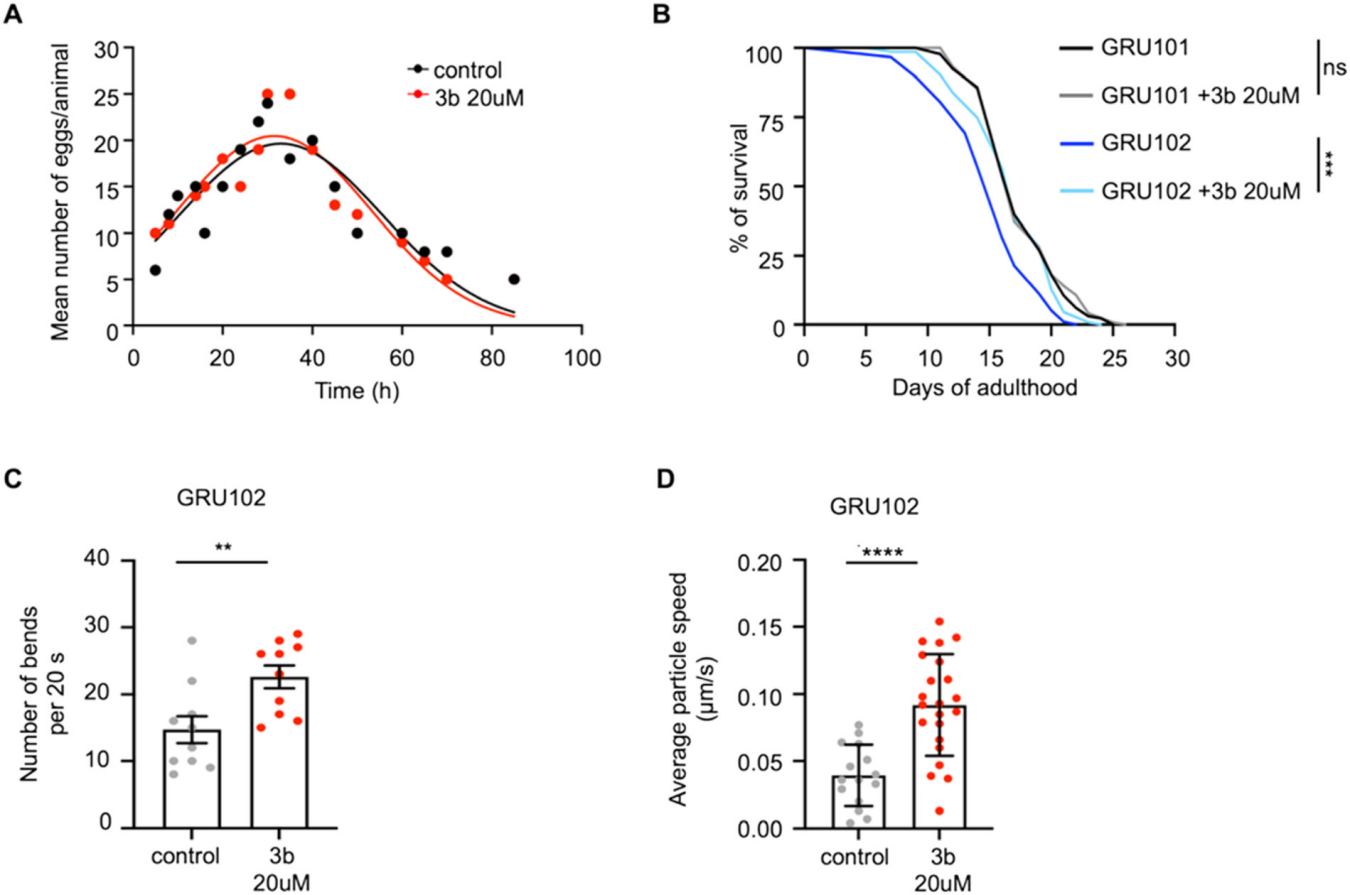
Compound 3b is non-toxic and alleviates amyloid-β-induced phenotypes in *C. elegans*. (A) 3b supplementation does not affect egg production compared to untreated controls, indicating the absence of reproductive toxicity. Data are shown as mean number of eggs per animal over time. (B) Survival analysis of control (GRU101) and amyloid-β-expressing (GRU102) nematodes in the presence or absence of 3b (20 μM). 3b treatment does not alter the lifespan of the GRU101 control strain, whereas it significantly rescued the reduced lifespan of GRU102 animals expressing pan-neuronal Aβ(1–42). Statistical significance is assessed using the log-rank (Mantel–Cox) test (***p < 0.001; ns, not significant). (C) Locomotor performance of GRU102 animals is assessed by swimming (thrashing) assay. 3b-treated worms display a significant increase in the number of body bends per 20 s compared to untreated controls, indicating improved neuromuscular function (**p < 0.01). (D) Crawling speed of GRU102 nematodes on solid media. 3b supplementation significantly increases average locomotion speed relative to untreated animals, reflecting enhanced sensorimotor performance (****p < 0.0001).

To exclude potential toxic effects of 3b, wild-type (N2) nematodes were treated with 20 μM 3b and assessed for developmental progression and reproductive output. 3b supplementation did not affect larval development, growth rate, or egg-laying capacity, and total progeny production remained unchanged compared to controls (Fig. 5A). These data indicate that 3b is well tolerated and does not interfere with normal *C. elegans* development, growth, or reproductive fitness.

We next evaluated the effects of 3b in the GRU101 and GRU102 strains. 3b treatment did not alter the lifespan of the GRU101 control strain, indicating an absence of nonspecific longevity effects. In contrast, 3b significantly rescued the reduced lifespan of Aβ-expressing GRU102 animals (Fig. 5B), demonstrating a selective protective effect under proteotoxic conditions. To further assess functional outcomes, we analyzed locomotor performance in GRU102 animals. 3b-treated worms exhibited a marked improvement in swimming behavior, as evidenced by an increased number of body bends in liquid media (Fig. 5C), and displayed enhanced motility on solid media, reflected by increased crawling speed (Fig. 5D).

Collectively, these findings demonstrate that 3b is non-toxic *in vivo* and confers significant functional and lifespan benefits in an amyloid-β-dependent *C. elegans* model, highlighting its therapeutic potential against amyloid-induced toxicity and neurodegenerative decline.

The neuroprotective effects of compound 3b, including inhibition of Aβ aggregation and reduction of oxidative stress, act through complementary mechanisms to clinically approved drugs such as donepezil or memantine, which primarily target synaptic signaling. This multifunctional activity highlights the potential of hydroxytyrosol-derived analogs as adjuncts to existing therapies. Together, these findings support further development of 3b as part of combination strategies for multifactorial intervention in AD.

## 3. Conclusion

In this work, we developed a series of hydroxytyrosol-based compounds designed to overcome the limited stability of oleuropein while enhancing interactions with β-amyloid peptides. The synthetic strategy yielded structurally diverse esters that were readily prepared in high yields and thoroughly characterized. Biophysical analyses revealed that several compounds form strong non-covalent complexes with Aβ40 and effectively inhibit its early aggregation steps, as confirmed by ESI-MS, CD spectroscopy, and thioflavin-T fluorescence assay. These compounds preserve Aβ in a predominantly random-coil conformation and prevent the formation of β-sheet-rich fibrils. Many of the analogs also display superior antioxidant activity compared to hydroxytyrosol, suggesting a dual mode of action that addresses both amyloid aggregation and oxidative stress – two mutually reinforcing contributors to AD pathology. The use of Aβ40 in these *in vitro* studies was motivated by its slower aggregation kinetics, which enable more controlled biophysical characterization.^41^

The biological relevance of these effects was validated *in vivo* using *C. elegans* models of amyloid-β toxicity. The lead compound 3b was well tolerated in wild-type animals, with no detectable effects on development, growth, or reproductive capacity. In contrast, 3b ameliorated lifespan shortening and locomotor impairments in transgenic nematodes expressing human Aβ42, a more aggregation-prone and pathologically relevant isoform,^42^ providing functional evidence of protection against amyloid-induced proteotoxicity at the organismal level. Taken together, these findings identify a set of stable, multifunctional hydroxytyrosol derivatives with improved anti-amyloidogenic and antioxidant properties. Compound 3b represents promising lead for further optimization and development as a novel therapeutic agent targeting the multifactorial mechanisms underlying Alzheimer's disease.

## 4. Experimental section

### 4.1 Chemistry

All commercially available reagents and solvents were purchased from Alfa Aesar (Ward Hill, Massachusetts, MA, USA) and used without any further purification. Melting points were determined on Büchi apparatus and were uncorrected. 1D (^1^H NMR, ^13^C NMR) and 2D (COSY, NOESY, HMBC, HSQC-DEPT135) NMR spectra were carried out on 400 or 600 MHz Bruker spectrometers respectively Avance™ DRX and III instruments (Bruker BioSpin GmbH – Rheinstetten, Germany). Chemical shifts (*δ*) are expressed in ppm while coupling constants (*J*) in Hz. Flash chromatography was performed on Merck silica gel (40–63 μm) with the indicated solvent system using gradients of increasing polarity in most cases (Merck KGaA—Darmstadt, Germany). The reactions were monitored by analytical thin-layer chromatography (Merck pre-coated silica gel 60 F254 TLC plates, 0.25-mm layer thickness). Mass spectra were recorded on a UPLC Triple TOF-MS ((UPLC: Acquity of Waters (Milford, MA 01757, USA), SCIEX Triple TOF-MS 5600+ (Framingham, MA 01701, USA)).

#### 2-Chloro-1-(3,4-dihydroxyphenyl)ethan-1-one (2)

A suspension of catechol (1.06 g, 10 mmol, 1) and chloroacetylchloride (0.9 mL, 11 mmol) in phosphorus oxychloride (2.8 mL, 30 mmol) was stirred at 110 °C for 12 hours. After completion of the reaction, the mixture was vacuum evaporated, and the oily residue was poured into ice-water. The resulting precipitate was filtered off, washed with water and dried over CaCl_2_, to afford the title compound 2 (1.71 g, 92%). ^1^H NMR and ^13^C NMR are in agreement with the reported data.^43^

#### 2-(3,4-Dihydroxyphenyl)-2-oxoethyl acetate (3a)

To a solution of compound 2 (1.5 g, 8.04 mmol) in dry DMF (20 mL), anhydrous potassium acetate (1.57 g, 16 mmol) was added, and the mixture was heated at 70 °C for 4 hours. After completion of the reaction, the volatiles were vacuum evaporated, the residue was diluted in EtOAc (80 mL), the organic phase was washed with water (3 × 30 mL), dried over anhydrous Na_2_SO_4_ and concentrated to dryness. Flash chromatography (silica gel) using a mixture of c-Hex/EtOAc 5/1 as the eluent provided 1.58 g (94%) of the title compound 3a. Mp.: 168–170 °C (EtOAc). Yield: 87%. ^1^H NMR and ^13^C NMR are in agreement with the reported data.^44^

#### General synthetic procedure for the synthesis of compounds 3b–e

To a solution of the appropriate acid (0.8 mmol) in dry DMF (2 mL), at 0 °C, sodium hydride (32 mg, 0.8 mmol, 60% in mineral oil) was added under argon. The resulting suspension was stirred at room temperature for 15 min, then cooled at 0 °C. Subsequently, chloride 2 (0.54 mmol) was added, and the resulting suspension was heated at 70 °C for 4 hours. Upon completion of the reaction, the volatiles were vacuum evaporated, the residue was diluted in EtOAc (30 mL), the organic phase was washed with water (3 × 20 mL), dried over anhydrous Na_2_SO_4_ and concentrated to dryness. Flash chromatography (silica gel) using a mixture of c-Hex/EtOAc 4/1 as the eluent provided the title compounds 3b–3e.

##### 2-(3,4-Dihydroxyphenyl)-2-oxoethyl 3,5-dimethoxybenzoate (3b)

Yield: 34%. Mp.: 188–189 °C (EtOAc). ^1^H NMR (400 MHz, DMSO-*d*_6_) *δ* 10.05 (br s, 1H, D_2_O exchang., 3-OH), 9.47 (br s, 1H, D_2_O exchang., 4-OH), 7.42 (dd, *J* = 8.2, 2.0 Hz, 1H, H-6′), 7.36 (d, *J* = 2.0 Hz, 1H, H-2′), 7.13 (d, *J* = 2.0 Hz, 2H, H-2 and H-6), 6.87 (d, *J* = 8.2 Hz, 1H, H-5′), 6.83 (t, *J* = 2.3 Hz, 1H, H-4), 5.60 (s, 2H, CH_2_), 3.82 (s, 6H, 3,5-OCH_3_). ^13^C NMR (151 MHz, DMSO-*d*_6_) *δ* 190.5 (*C*OCH_2_), 164.9 (OCO), 160.5 (C-3 and C-5), 151.5 (C-4′), 145.5 (C-3′), 131.3 (C-1), 125.7 (C-1′), 121.1 (C-6′), 115.3 (C-5′), 114.5 (C-2′), 106.9 (C-2 and C-6), 105.5 (C-4), 66.8 (CH_2_), 55.5 (3,5-OCH_3_). HRMS (ESI) calculated for C_17_H_17_O_7_ + H^+^ [M + H^+^]: 333.0969. Found: 333.0975.

##### 2-(3,4-Dihydroxyphenyl)-2-oxoethyl 3,5-dihydroxybenzoate (3c)

Yield: 44%. Mp.: >250 °C (dec) (EtOAc). ^1^H NMR (400 MHz, DMSO-*d*_6_) *δ* 9.76 (br s, 4H, D_2_O exchang., 3,5-OH and 3′,4′-OH), 7.41 (d, *J* = 8.3 Hz, 1H, H-6′), 7.36 (s, 1H, H-2′), 6.89 (s, 2H, H-2, 6), 6.87 (d, *J* = 8.3 Hz, 1H, H-5′), 6.49 (s, 1H, H-4), 5.55 (s, 2H, CH_2_O). ^13^C NMR (151 MHz, DMSO-*d*_6_) *δ* 190.8 (*C*OCH_2_), 165.4 (OCO), 158.64 (C-3 and C-5), 151.5 (C-4′), 145.6 (C-3′), 131.1 (C-1), 125.9 (C-1′), 121.2 (C-6′), 115.3 (C-5′), 114.6 (C-2′), 107.5 (C-4), 107.4 (C-2 and C-6), 66.5 (CH_2_O). HRMS (ESI) calculated for C_15_H_13_O_7_ + H^+^ [M + H^+^]: 305.0656. Found: 305.0664.

##### 2-(3,4-Dihydroxyphenyl)-2-oxoethyl cyclohexanecarboxylate (3d)

Yield: 86%. Mp.: 140–141 °C (CH_2_Cl_2_). ^1^H NMR (400 MHz, DMSO-*d*_6_) *δ* 9.94 (br s, 1H, D_2_O exchang., 3′-OH), 9.43 (br s, 1H, D_2_O exchang., 4′-OH), 7.35 (d, *J* = 7.8 Hz, 1H, H-6′), 7.30 (s, 1H, H-2′), 6.83 (d, *J* = 7.8 Hz, 1H, H-5′), 5.31 (s, 2H, CH_2_O), 2.50–2.40 (m, 1H, H-1), 1.94–1.83 (m, 2H, H-2 and H-6), 1.77–1.65 (m, 2H, H-3 and H-5), 1.63–1.54 (m, 1H, H-4), 1.48–1.37 (m, 2H, H-2 and H-6), 1.35–1.17 (m, 3H, H-3, H-4, H-5). ^13^C NMR (151 MHz, DMSO-*d*_6_) *δ* 190.7 (*C*OCH_2_), 174.5 (OCO), 151.3 (C-4′), 145.4 (C-3′), 125.8 (C-1′), 121.00 (C-6′), 115.2 (C-5′), 114.5 (C-2′), 65.6 (CH_2_O), 41.9 (C-1), 28.6 (C-2 and C-6), 25.3 (C-4), 24.68 (C-3 and C-5). HRMS (ESI) calculated for C_15_H_19_O_5_ + H^+^ [M + H^+^]: 279.1227. Found: 279.1232.

##### 2-(3,4-Dihydroxyphenyl)-2-oxoethyl 2-cyclohexylacetate (3e)

Yield: 45.7%. Mp.: 142–143 °C (CH_2_Cl_2_–*n*-pentane). ^1^H NMR (400 MHz, DMSO-*d*_*6*_) *δ* 10.01 (br s, 1H, D_2_O exchang., 3′-OH), 9.44 (br s, 1H, D_2_O exchang., 4′-OH), 7.36 (dd, *J* = 8.2, 1.8 Hz, 1H, H-6′), 7.32 (s, 1H, H-2′), 6.84 (d, *J* = 8.2 Hz, 1H, H-5′), 5.32 (s, 2H, CH_2_O), 2.29 (d, *J* = 7.0 Hz, 2H, COCH_2_), 1.81–1.73 (m, 3H, H-1, H-2 and H-6), 1.72–1.57 (m, 3H, H-3, H-4 and H-5), 1.30–1.06 (m, 3H, H-3, H-4 and H-5), 1.05–0.90 (m, 2H, H- 2 and H-6). ^13^C NMR (151 MHz, DMSO-*d*_6_) *δ* 190.7 (*C*OCH_2_), 171.6 (O*C*OCH_2_), 151.3 (C-4′), 145.4 (C-3′), 125.8 (C-1′), 121.0 (C-6′), 115.2 (C-5′), 114.5 (C-2′), 65.7 (CH_2_O), 41.0 (CO*C*H_2_), 34.4 (C-1), 32.3 (C-2 and C-6), 25.7 (C-4), 25.5 (C-3 and C-5). HRMS (ESI) calculated for C_16_H_21_O_5_ + H^+^ [M + H^+^]: 293.1384. Found: 293.1389.

#### General synthetic procedure for the synthesis of compounds 4a–e

To a mixture of compound 3a–e (1 mmol) in CF_3_COOH (3 mmol), Et_3_SiH (2 mmol) was added dropwise, and the mixture was stirred at room temperature for 3 hours. After completion of the reaction, the volatiles were removed under reduced pressure, the residue was diluted in CH_2_Cl_2_ (40 mL), the organic phase was washed with aqueous 10% NaHCO_3_ solution (30 mL), water (3 × 20 mL), dried over anhydrous Na_2_SO_4_ and concentrated to dryness. Flash chromatography on silica gel using a mixture of c-Hex/EtOAc 3/1 as the eluent provided the title compounds 4a–e.

##### 3,4-Dihydroxyphenethyl acetate (4a)

Yield: 64.7%. ^1^H NMR and ^13^C NMR are in agreement with the reported data.^43^

##### 3,4-Dihydroxyphenethyl 3,5-dimethoxybenzoate (4b)

Yield: 77%. Mp.: 108–109 °C (CH_2_Cl_2_/*n*-hexane). ^1^H NMR (400 MHz, CDCl_3_) *δ* 7.18 (d, *J* = 2.3 Hz, 2H, H-2 and H-6), 6.85 (d, *J* = 8.1 Hz, 1H, H-5′), 6.81 (d, *J* = 2.3 Hz, 1H, H-2′), 6.71 (dd, *J* = 8.1, 2.3 Hz, 1H, H-6′), 6.66 (t, *J* = 2.3 Hz, 1H, H-4), 4.47 (t, *J* = 7.0 Hz, 2H, CH_2_C*H*_2_O), 3.83 (s, 6H, 3,5-OCH_3_), 2.96 (t, *J* = 7.0 Hz, 2H, C*H*_2_CH_2_O). ^13^C NMR (151 MHz, CDCl_3_) *δ* 166.7 (CO), 160.6 (C-3 and C-5), 143.7 (C-3′), 142.4 (C-4′), 132.0 (C-1), 130.6 (C-1′), 121.3 (C-6′), 116.1 (C-5′), 115.4 (C-2′), 107.4 (C-2 and C-6), 105.8 (C-4), 65.9 (CH_2_*C*H_2_O), 55.4 (3,5-OCH_3_), 34.6 (*C*H_2_CH_2_O). HRMS (ESI) calculated for C_17_H_19_O_6_ + H^+^ [M + H^+^]: 319.1176. Found: 319.1173.

##### 3,4-Dihydroxyphenethyl 3,5-dihydroxybenzoate (4c)

Yield: 84%. Mp.: 110–111 °C (EtOAc/c-Hex). ^1^H NMR (600 MHz, DMSO-*d*_6_) *δ* 9.64 (br s, 2H, D_2_O exchang., 3,5-O*H*), 8.79 (br s, 1H, D_2_O exchang., 4′-O*H*), 8.75 (br s, 1H, D_2_O exchang., 3′-O*H*), 6.80 (d, *J* = 2.1 Hz, 2H, H-2, 6), 6.67 (d, *J* = 7.9 Hz, 1H, H-5′), 6.64 (d, *J* = 2.1 Hz, 1H, H-2′), 6.53 (dd, *J* = 7.9, Hz, 2.1 Hz, 1H, H-6′), 6.44 (t, *J* = 2.3 Hz, 1H, H-4), 4.32 (t, *J* = 6.8 Hz, 2H, CH_2_C*H*_2_O), 2.81 (t, *J* = 6.8 Hz, 2H, C*H*_2_CH_2_O). ^13^C NMR (151 MHz, DMSO-*d*_6_) *δ* 165.7 (CO), 158.5 (C-3 and C-5), 145.1 (C-3′), 143.7 (C-4′), 131.5 (C-1), 128.7 (C-1′), 119.5 (C-6′), 116.2 (C-5′), 115.5 (C-2′), 107.1 (C-2, C-4, C-6), 65.4 (CH_2_*C*H_2_O), 33.8 (*C*H_2_CH_2_O).^45^ HRMS (ESI) calculated for C_15_H_15_O_6_ + H^+^ [M + H^+^]: 291.0863. Found: 291.0858.

##### 3,4-Dihydroxyphenethyl cyclohexanecarboxylate (4d)

Yield: 66.7%. Viscus oil. ^1^H NMR (600 MHz, CDCl_3_) *δ* 6.81 (d, *J* = 8.0 Hz, 1H, H-5′), 6.76 (s, 1H, H-2′), 6.63 (d, *J* = 8.0 Hz, 1H, H-6′), 4.25 (t, *J* = 6.9 Hz, 2H, CH_2_C*H*_2_O), 2.81 (t, *J* = 6.9 Hz, 2H, C*H*_2_CH_2_O), 2.40–2.27 (m, 1H, H-1), 1.91–1.86 (m, 2H, H-2 and H-6), 1.81–1.70 (m, 2H, H-3 and H-5), 1.69–1.61 (m, 1H, H-4), 1.51–1.40 (m, 2H, H-2 and H-6), 1.26 (m, 3H, H-3, H-4 and H-5). ^13^C NMR (151 MHz, CDCl_3_) *δ* 177.3 (CO), 143.9 (C-3′), 142.5 (C-4′), 130.3 (C-1′), 121.2 (C-6′), 116.0 (C-5′), 115.4 (C-2′), 65.2 (CH_2_*C*H_2_O), 43.3 (C-1), 34.4 (*C*H_2_CH_2_O), 28.9 (C-2 and C-6), 25.7 (C-4), 25.4 (C-3 and C-5). HRMS (ESI) calculated for C_15_H_21_O_4_ + H^+^ [M + H^+^]: 265.1434. Found: 265.1431.

##### 3,4-Dihydroxyphenethyl 2-cyclohexylacetate (4e)

Yield: 95%. Viscus oil. ^1^H NMR (400 MHz, CDCl_3_) *δ* 6.81 (d, *J* = 8.0 Hz, 1H, H-5′), 6.75 (d, *J* = 1.8 Hz, 1H, H-2′), 6.62 (dd, *J* = 8.0, 1.8 Hz, 1H, H-6′), 4.27 (t, *J* = 7.0 Hz, 2H, CH_2_C*H*_2_O), 2.84 (t, *J* = 7.0 Hz, 2H, C*H*_2_CH_2_O), 2.19 (d, *J* = 7.1 Hz, 2H, COCH_2_), 1.82–1.60 (m, 6H, H-1, H-2, H-3, H-4, H-5 and H-6), 1.31–1.08 (m, 3H, H-3, H-4 and H-5), 0.98–0.91 (m, 2H, H-2 and H-6). ^13^C NMR (151 MHz, CDCl_3_) *δ* 174.3 (CO), 143.9 (C-3′), 142.6 (C-4′), 130.2 (C-1′), 121.1 (C-6′), 115.8 (C-5′), 115.3 (C-2′), 65.2 (CH_2_*C*H_2_O), 42.3 (CO*C*H_2_), 34.9 (*C*H_2_CH_2_O), 34.4 (C-1), 32.9 (C-2 and C-6), 26.9 (C-4), 26.1 (C-3 and C-5). HRMS (ESI) calculated for C_16_H_23_O_4_ + H^+^ [M + H^+^]: 279.1591. Found: 279.1598.

#### General synthetic procedure for the synthesis of compounds 5a–e

A suspension of the appropriate compound 3a–e (2.38 mmol) and Pd/C 10% (30 mg) in *t*-BuOH (20 mL) was hydrogenated at 50 psi, at room temperature, for 4 hours. After completion of the reaction, the reaction mixture was filtered through a Celite pad, and the filtrate was vacuum evaporated. The residue was recrystallized, to furnish the title compounds 5a–e.

##### 2-(3,4-Dihydroxyphenyl)-2-hydroxyethyl acetate (5a)

Yield: 92%. Mp.: 134–135 °C (CH_2_Cl_2_/*n*-pentane). ^1^H NMR (400 MHz, DMSO-*d*_6_) *δ* 8.85 (br s, 1H, D_2_O exchang., 3-OH), 8.78 (br s, 1H, D_2_O exchang., 4-OH), 6.75 (s, 1H, H-2), 6.66 (d, *J* = 8.0 Hz, 1H, H-5), 6.58 (d, *J* = 8.0 Hz, 1H, H-6), 5.35 (d, *J* = 4.0 Hz, 1H, CHO*H*), 4.59–4.57 (m, 1H, C*H*CH_2_), 3.94 (d, *J* = 8.0 Hz, 2H, CHC*H*_2_), 1.99 (s, 3H, CH_3_). ^13^C NMR (151 MHz, DMSO-*d*_6_) *δ* 170.6 (CO), 145.4 (C-3), 144.8 (C-4), 133.2 (C-1), 117.7 (C-6), 115.5 (C-5), 113.9 (C-2), 70.3 (*C*HCH_2_), 69.2 (CH*C*H_2_), 21.1 (CH_3_).^46^ HRMS (ESI) calculated for C_10_H_13_O_5_ + H^+^ [M + H^+^]: 213.0757. Found: 213.0764.

##### 2-(3,4-Dihydroxyphenyl)-2-hydroxyethyl 3,5-dimethoxybenzoate (5b)

Yield: 78.6%. Mp.: 118–119 °C (ethanol). ^1^H NMR (600 MHz, DMSO-*d*_6_) *δ* 8.82 (br s, 1H, D_2_O exchang., 3′-OH), 8.77 (br s, 1H, D_2_O exchang., 4′-OH), 7.06 (d, *J* = 1.8 Hz, 2H, H-2 and H-6), 6.83 (d, *J* = 1.8 Hz, 1H, H-2′), 6.77 (t, *J* = 2.3 Hz, 1H, H-4), 6.69 (d, *J* = 8.0 Hz, 1H, H-5′), 6.66 (dd, *J* = 8.0, 1.8 Hz, 1H, H-6′), 5.49 (d, *J* = 4.4 Hz, 1H, CHO*H*), 4.75–4.73 (m, 1H, C*H*CH_2_), 4.21 (d, *J* = 8.0 Hz, 2H, CHC*H*_2_), 3.79 (s, 6H, 3,5-OCH_3_). ^13^C NMR (151 MHz, DMSO-*d*_6_) *δ* 165.3 (CO), 160.4 (C-3 and C-5), 145.0 (C-3′), 144.5 (C-4′), 132.8 (C-1′), 131.8 (C-1), 117.1 (C-6′), 115.1 (C-5′), 113.7 (C-2′), 106.8 (C-2 and C-6), 105.2 (C-4), 70.0 (*C*HCH_2_), 69.7 (CH*C*H_2_), 55.5 (3,5-OCH_3_). HRMS (ESI) calculated for C_17_H_19_O_7_ + H^+^ [M + H^+^]: 335.1125. Found: 335.1128.

##### 2-(3,4-Dihydroxyphenyl)-2-hydroxyethyl 3,5-dihydroxybenzoate (5c)

Yield: 76.2%. Mp.: 181–182 °C (CH_2_Cl_2_). ^1^H NMR (400 MHz, DMSO-*d*_6_) *δ* (ppm): 9.64 (br s, 2H, D_2_O exchang., 3,5-O*H*), 8.87 (br s, 1H, D_2_O exchang., 3′-O*H*), 8.82 (br s, 1H, D_2_O exchang., 4′-O*H*), 6.83 (s, 2H, H-2, 6), 6.81 (s, 1H, H-2′), 6.69 (d, *J* = 8.0 Hz, 1H, H-5′), 6.65 (d, *J* = 8.0 Hz, 1H, H-6′), 6.44 (s, 1H, H-4), 5.47 (d, *J* = 3.8 Hz, 1H, CHO*H*), 4.75–4.65 (m, 1H, C*H*CH_2_), 4.20–4.10 (m, 2H, CHC*H*_2_). ^13^C NMR (151 MHz, DMSO-*d*_6_) *δ* (ppm): 166.00 (*C*O), 158.46 (C-3,5), 145.00 (C-3′), 144.67 (C-4′), 132.82 (C-1′), 131.30 (C-1), 116.99 (C-6′), 115.04 (C-5′), 113.41 (C-2′), 107.26 (C-2, 4, 6), 69.93 (*C*HCH_2_), 69.23 (CH*C*H_2_). HRMS (ESI) calculated for C_15_H_15_O_7_ + H^+^ [M + H^+^]: 307.0812. Found: 307.0805.

##### 2-(3,4-Dihydroxyphenyl)-2-hydroxyethyl cyclohexanecarboxylate (5d)

Yield: 80%. Mp: 141–142 °C (CHCl_3_/*n*-pentane). ^1^H NMR (400 MHz, DMSO-*d*_6_) *δ* 8.84 (br s, 1H, D_2_O exchang., 3′-OH), 8.77 (br s, 1H, D_2_O exchang., 4′-OH), 6.75 (s, 1H, H-2′), 6.67 (d, *J* = 8.0 Hz, 1H, H-5′), 6.58 (d, *J* = 8.0 Hz, 1H, H-6′), 5.34 (d, *J* = 3.8 Hz, 1H, CHO*H*), 4.62–4.51 (m, 1H, C*H*CH_2_), 4.04–3.90 (m, 2H, CHC*H*_2_), 2.34–2.21 (m, 1H, H-1), 1.88–1.71 (m, 2H, H-2 and H-6), 1.70–1.61 (m, 2H, H-3 and H-5), 1.60–1.54 (m, 1H, H-4), 1.40–1.11 (m, 5H, H-2, H-3, H-4, H-5, H-6). ^13^C NMR (151 MHz, DMSO-*d*_6_) *δ* 174.8 (CO), 144.9 (C-3′), 144.5 (C-4′), 133.0 (C-1′), 117.1 (C-6′), 115.1 (C-5′), 113.7 (C-2′), 70.1 (*C*HCH_2_), 68.5 (CH*C*H_2_), 42.1 (C-1), 28.5 (C-2 and C-6), 25.3 (C-4), 24.8 (C-3 and C-5). HRMS (ESI) calculated for C_15_H_21_O_5_ + H^+^ [M + H^+^]: 281.1384. Found: 281.1389.

##### 2-(3,4-Dihydroxyphenyl)-2-hydroxyethyl 2-cyclohexylacetate (5e)

Yield: 74%. Mp.: 167–168 °C (CH_2_Cl_2_). ^1^H NMR (400 MHz, DMSO-*d*_6_) *δ* 8.80 (br s, 2H, D_2_O exchang., 3′, 4′-OH), 6.74 (s, 1H, H-2′), 6.66 (d, *J* = 8.0 Hz, 1H, H-5′), 6.57 (d, *J* = 8.0 Hz, 1H, H-6′), 5.31 (d, *J* = 4.2 Hz, 1H, CHO*H*), 4.55 (t, *J* = 5.4 Hz, 1H, C*H*CH_2_), 3.91–4.00 (m, 2H, CHC*H*_2_), 2.13 (d, *J* = 7.1 Hz, 2H, COCH_2_), 1.51–1.69 (m, 6H, H-1, H-2, H-3, H-4, H-5 and H-6), 1.25–1.01 (m, 3H, H-3, H-4 and H-5), 0.95–0.81 (m, 2H, H-2 and H-6). ^13^C NMR (151 MHz, DMSO-*d*_6_) *δ* 171.81 (CO), 144.7 (C-3′), 144.4 (C-4′), 133.1 (C-1′), 116.8 (C-6′), 114.9 (C-5′), 113.5 (C-2′), 69.9 (*C*HCH_2_), 68.4 (CH*C*H_2_), 41.4 (CO*C*H_2_), 34.2 (C-1), 32.3 (C-2 and C-6), 25.6 (C-4), 25.5 (C-3 and C-5). HRMS (ESI) calculated for C_16_H_23_O_5_ + H^+^ [M + H^+^]: 295.1540. Found: 295.1547.

### 4.2 Mass spectrometry assay

All experiments were performed in triplicate employing a Waters Q-ToF premiere mass spectrometer using the ESI ion source in the positive ionization mode. The parameters used are tabulated in Table S1. The samples were infused *via* a metal needle capillary with a constant flow rate of 3.00 μL min^−1^. The solutions infused were prepared as follows: 50 μL of an Aβ(1–40) (200 μM) were diluted with 40 μL ultra-pure water and 100 μL of ACN. Consequently, 10 μL of each compound (1 mM) were added. The ratio of analyzed compound to Aβ was kept to 1 : 1 throughout all experiments. The collision energy employed for the MS/MS experiments was scanned from 5 to 30 V and the fragmentation of the complex has been monitored and calculated assuming that the highest intensity of the complex was achieved in the lower collision energy. The observed intensity has been expressed as the % of the respective intensity at the lowest collision energy, *i.e.*, that of the 5 V.

### 4.3 QSAR methodology

The experimentally determined complexation capability of the HT analogs was used as the dependent variable for QSAR model construction. Molecular structures were converted to SMILES format and optimized using the MOPAC 22.10 as downloaded from GitHub https://github.com/openmopac/mopac/releases/tag/v22.1.0). The PM7 basis set has been used for the geometry optimization. Molecular descriptors were calculated using PaDEL and the OCHEM descriptor calculation platform (https://ochem.eu/descriptorscalculator/show.do). Multivariate statistical analysis was performed using SIMCA 14 (Umetrics, Umeå, Sweden) employing the partial least squares regression (PLS-R) approach. Descriptor data were Pareto-scaled prior to model development, and model validation was performed using internal cross-validation, permutation testing, and CV-ANOVA analysis.

### 4.4 Preparation of amyloid peptide (Aβ) solutions

Aβ40 was purchased from Eurogentec (Belgium) and was gently dissolved in Milli-Q water to reach a concentration of 100 μM and then was diluted in phosphate buffer (PB, 10 mM, pH 7.33) to achieve final concentration of 50 μM. Proper amount of the compound solutions (10 mM in CH_3_OH) was added to the Aβ solutions to achieve a final concentration of 50 μM.^47^

### 4.5 Circular dichroism (CD) measurements

The structural changes of a 50 μM solution of plain Aβ40 in H_2_O/CH_3_OH 1% obtained over a 25-day period were monitored for 25 days. CD spectra were recorded on a JASCO J-715 spectropolarimeter (Jasco Co., Tokyo, Japan), at 33 °C in the range of 190–260 nm with a 1 mm path length quartz cuvette. Each spectrum was the average of three scans at a speed of 100 nm min^−1^ and a resolution of 0.5 nm. The analysis of CD data was performed with the OriginPro 9 program.

### 4.6 Thioflavin T fluorescence assay

Solutions of a 25 day prepared Aβ40 stock solution in the absence and presence of compounds were diluted with PB (10 mM, pH 7.33) to achieve a final peptide concentration of 25 μM. A stock solution of ThT (Sigma, St. Louis, MO) in PB (10 mM, pH 7.33) was subsequently added to reach a 5 μM final concentration of ThT. Fluorescence was monitored after excitation at 440 nm (EM slit = 2.5 nm, PMT voltage 700 V, response 0.4 s) with a HITACHI F-2500 spectrofluorometer and the analysis of fluorescence data was performed using the OriginPro 9 program.

### 4.7 Soybean lipoxygenase inhibition assay

The assay was performed according to a previously described procedure,^48^ with some modifications. The incubation mixture consisted of the appropriate amount (up to 40 μL) of the sample solution in the chosen solvent (water or dimethyl sulfoxide), 5 μL of the enzyme solution (60 units per μL in boric acid buffer) and 165 μL of 0.2 M boric acid buffer, pH 9.0. After incubation at room temperature for 5 min in the dark, the reaction was started by adding 40 μL of linoleic acid solution (937 μM in 25 μL dimethyl sulfoxide and 1225 μL buffer). The total volume of the reaction solution was 250 μL and the final concentration of linoleic acid was 150 μM in the reaction mixture. The conversion of linoleic acid to 13-hydroperoxylinoleic acid was recorded at 234 nm (room temperature) and compared to the appropriate standard solution, which did not contain the extracts. Every sample was tested at least in duplicate. The results were expressed as the % inhibition of the lipoxygenase activity.

### 4.8 DPPH radical-scavenging activity

The DPPH assay was used to measure the free radical-scavenging capacity of the molecules, according to a previously reported method,^49^ with modifications. Briefly, several amounts of each molecule diluted in ethanol in order to obtain final concentratio of 1 μM, 5 μM, 15 μM, 35 μM and 50 μM). Then, was mixed with 35 μL of a freshly prepared ethanolic solution of 0.4 mg mL^−1^ DPPH in microplate wells. The total volume of the assay was 0.2 mL. The solutions were incubated at 37 °C for 30 min and the absorbance were measured at 492 nm with a microplate reader. The percentage of scavenging was calculated by the formula: % scavenging = 100 × *A*_molecule_/*A*_control_, where *A*_control_ is the absorbance of the control without the molecule and *A*_molecule_ is the absorbance with the molecule. The EC_50_ values, which reflect the equivalent concentration able to scavenge 50% of the DPPH radicals, were estimated by the plot of % scavenging towards concentration and were expressed as μM. Every sample was tested at least in triplicate.

### 4.9 Inhibition of Cu^2+^ induced serum oxidation

Fasting venous blood was collected in tubes without an anticoagulant and was left for 40 min at room temperature to clot. The blood was centrifuged at 1500 × *g* for 10 min. The supernatant was collected and diluted with phosphate buffer solution at 1/12 ratio. The reaction mixture contained samples in ethanol or water, 20 μL of human serum and 230 μL of copper sulfate 20 μM. The conjugated dienes' absorption was measured continuously for 6 h at 245 nm at 37 °C. The analysis was conducted with the use of a microplate spectrophotometer (BioTek PowerWave XS2. Results are expressed in minutes (lag time). Every sample was tested at least in triplicate.

### 4.10 Statistical analysis

Normality was tested with the Kolmogorov–Smirnoff criterion. All the variables had normal distribution and the results were expressed as mean values ± SD. For the comparison of the molecules one-way analysis of variance (ANOVA) was performed and a *post hoc* analysis was carried out, where appropriate, with the Bonferroni correction.

### 4.11 *C. elegans* strains and maintenance

All *C. elegans* strains were maintained on NGM plates seeded with *Escherichia coli* OP50 at 20 °C using standard procedures.^50^ N2, GRU101:, GRU102:

#### *C. elegans* lifespan assay

Lifespan assays were performed at 20 °C. Age-synchronized worms were maintained on nematode growth medium (NGM) plates seeded with *E. coli* OP50 that had been UV-killed prior to compound administration to prevent bacterial metabolism of 3b. 3b was applied directly onto the UV-killed bacterial lawn at a final concentration of 20 μM and allowed to be absorbed before worm transfer. Vehicle control plates were prepared in parallel by applying the corresponding solvent (ddH_2_O) used for 3b delivery onto the UV-killed bacterial lawn at the same final volume, ensuring identical handling and exposure conditions across all experimental groups.

Animals were transferred to freshly prepared plates daily during the first week of adulthood and every other day thereafter to ensure a consistent food source and minimize contamination. Worms that died from non-age-related causes, including desiccation at the plate edge, internal hatching (bagging), vulval rupture, or loss during transfers, were censored from the analysis. For each condition, more than 150 worms were distributed across five independent plates, with at least 20 animals per plate. Survival was scored daily. Lifespan data were analyzed using Kaplan–Meier survival analysis, and statistical comparisons were performed using GraphPad Prism.

#### Egg-laying assay

Egg-laying was assessed using age-synchronized day-1 adult *C. elegans* maintained individually on NGM plates with or without 3b (20 μM) supplementation. For each condition, 10 worms were placed on freshly seeded OP50 plates (one worm per plate) at the start of the assay and monitored for a total duration of 90 hours.

At 8 hour intervals, each worm was transferred to a new freshly seeded OP50 plate corresponding to the same experimental condition (3b-treated or control). Eggs laid during each interval were counted on the plate from which the worm was removed and recorded as eggs per worm per time point. Cumulative egg production per worm over the 90-hour period was calculated by summing egg counts across all time intervals. Worms that were lost, exhibited internal hatching (bagging), or showed vulval rupture were excluded from the analysis.

#### Locomotion assessment: thrashing assay

Thrashing behavior was assessed using age-synchronized day-8 adult worms. Individual animals were transferred into 15 μL of M9 buffer placed on a glass slide. Locomotion was quantified by counting body bends, defined as a complete left–right movement of the body, over a 20 second interval. Ten worms per strain were analyzed, and each worm was scored twice. The mean number of body bends per worm was used for subsequent analysis.

Video-based locomotion analysis: Spontaneous locomotion of age-synchronized day-8 adult worms was recorded using the WMicrotracker® SMART-8x Carousel system (Phylumtech) on unseeded 35 mm NGM plates. Worms were monitored for 5 minutes under standardized conditions. Locomotion parameters, including movement speed (mm s^−1^), were extracted and exported to Excel for quantitative analysis.

## Bioethical statement

All experiments were performed in accordance with the guidelines of the Declaration of Helsinki (1989) of the World Medical Association, and experiments were approved by the ethics committee at Harokopio University. Informed consents were obtained from human participants of this study.

## Conflicts of interest

There is no conflict of interest to declare.

## Supplementary Material

MD-017-D6MD00265J-s001

## Data Availability

Data are available upon request from the authors. Supplementary information (SI): the SI contains the complete 1D and 2D NMR spectra, MS spectra, and QSAR data tables associated with this study. See DOI: https://doi.org/10.1039/d6md00265j
