# Supplementary material for: Novel hydroxytyrosol esters as potential anti-amyloid and neuroprotective agents for Alzheimer's disease
Source: RSC Med Chem. 2026 Jun 4;17(7):3297–312. doi: 10.1039/d6md00265j (PMC13267185; doi:10.1039/d6md00265j)
Supplement: MD-017-D6MD00265J-s001 [file MD-017-D6MD00265J-s001.pdf]

Supplementary Information (SI) for RSC Medicinal Chemistry.
This journal is © The Royal Society of Chemistry 2026

**Supplementary Information**

# Novel Hydroxytyrosol Esters as Potential Anti-Amyloid and Neuroprotective Agents for Alzheimer's Disease

Ioanna Kalpaktsi[1], Anthi Panara[2], Barbara Mavroidi[3], Giorgos Garcia Niforos[4], Amalia D. Kalampaliki[1], Ioanna C. Vlachogianni[5], Eleftheria A. Georgiou,[1] Elizabeth Fragopoulou[5], Anthony Tsarbopoulos[6], Alexios-Leandros Skaltsounis[7], Maria Pelecanou[8], Kontantinos Palikaras[4], Evangelos Gikas[2], Ioannis K. Kostakis[1,*]

[1]Division of Pharmaceutical Chemistry, Department of Pharmacy, National and Kapodistrian University of Athens, Panepistimiopolis Zografou 15771, Athens, Greece
[2]Laboratory of Analytical Chemistry, Department of Chemistry, National and Kapodistrian University of Athens, Panepistimiopolis, Zografou, Athens 15771, Greece.
[3]Institute of Biosciences & Applications, National Centre for Scientific Research "Demokritos", 15310 Athens, Greece
[4]Department of Physiology, Medical School, National and Kapodistrian University of Athens, Athens 11527, Athens, Greece
[5]Department of Nutrition & Dietetics, School of Health Sciences and Education, Harokopio University, Athens, Greece
[6]Department of Pharmacology, Medical School, National and Kapodistrian University of Athens, 11527 Athens, Greece
[7]Division of Pharmacognosy and Natural Products Chemistry, Department of Pharmacy, National and Kapodistrian University of Athens, Panepistimiopolis Zografou 15771, Athens, Greece

* Corresponding author.

E-mail address: ikkostakis@pharm.uoa.gr (IKK).

# Contents

**NMR Spectra** .......... 3

**Figures** .......... 31

**Tables** .......... 35

# NMR Spectra

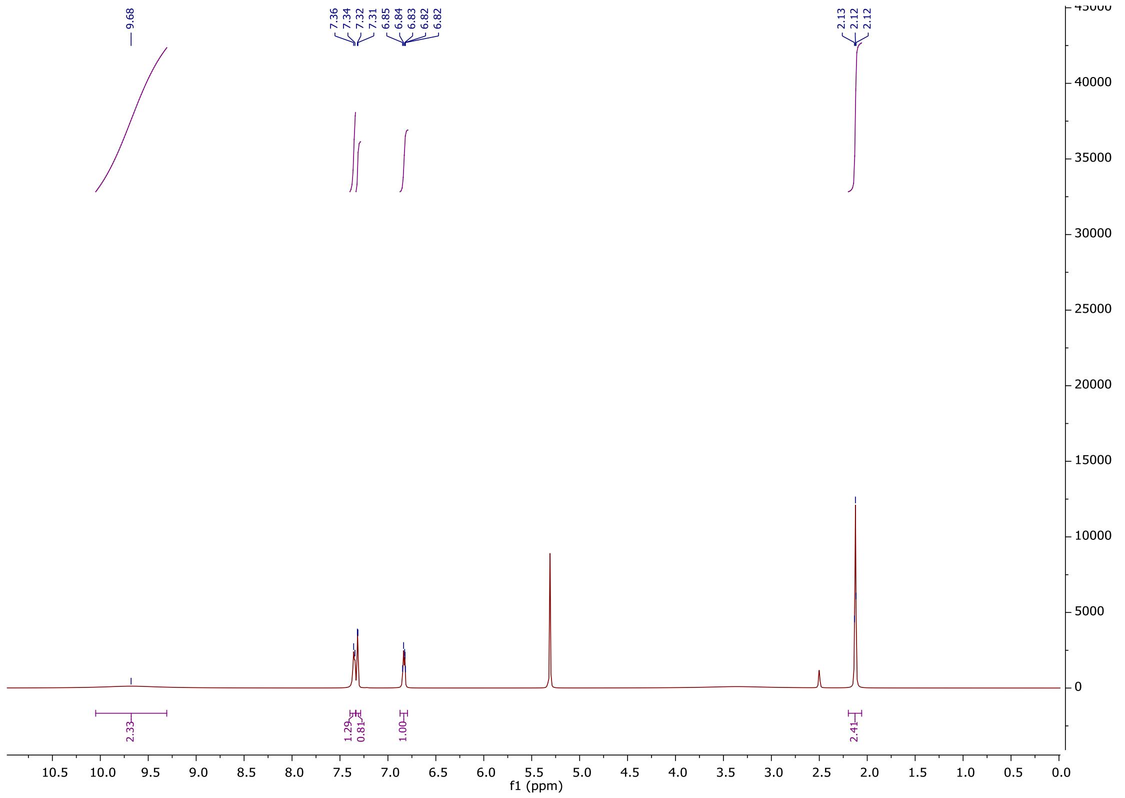


$^{1}$H NMR spectrum of **3a.**

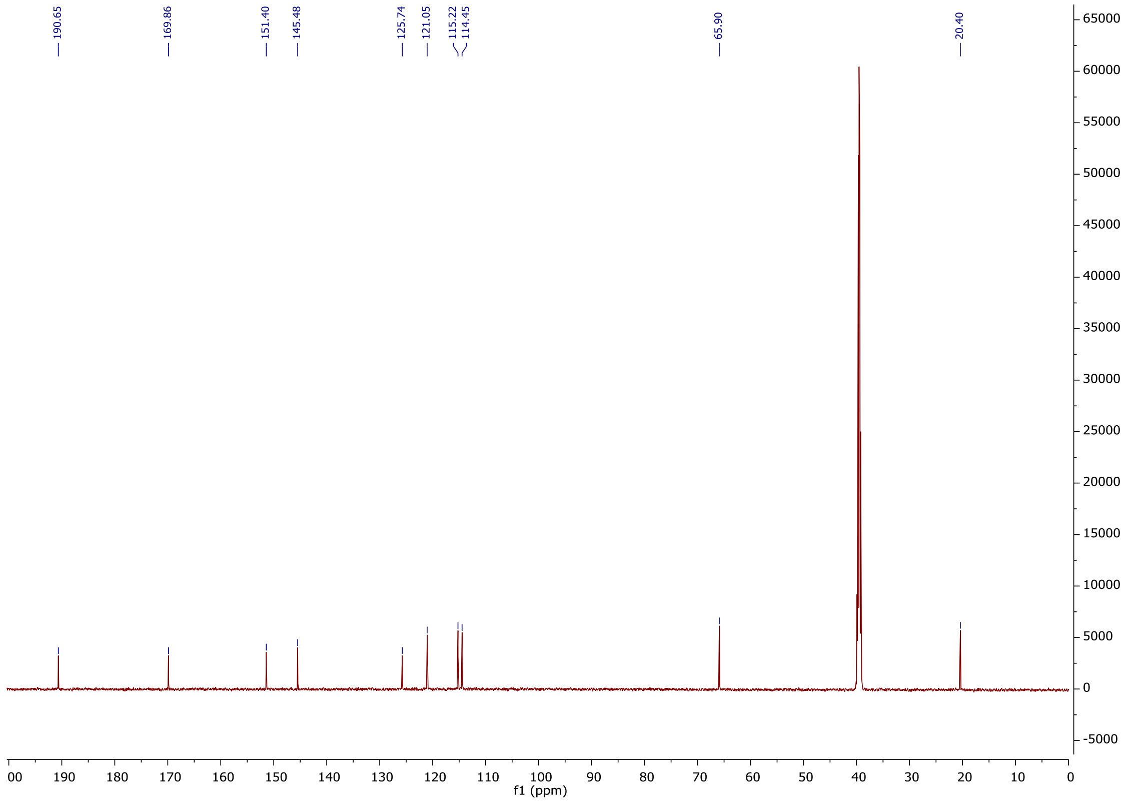


$^{13}$C NMR spectrum of **3a.**

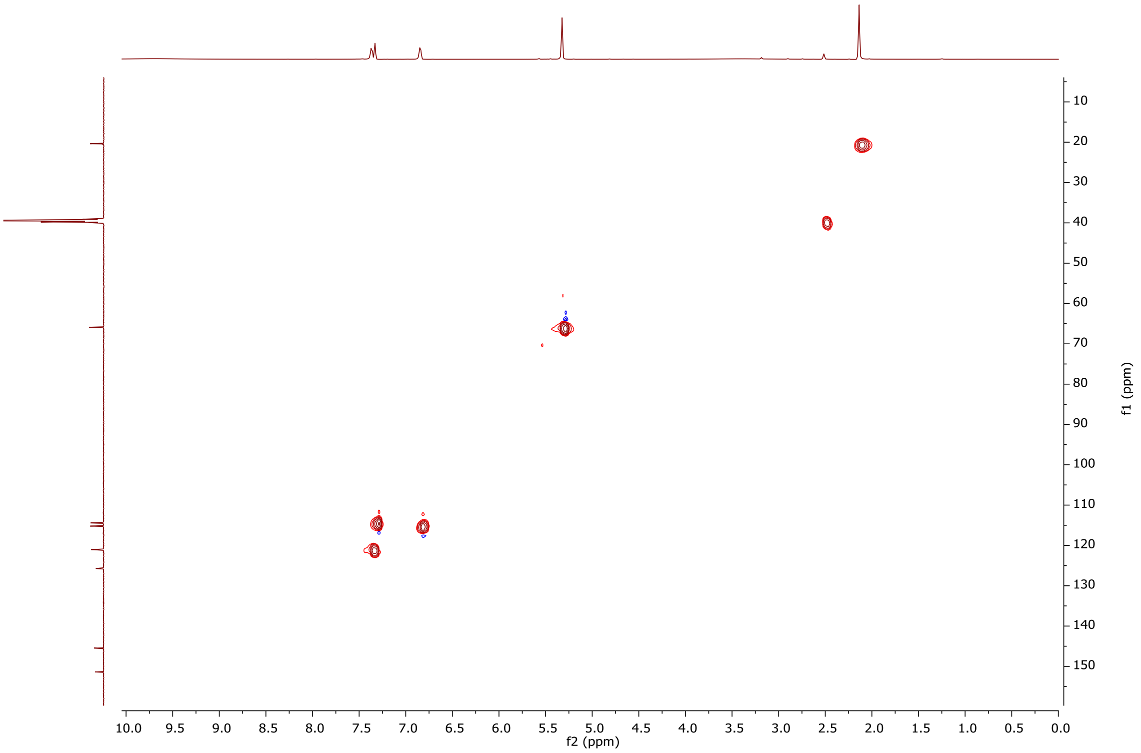


HSQC spectrum of **3a.**

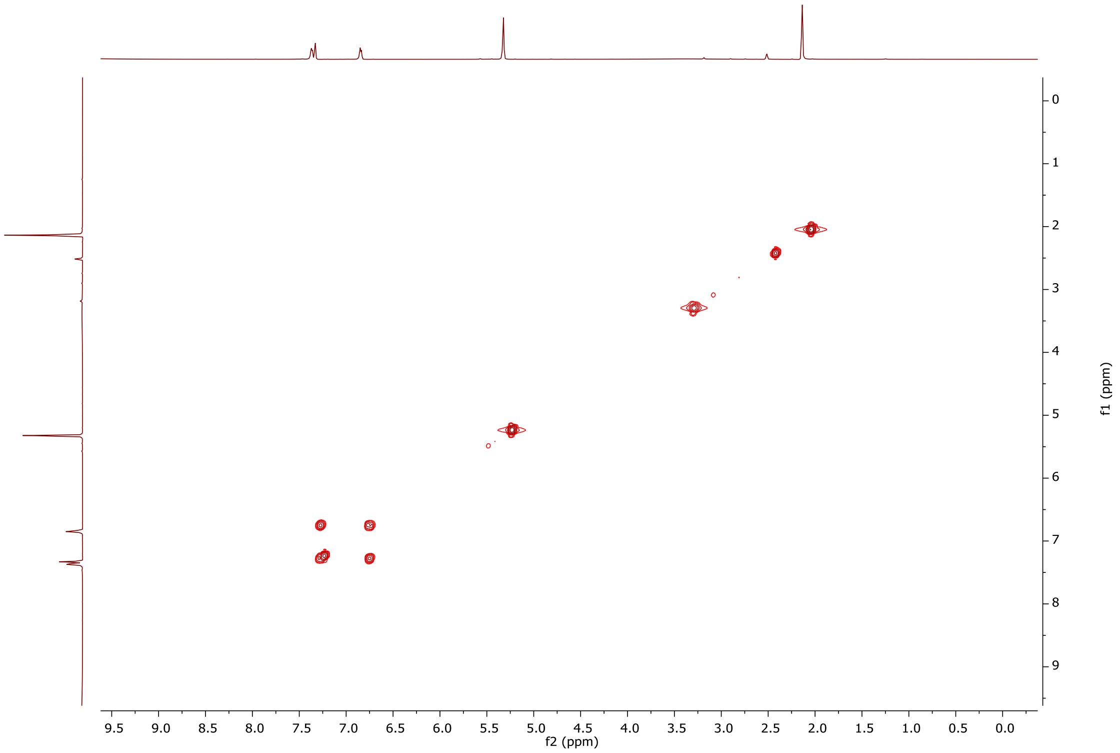


COSY spectrum of **3a.**

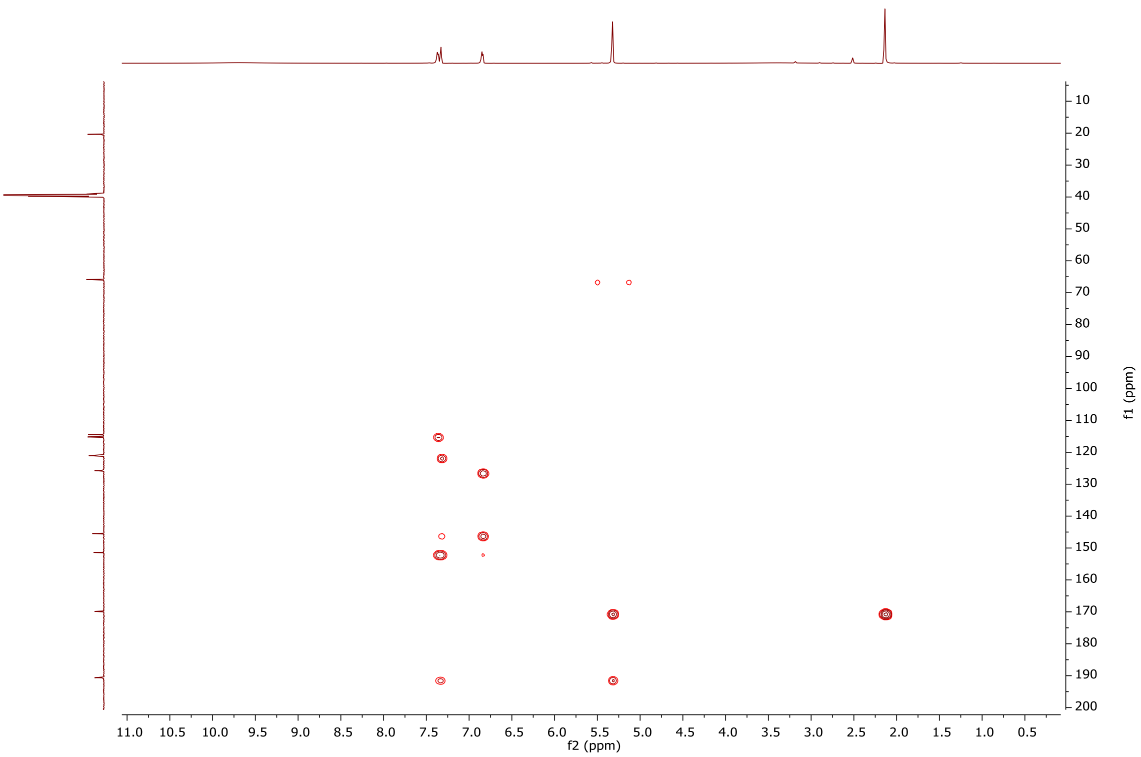


HMBC spectrum of **3a.**

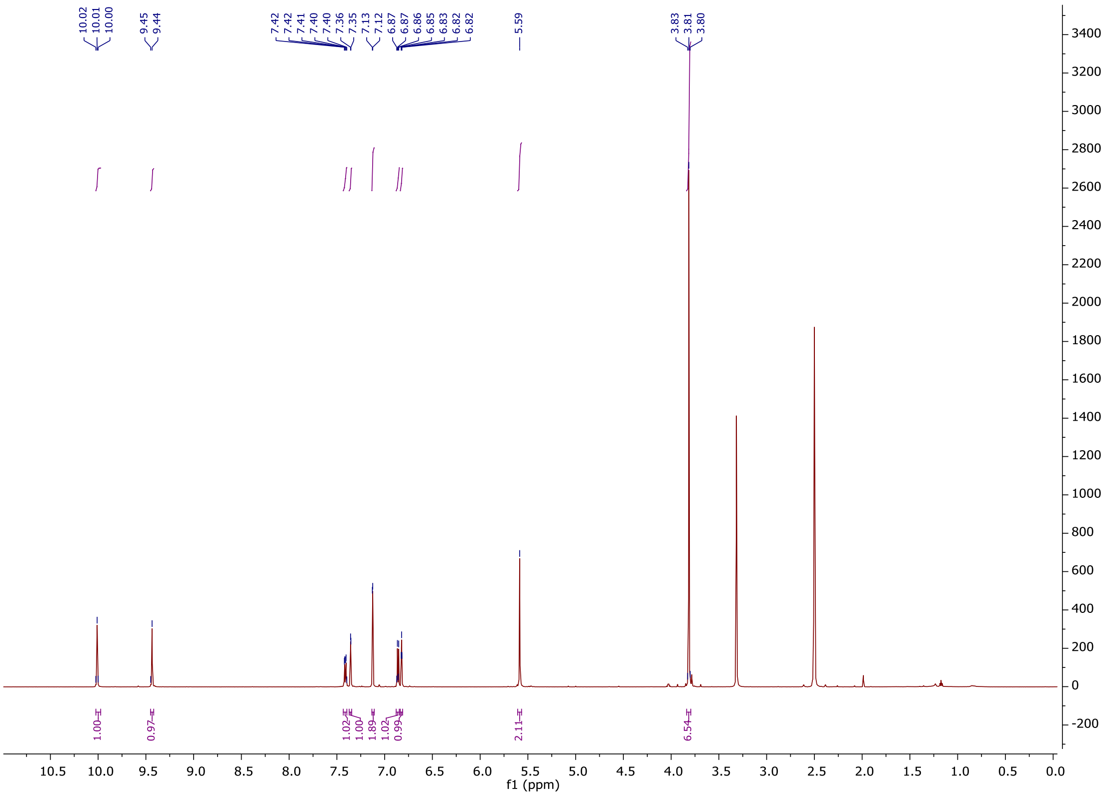


$^1H$ $^1H$ NMR spectrum of **3b.**

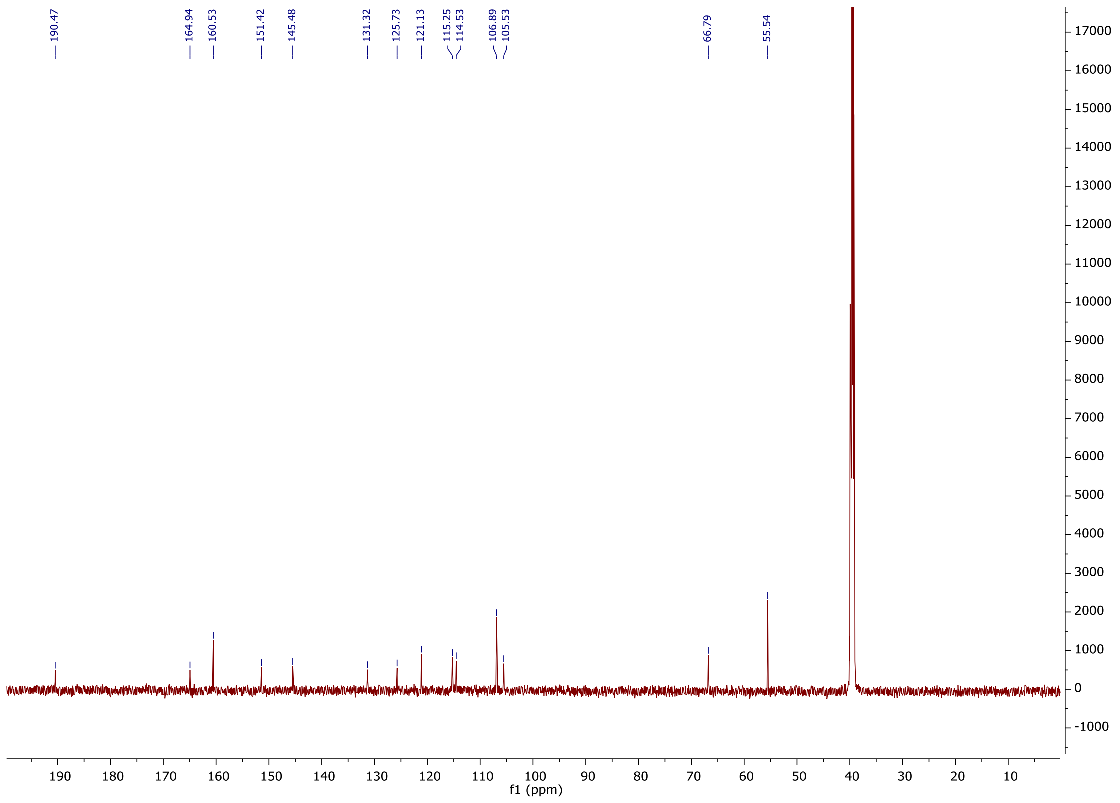


$^{13}$C NMR spectrum of **3b0**

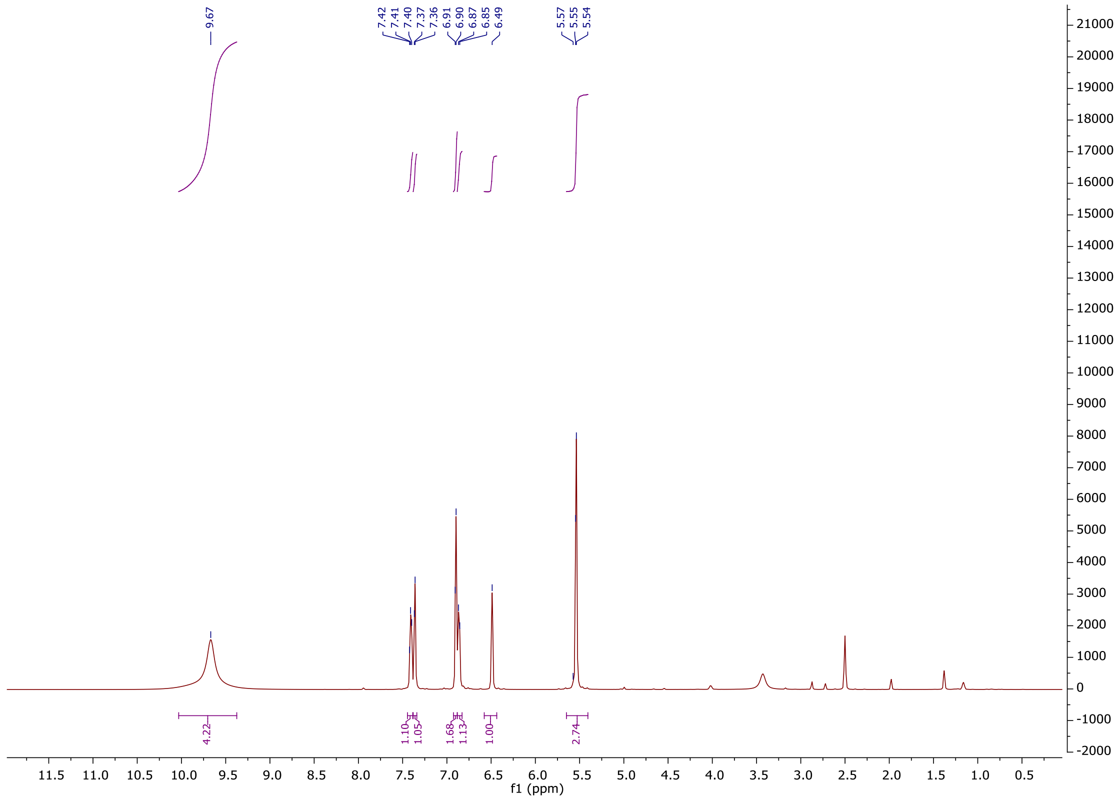


$^{1}$H NMR spectrum of **3c**

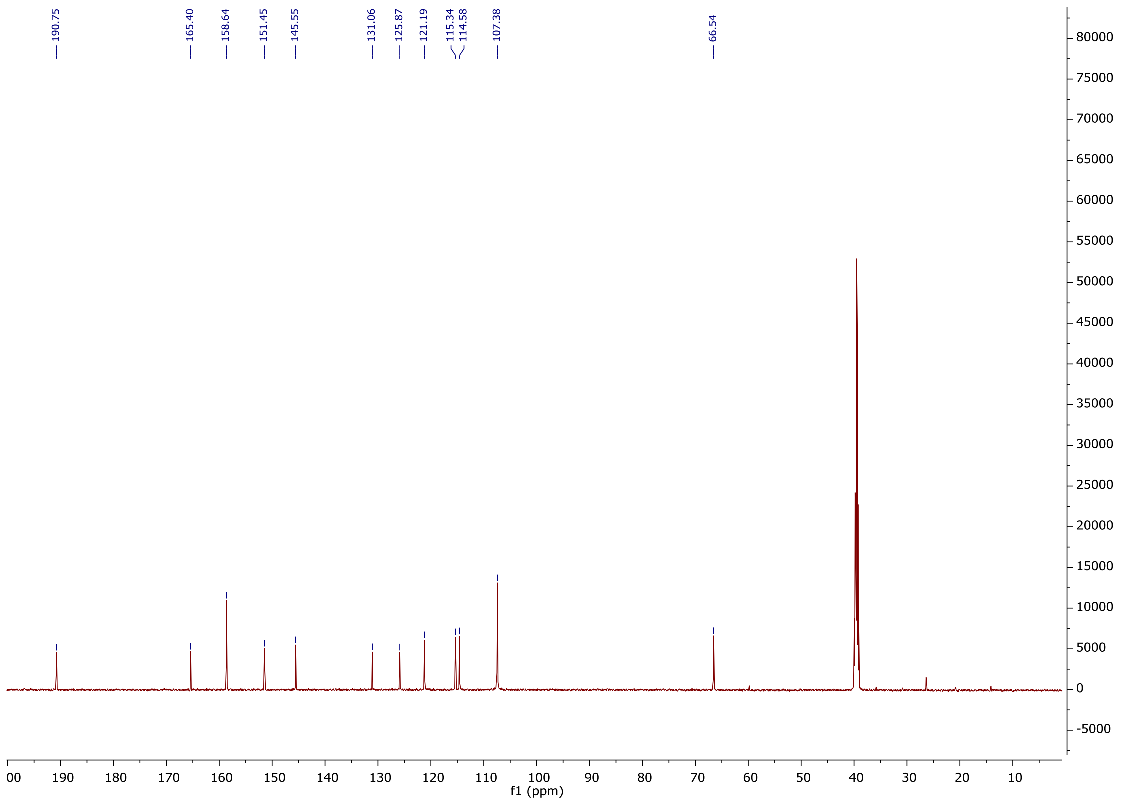


$^{13}$C NMR spectrum of **3c**.

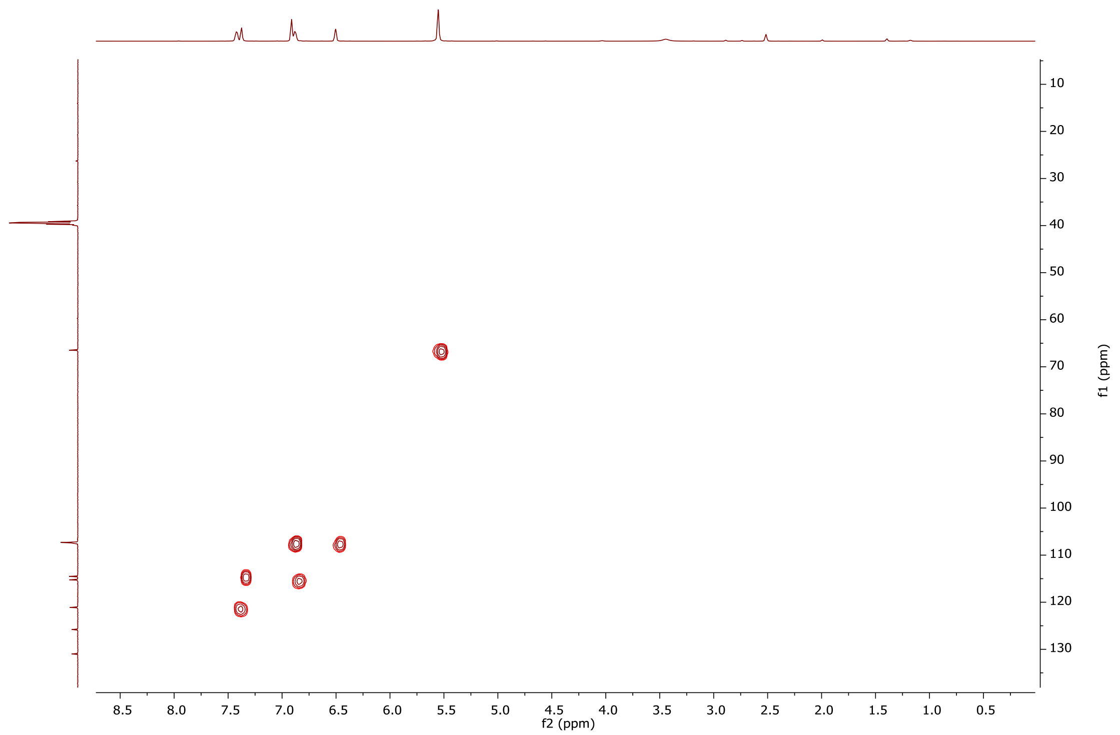


HSQC spectrum of **3c.**

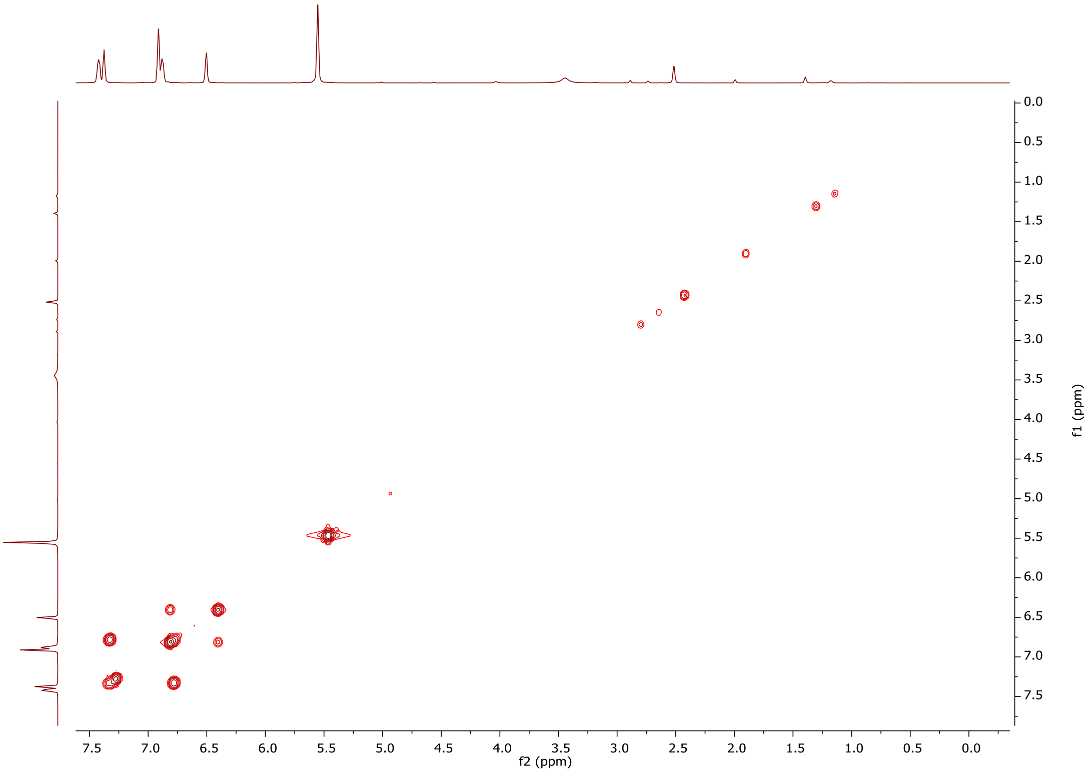


COSY spectrum of **3c.**

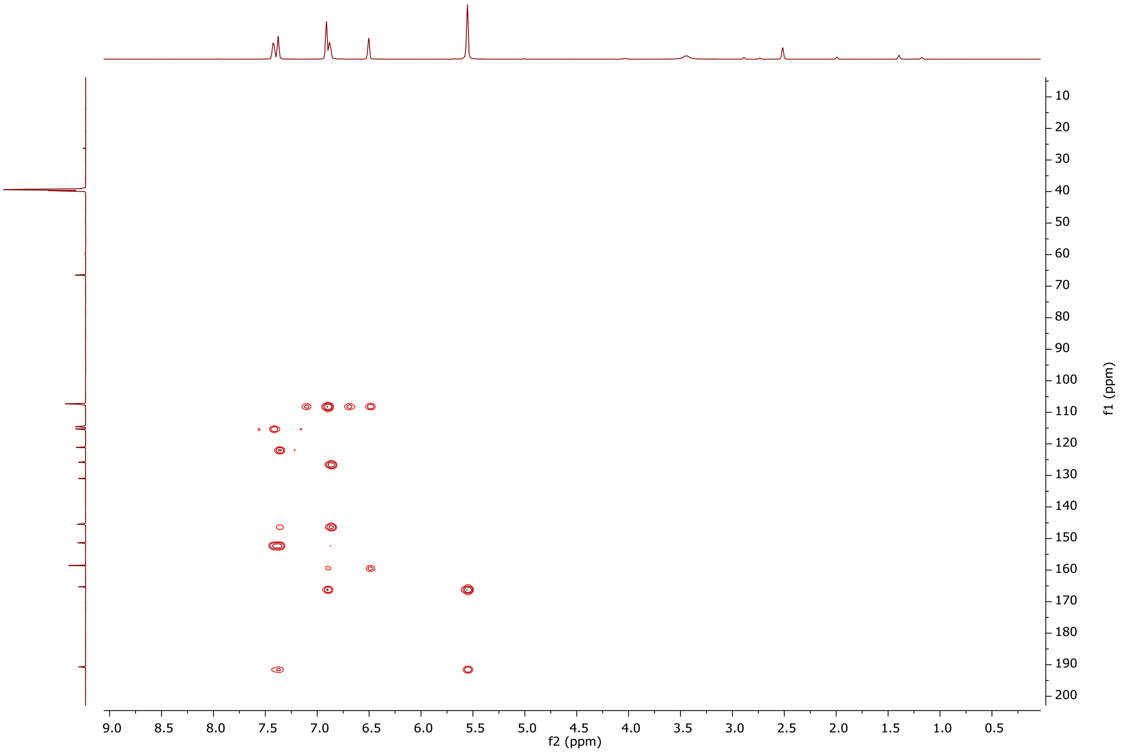


HMBC spectrum of **3c.**

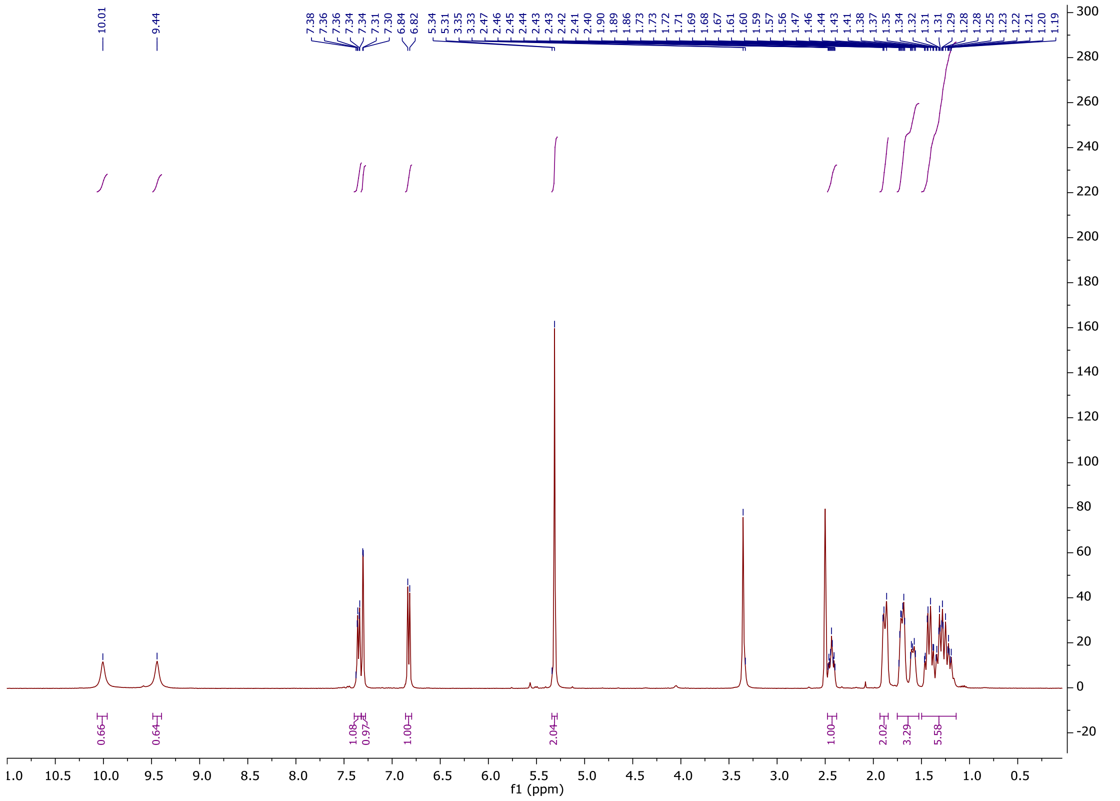


$^{1}$H NMR spectrum of **3d**

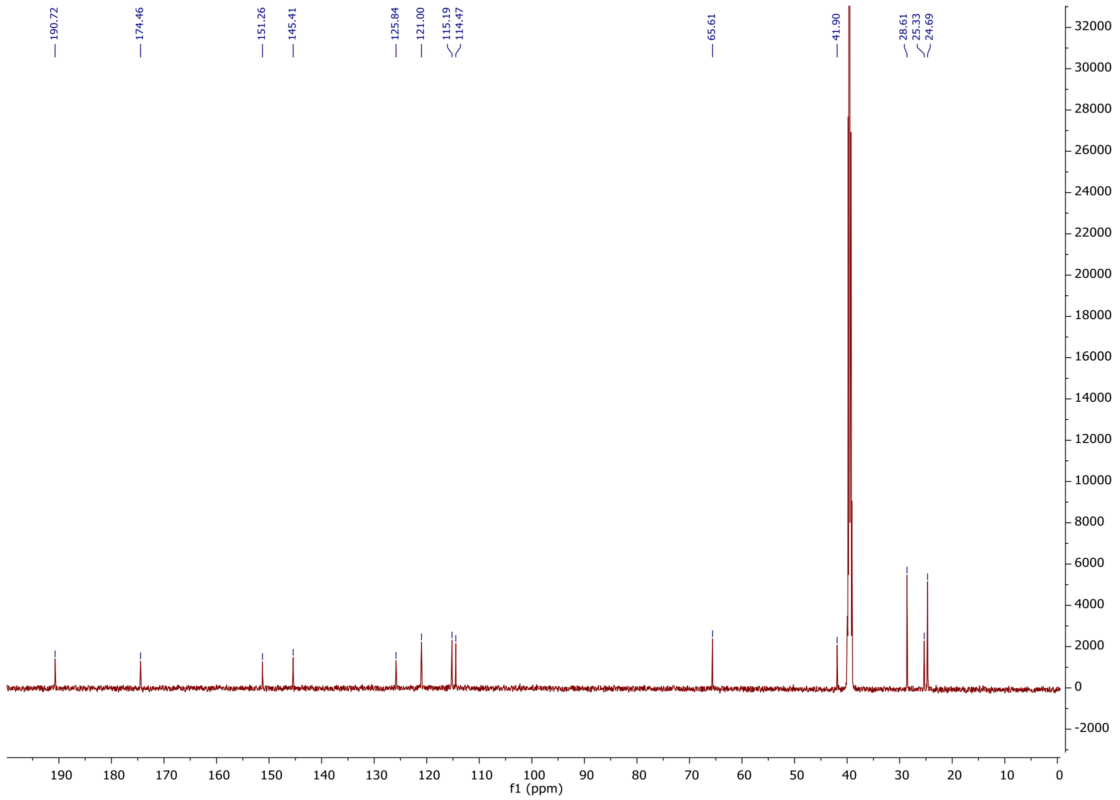


$^{13}$C NMR spectrum of **3d**

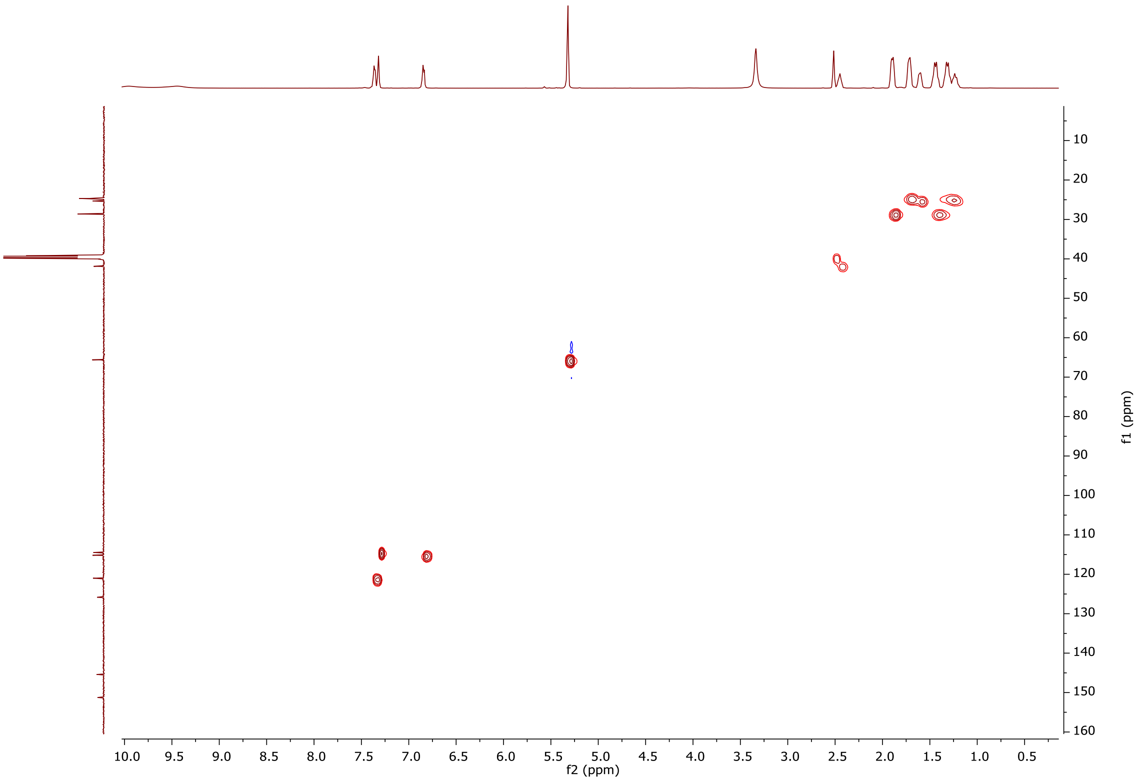


HSQC spectrum of **3d**

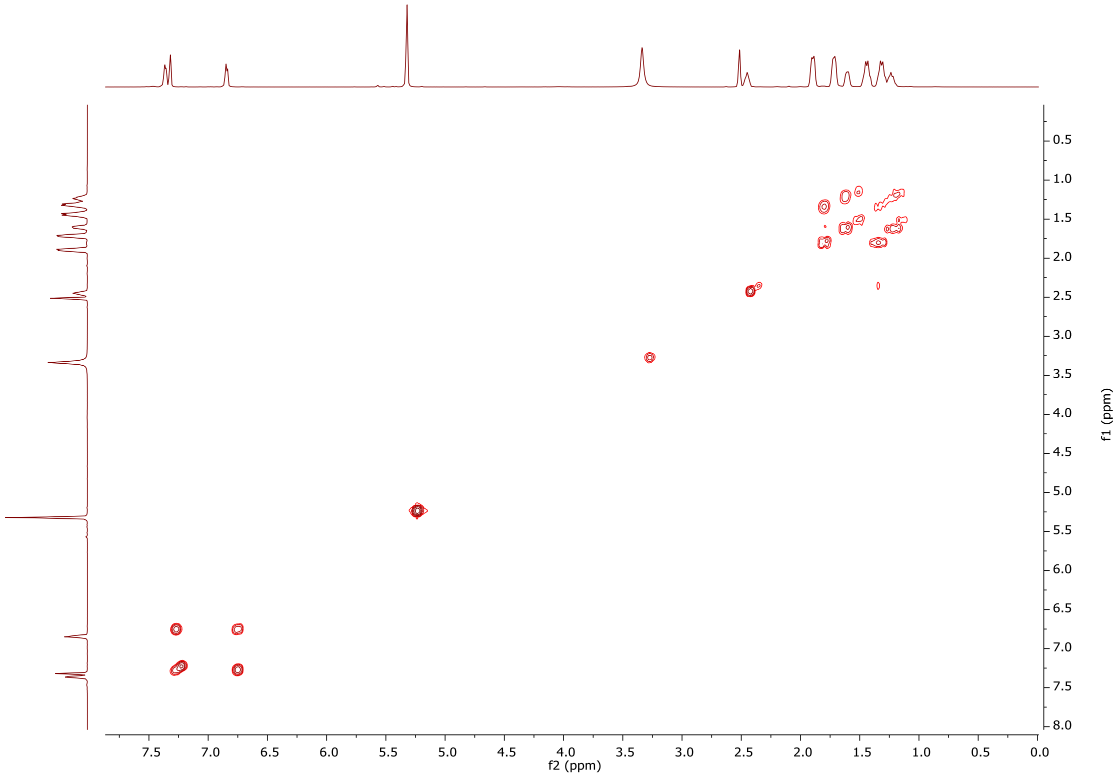


COSY spectrum of **3d**

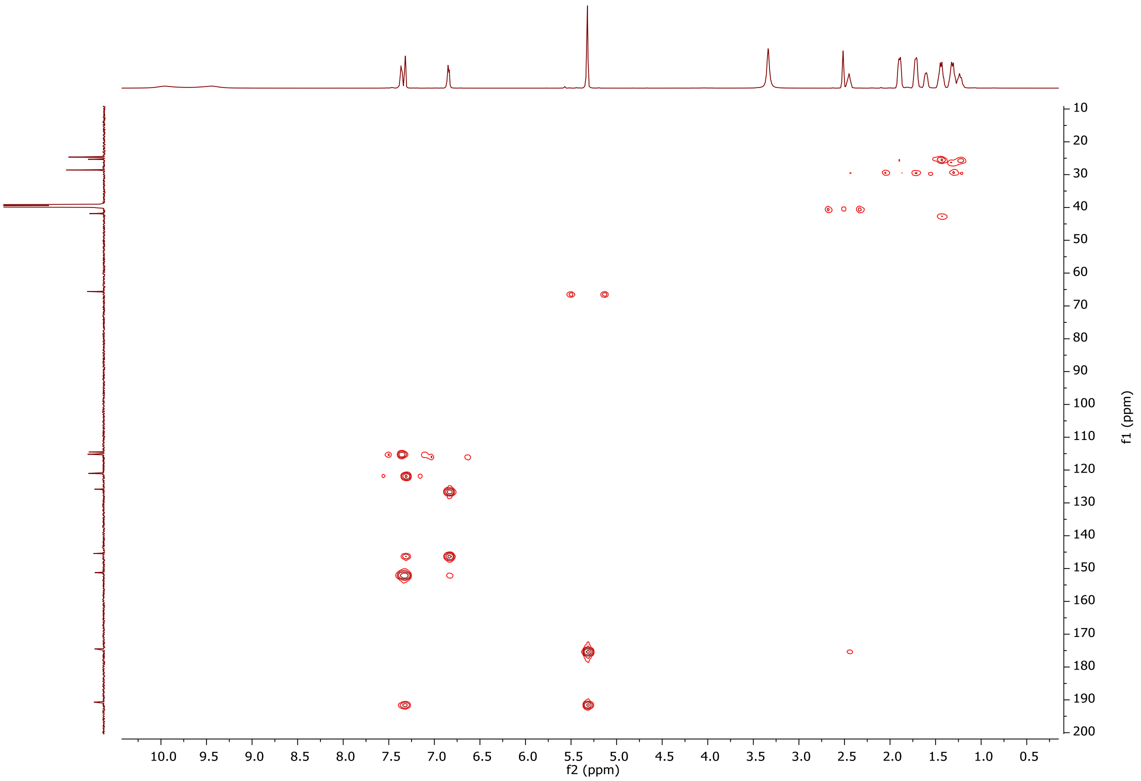

HMBC spectrum of **3d**

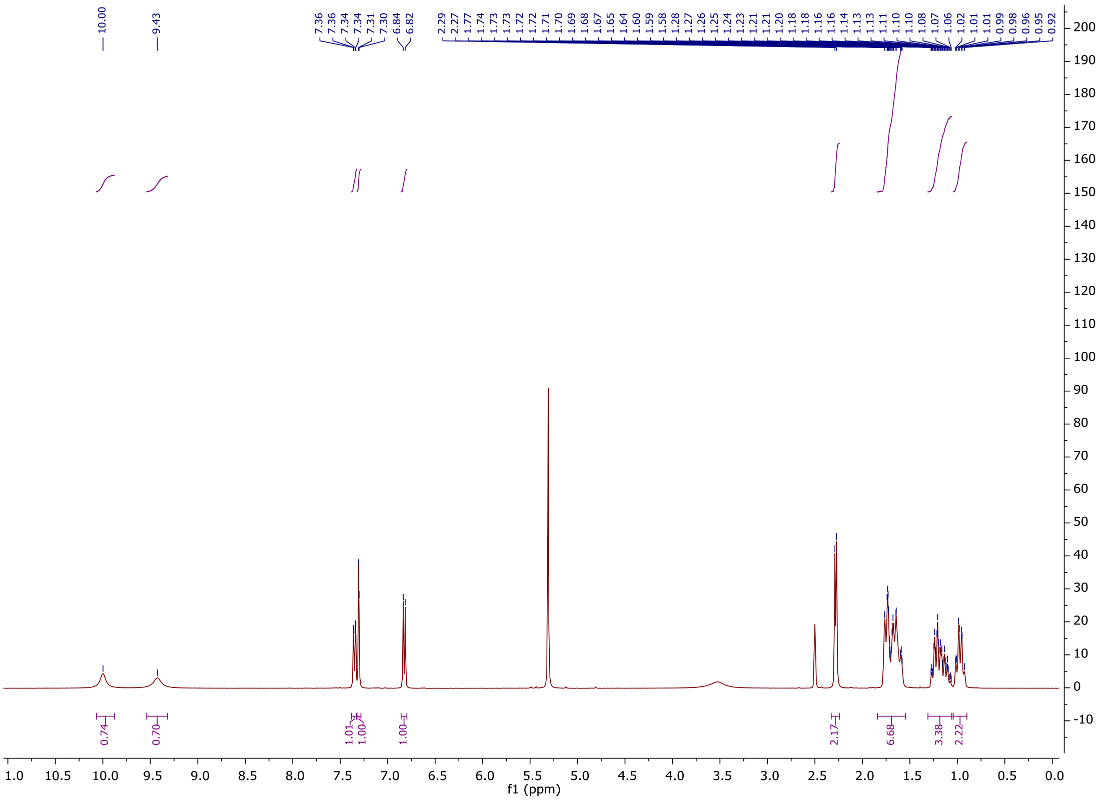

$^{1}$H NMR spectrum of **3e**

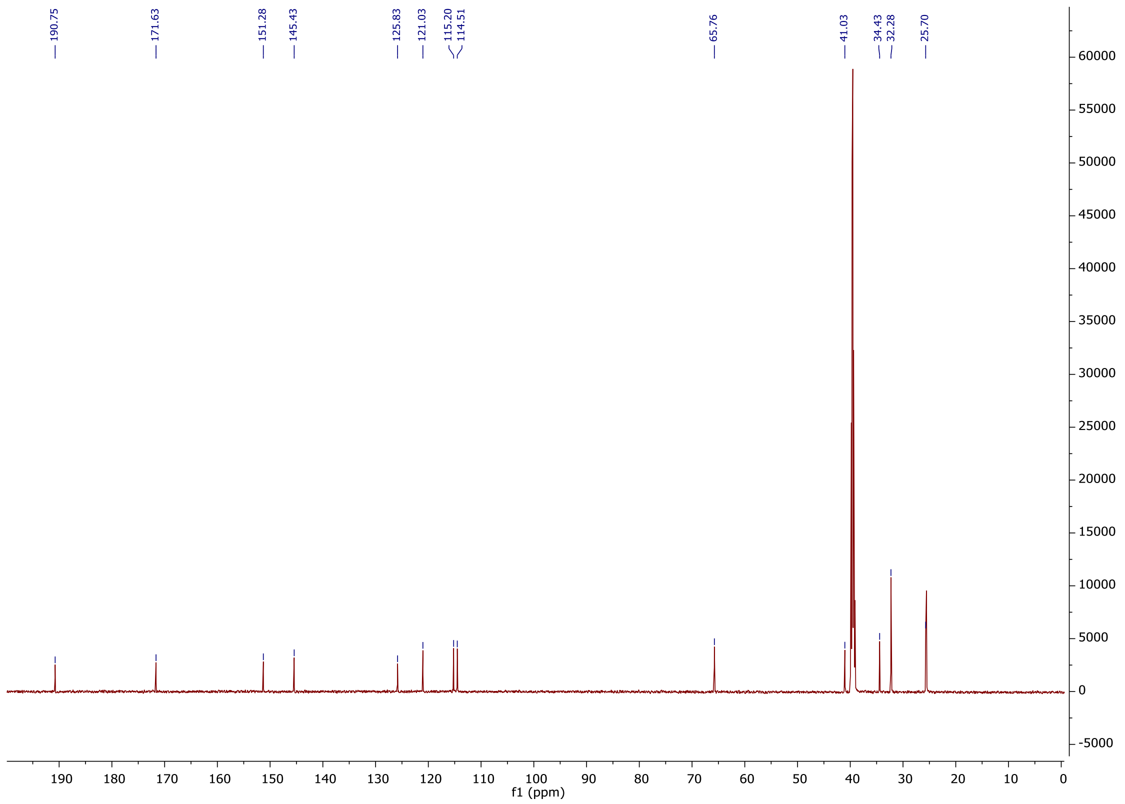


$^{13}$C NMR spectrum of **3e**

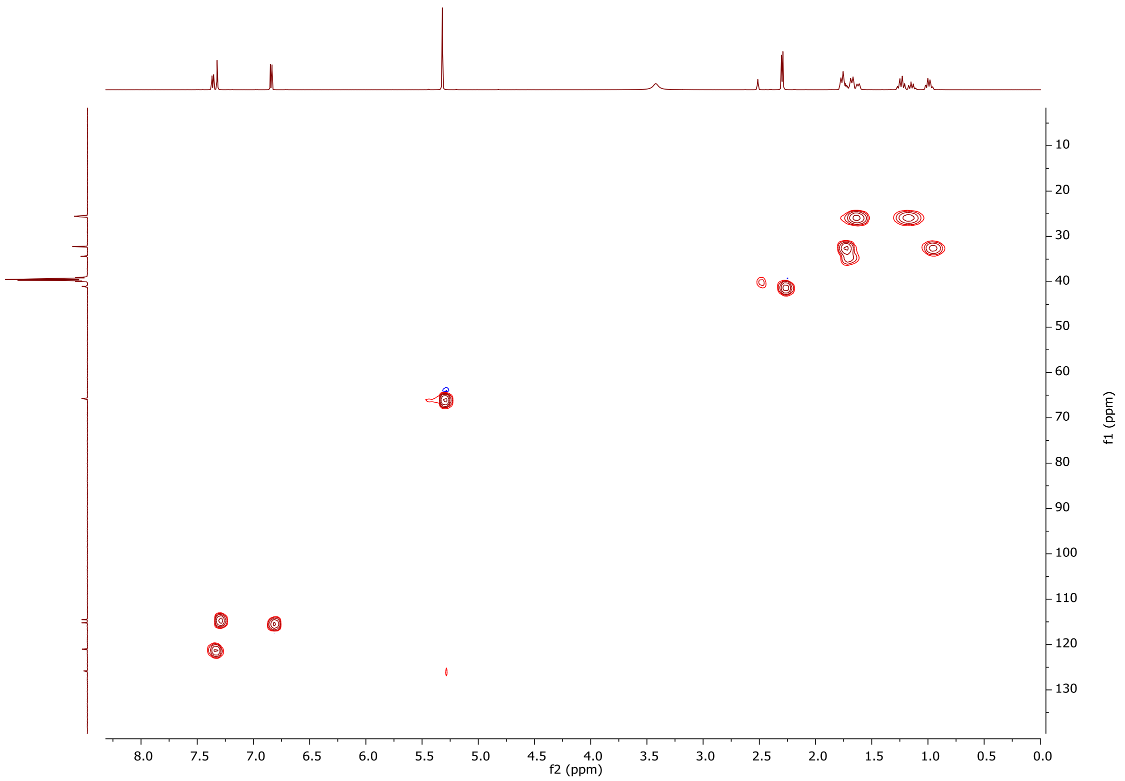


HSQC spectrum of **3e**

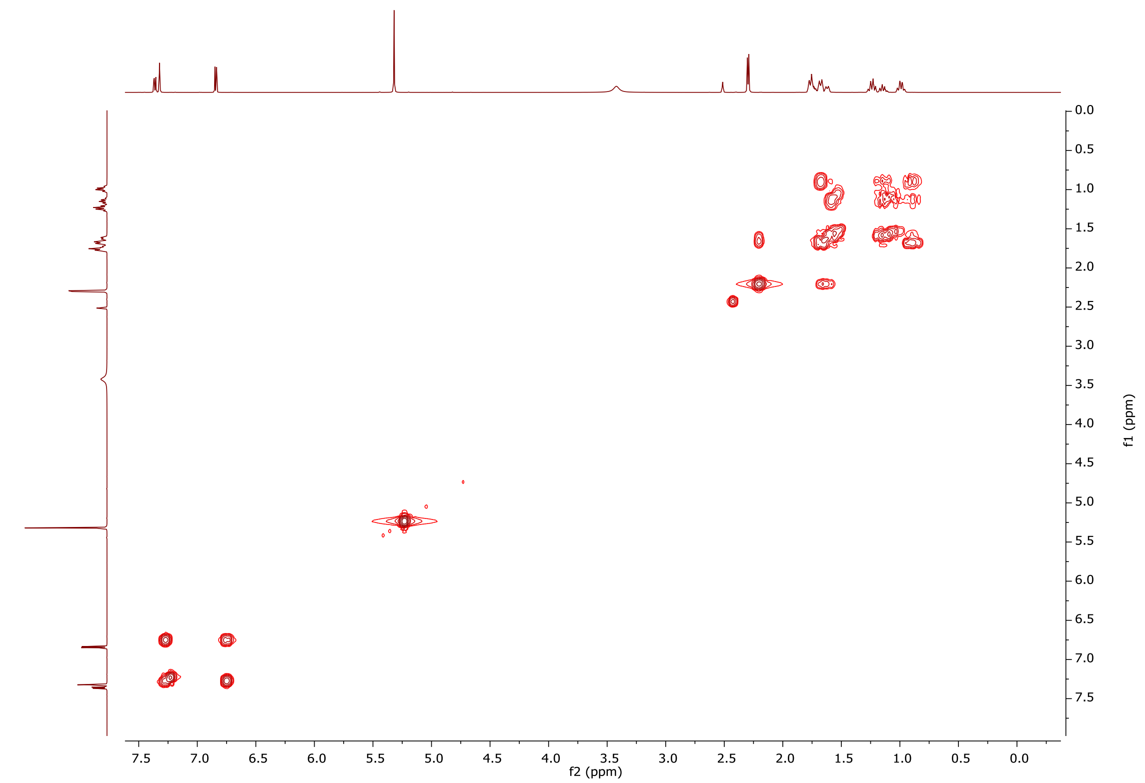


COSY spectrum of **3e**

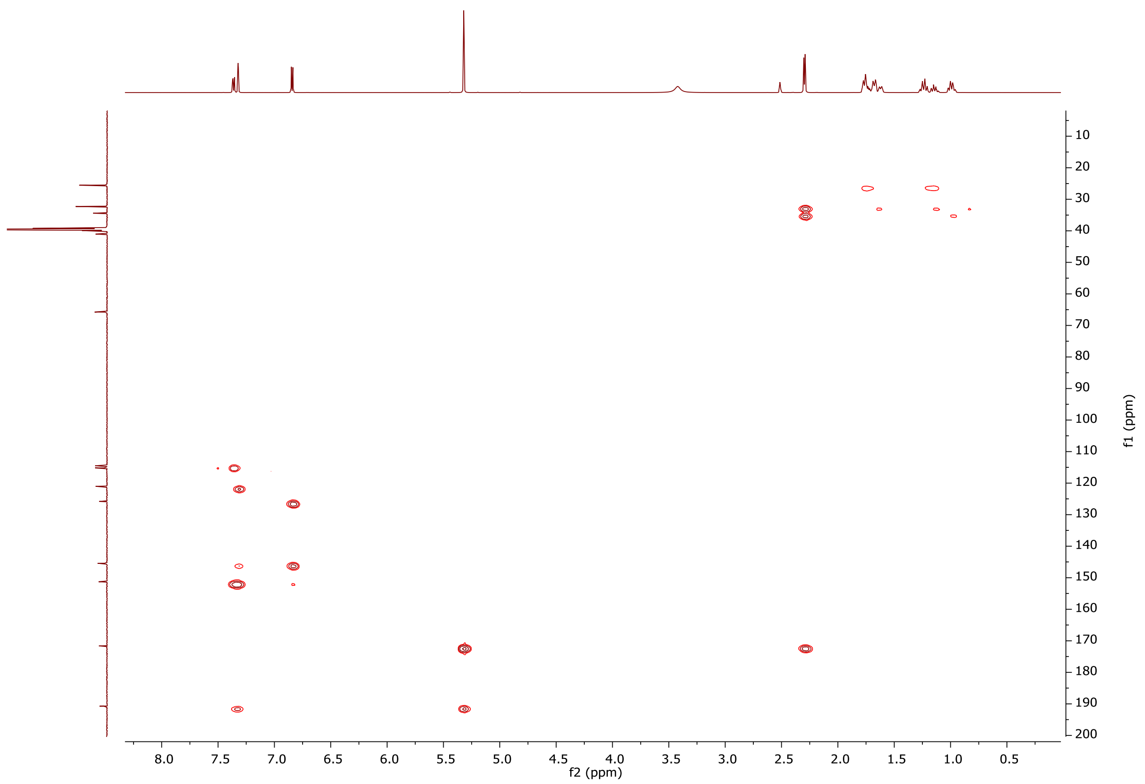


HMBC spectrum of **3e**

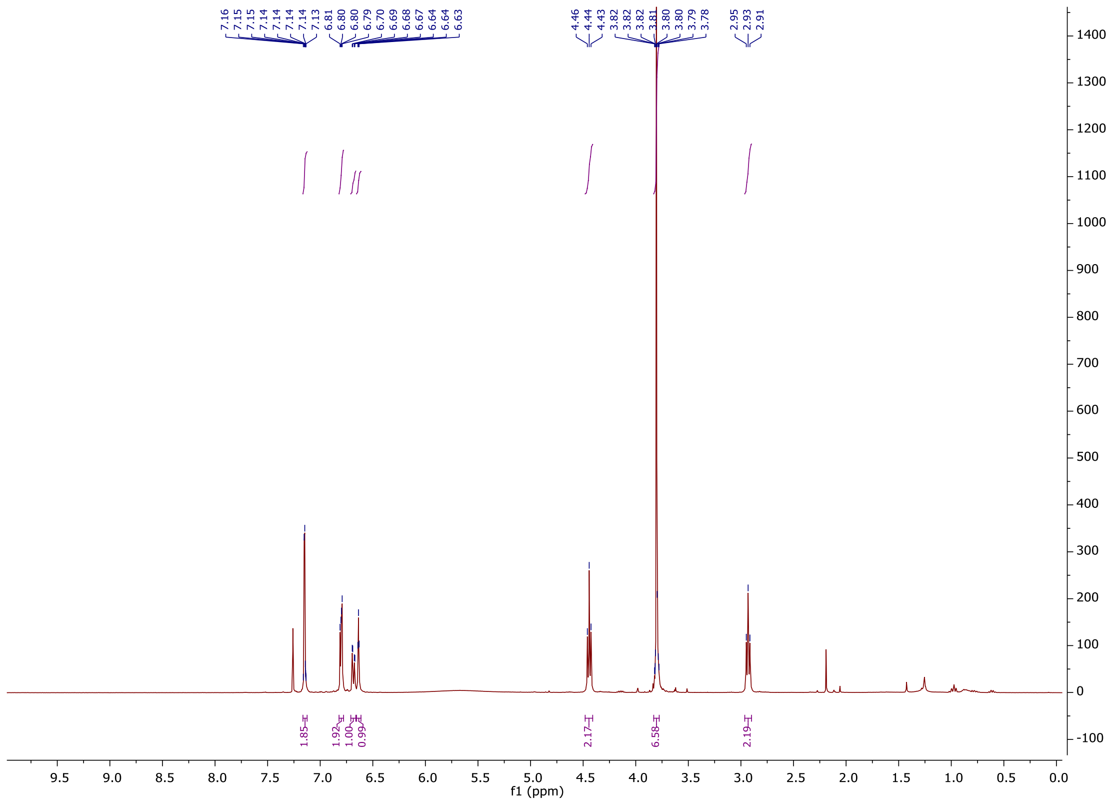


$^{1}$H NMR spectrum of **4b**.

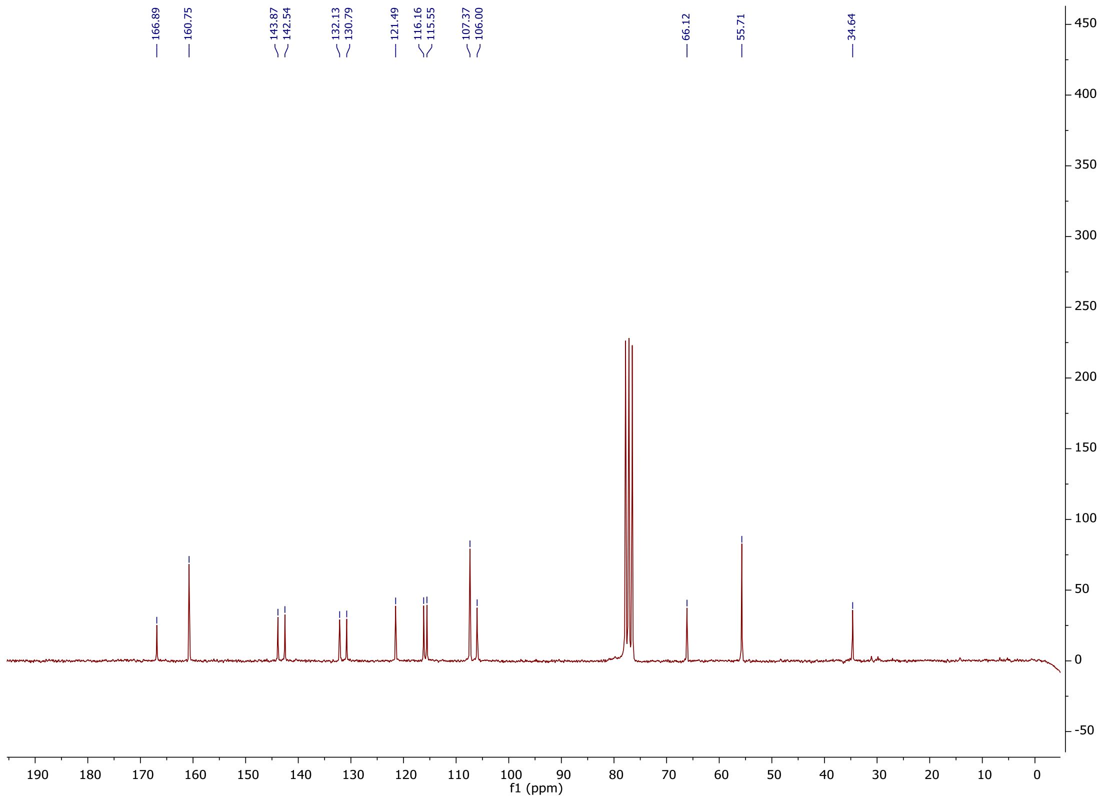


$^{13}$C NMR spectrum of **4b**

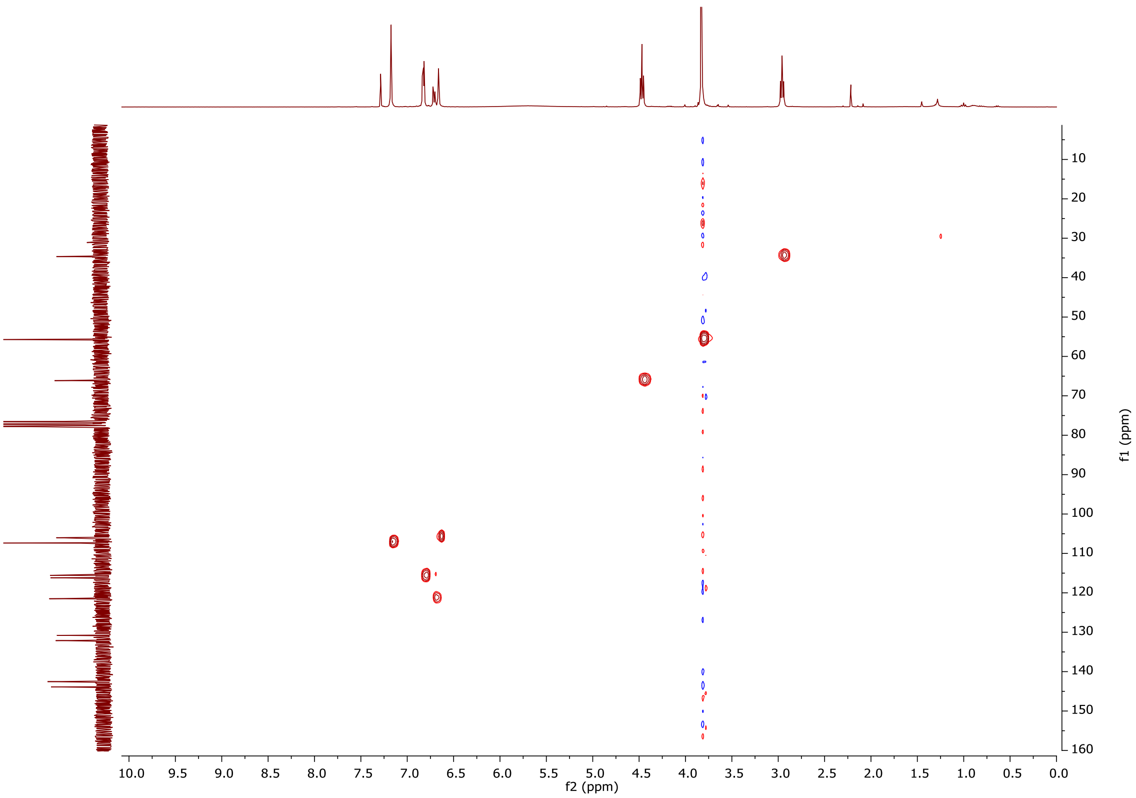


HSQC spectrum of **4b**.

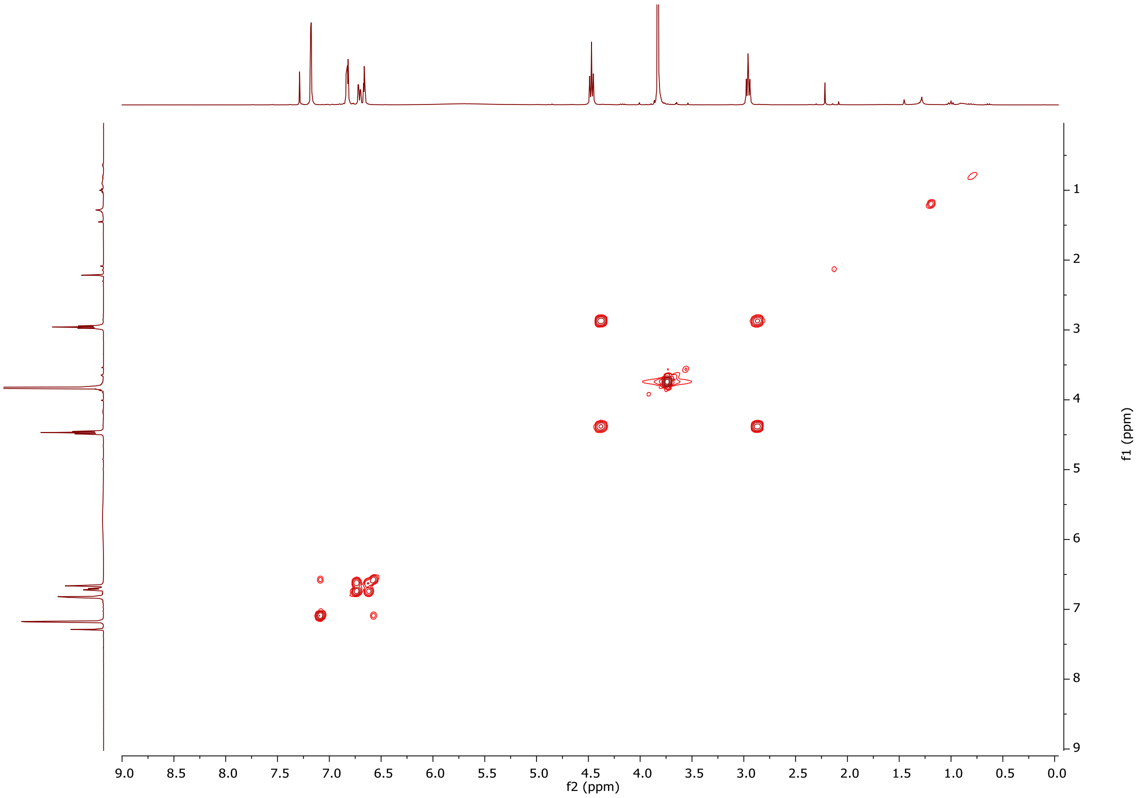


HSQC spectrum of **4b**.

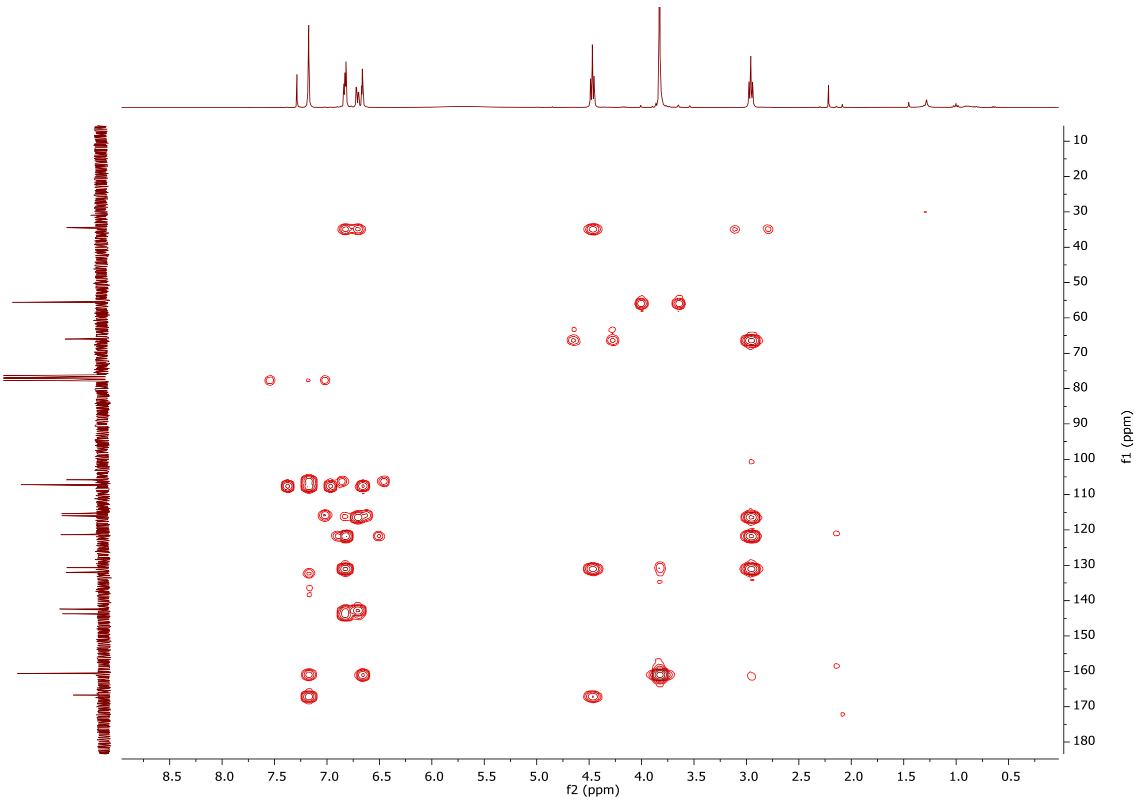


HMBC spectrum of **4b**.

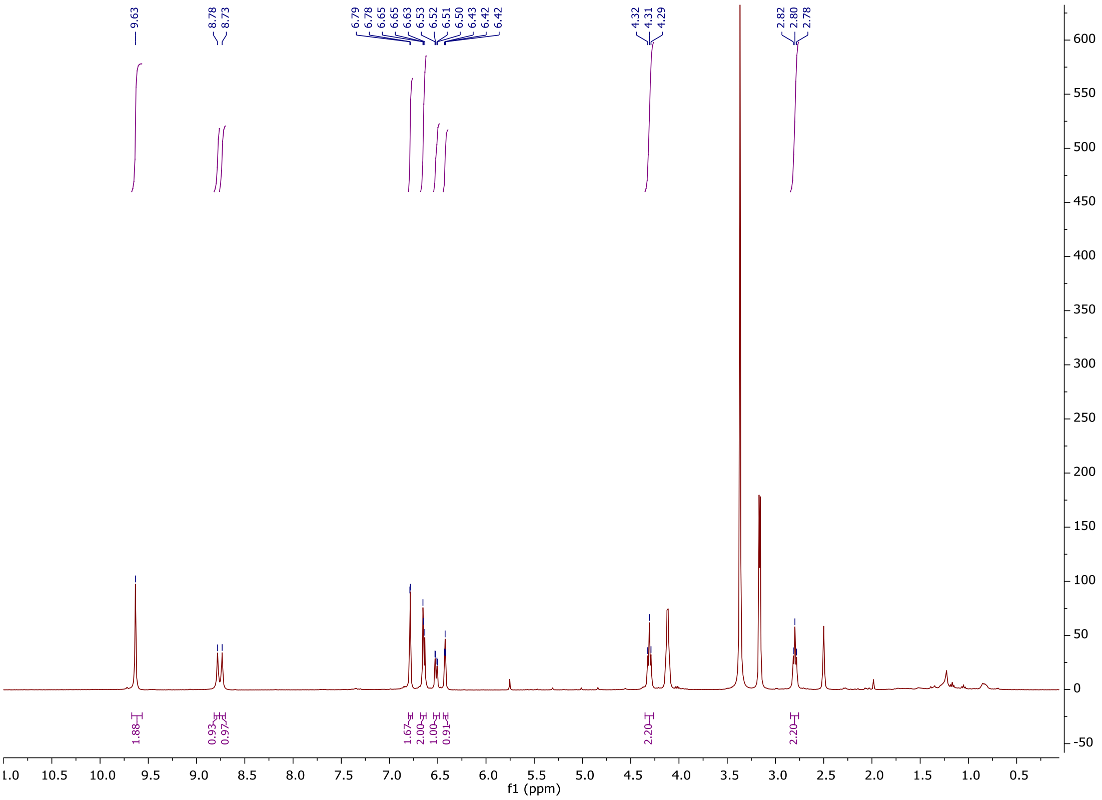


$^{1}$H NMR spectrum of **4c**.

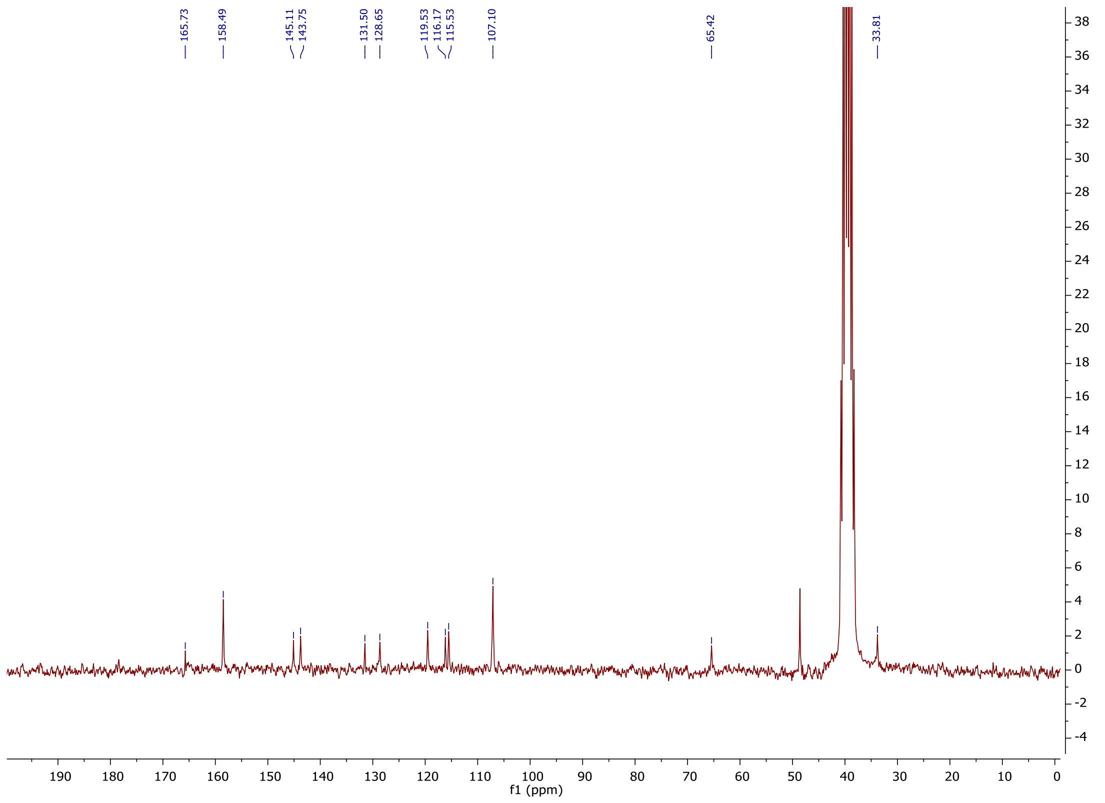


$^{13}$C NMR spectrum of **4c**

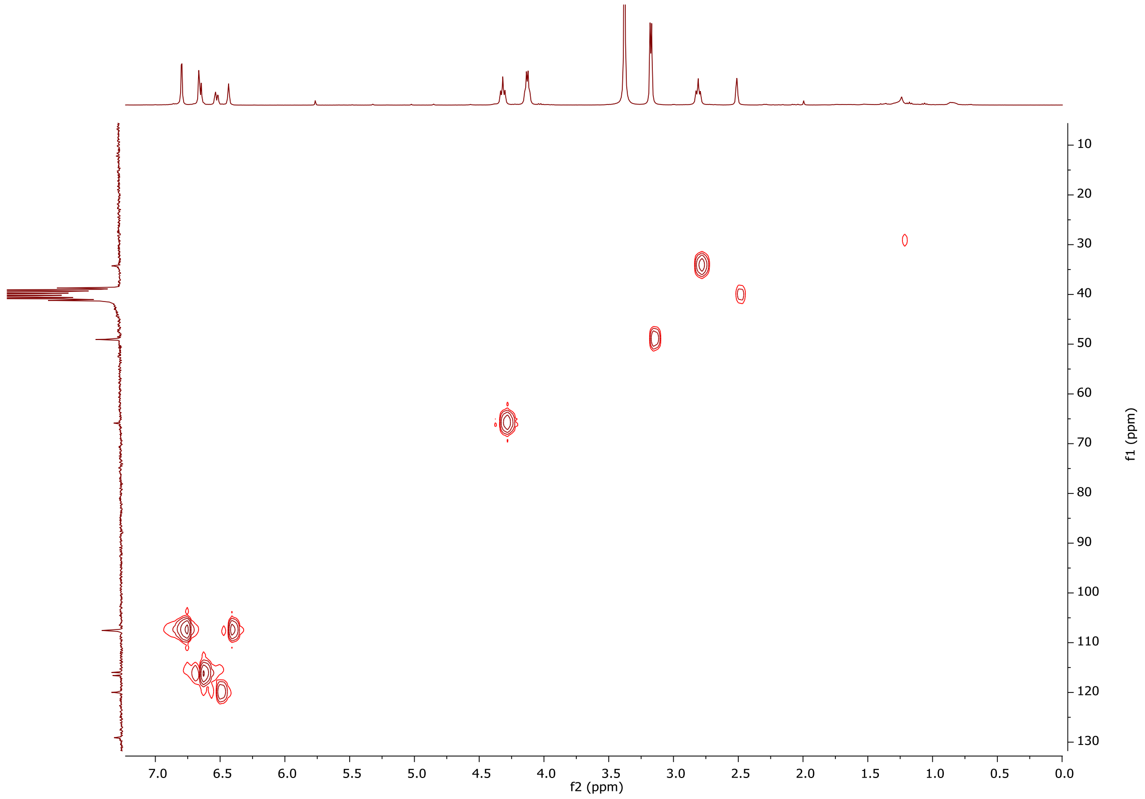


HSQC spectrum of **4c**

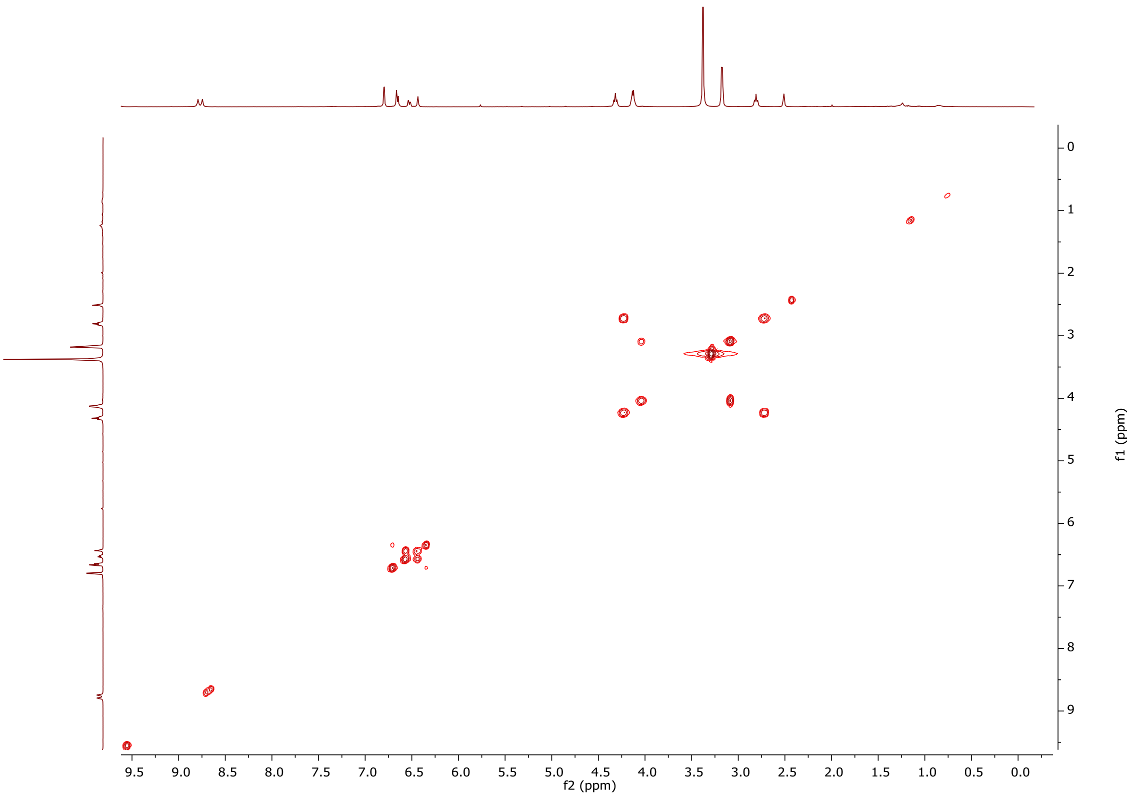


COSY spectrum of **4c**

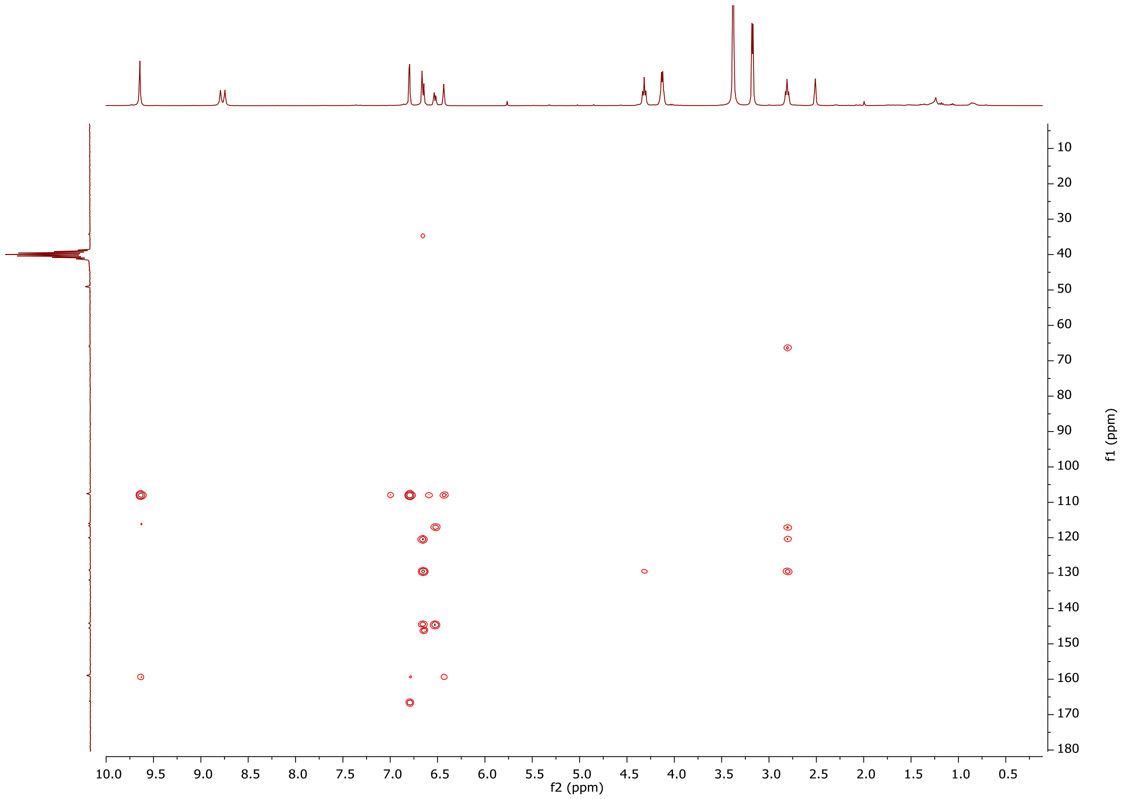


HMBC spectrum of **4c**

.

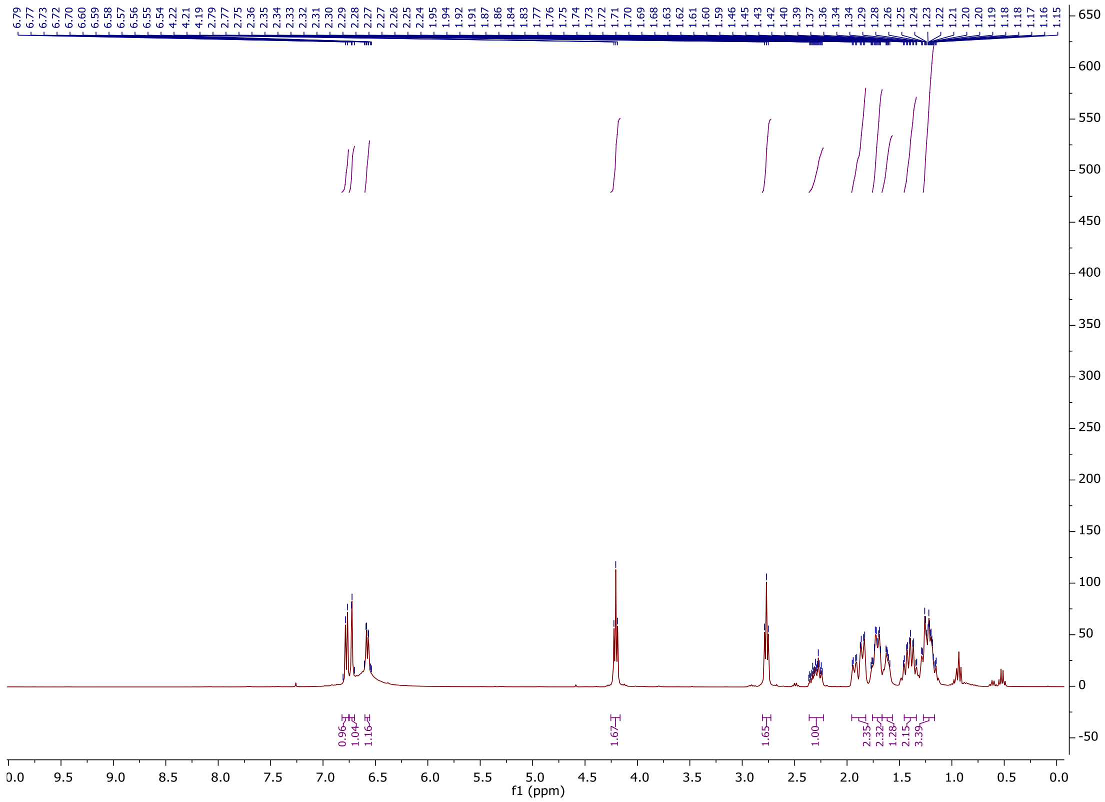

$^{1}$H NMR spectrum of **4d**.

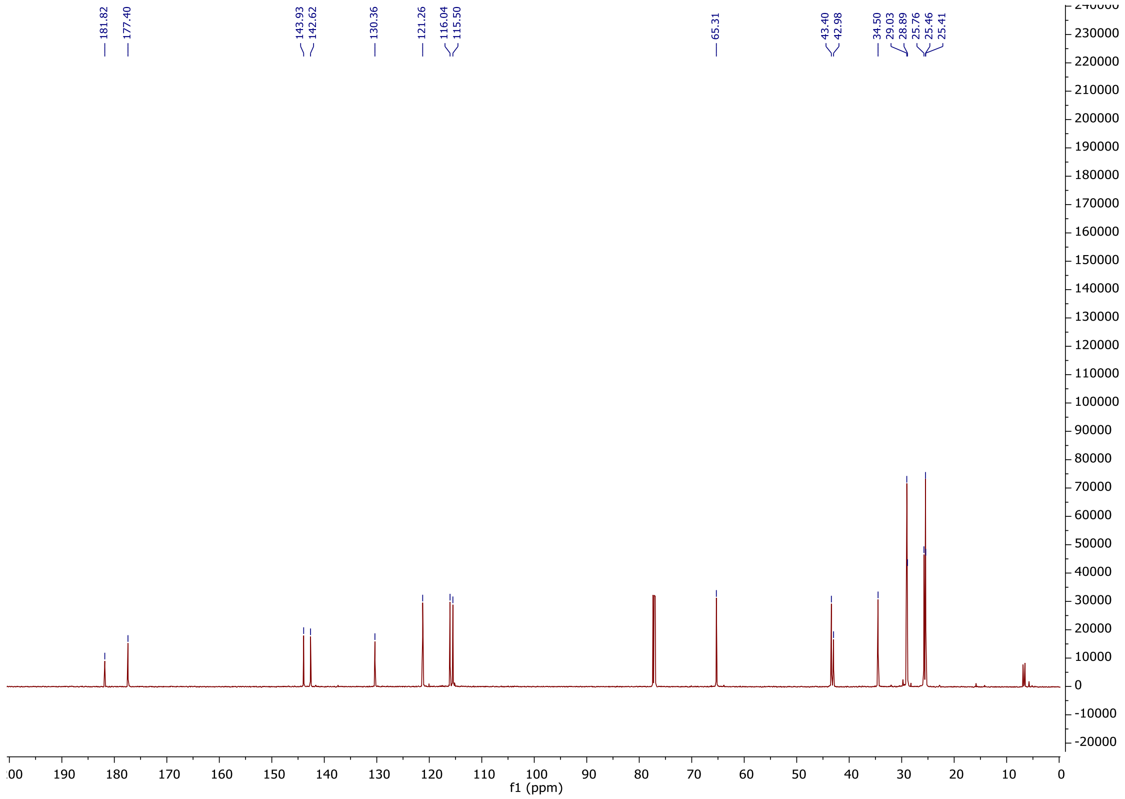

$^{13}$C NMR spectrum of **4d**.

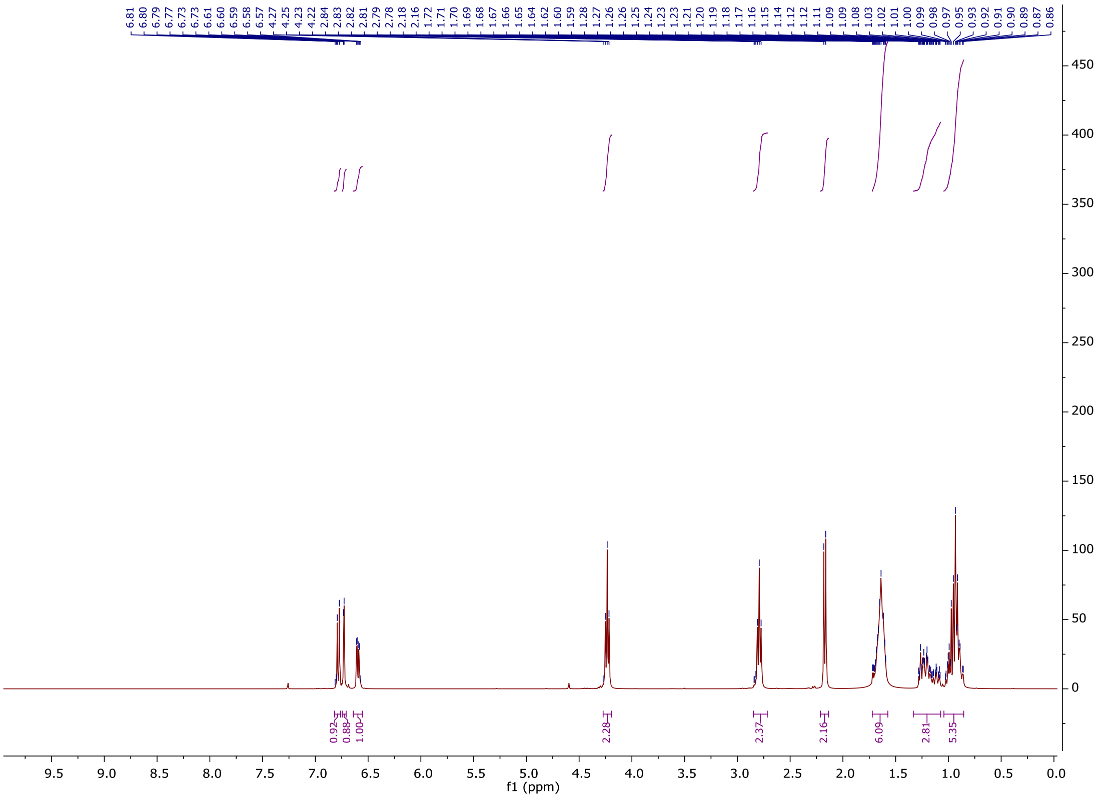

$^{1}$H NMR spectrum of **4e**

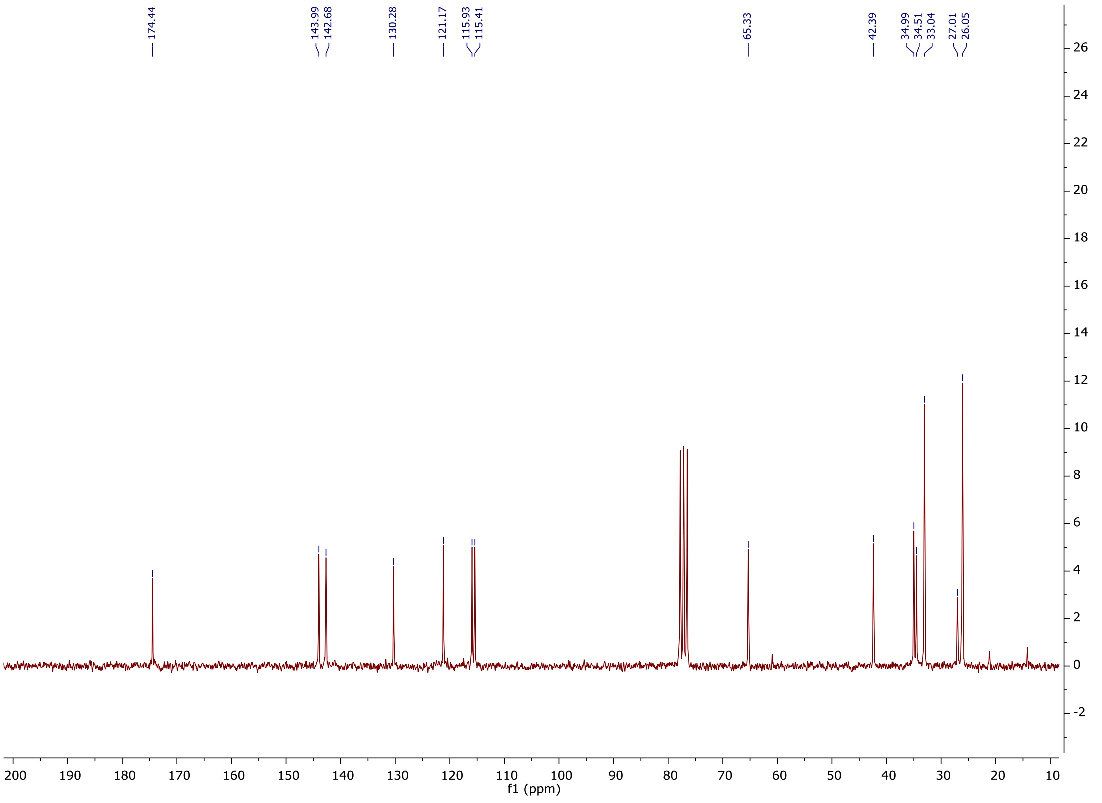

$^{13}$C NMR spectrum of **4e**

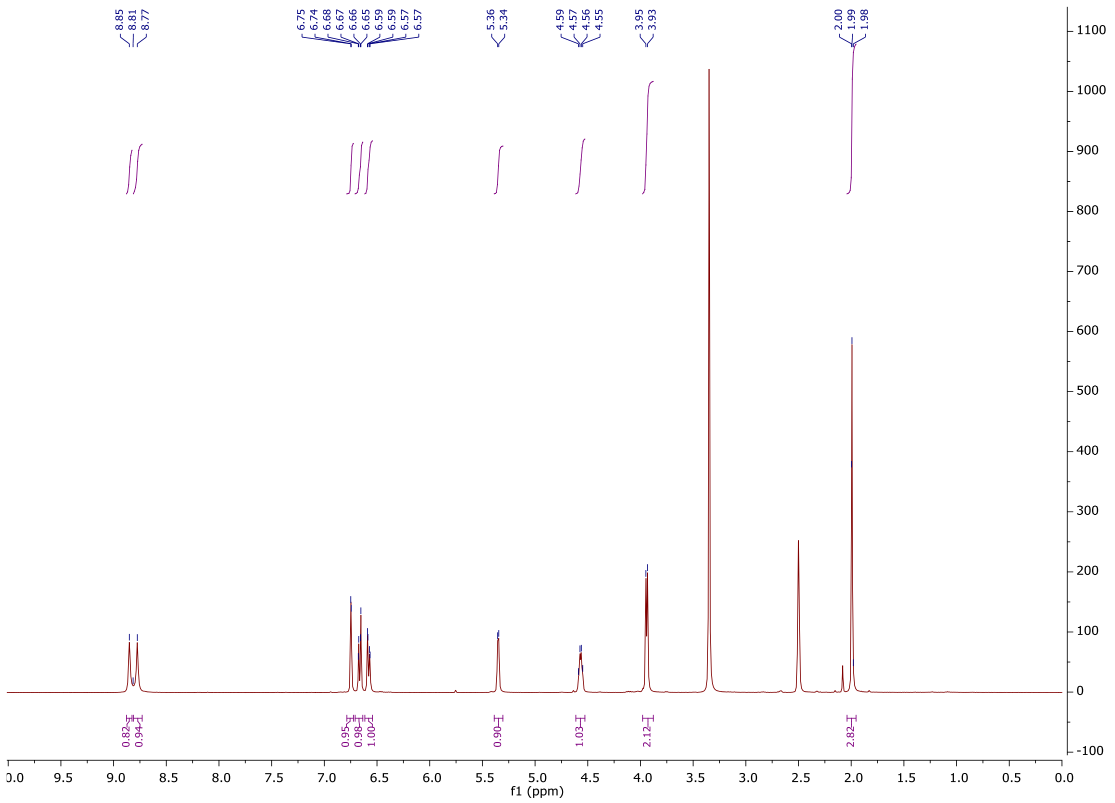


$^1$H NMR spectrum of **5a**.

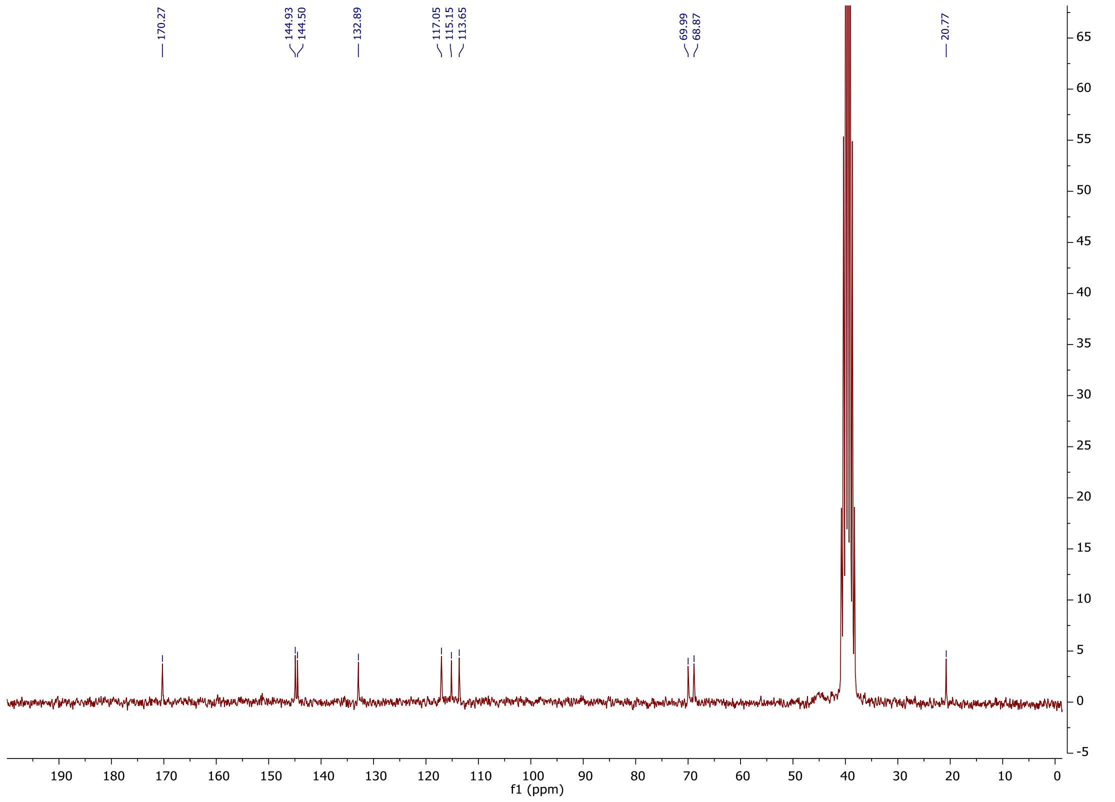


$^{13}$C NMR spectrum of **5a**

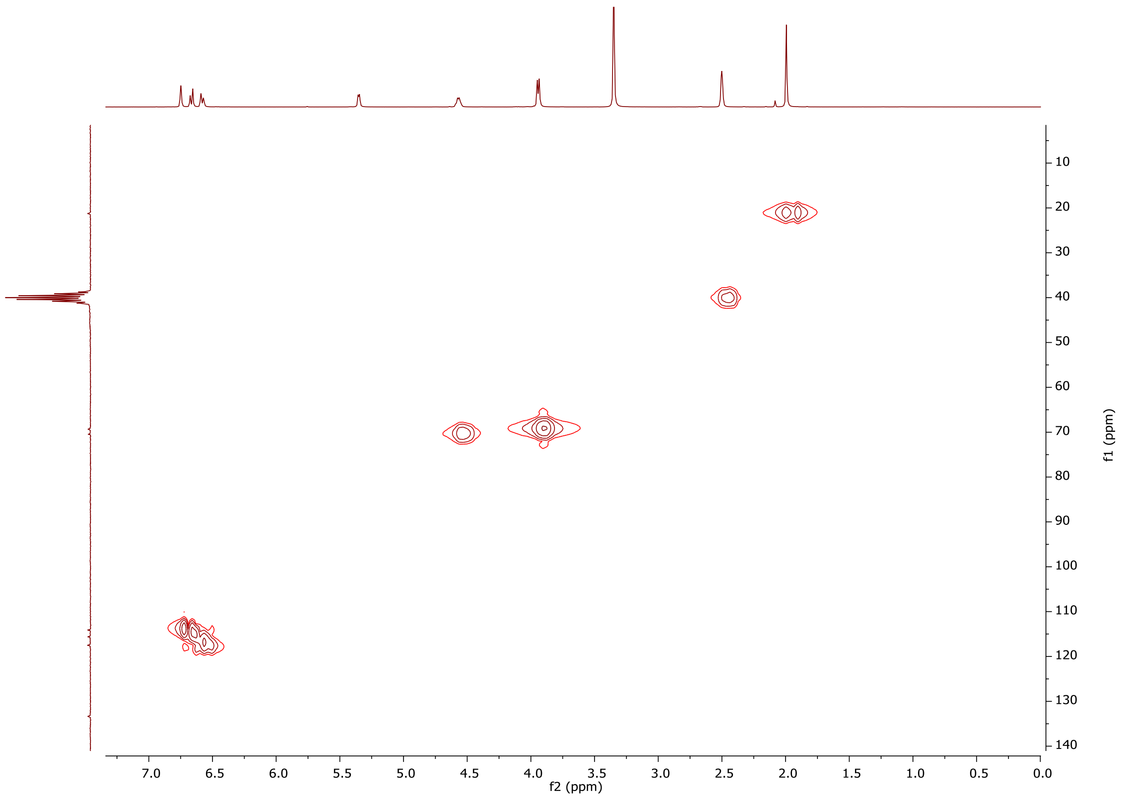


HSQC spectrum of **5a**

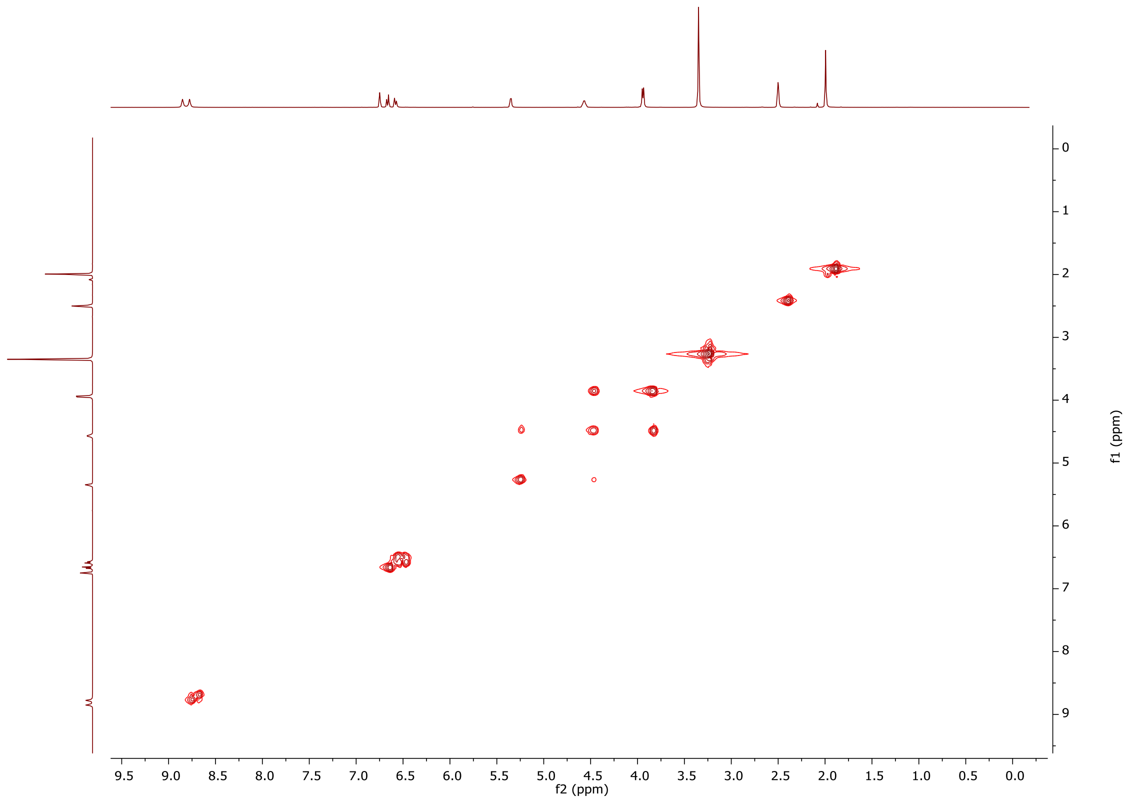


COSY spectrum of **5a**

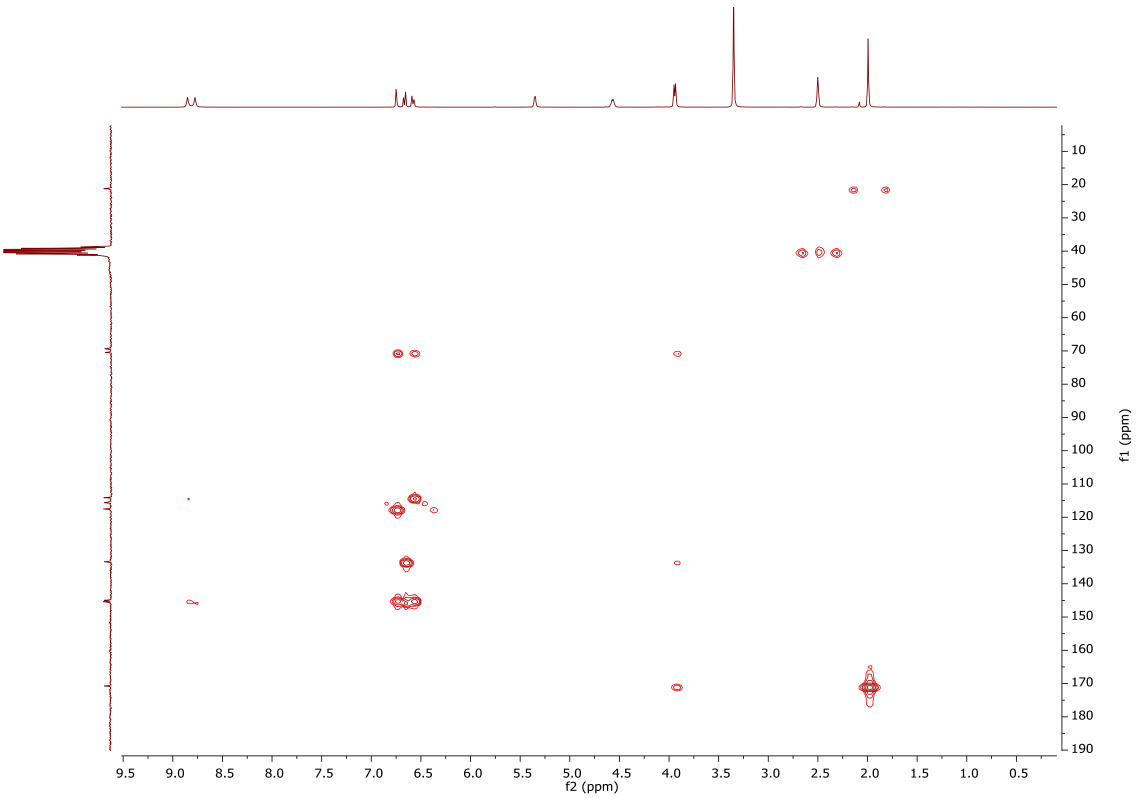


HMBC spectrum of **5a**

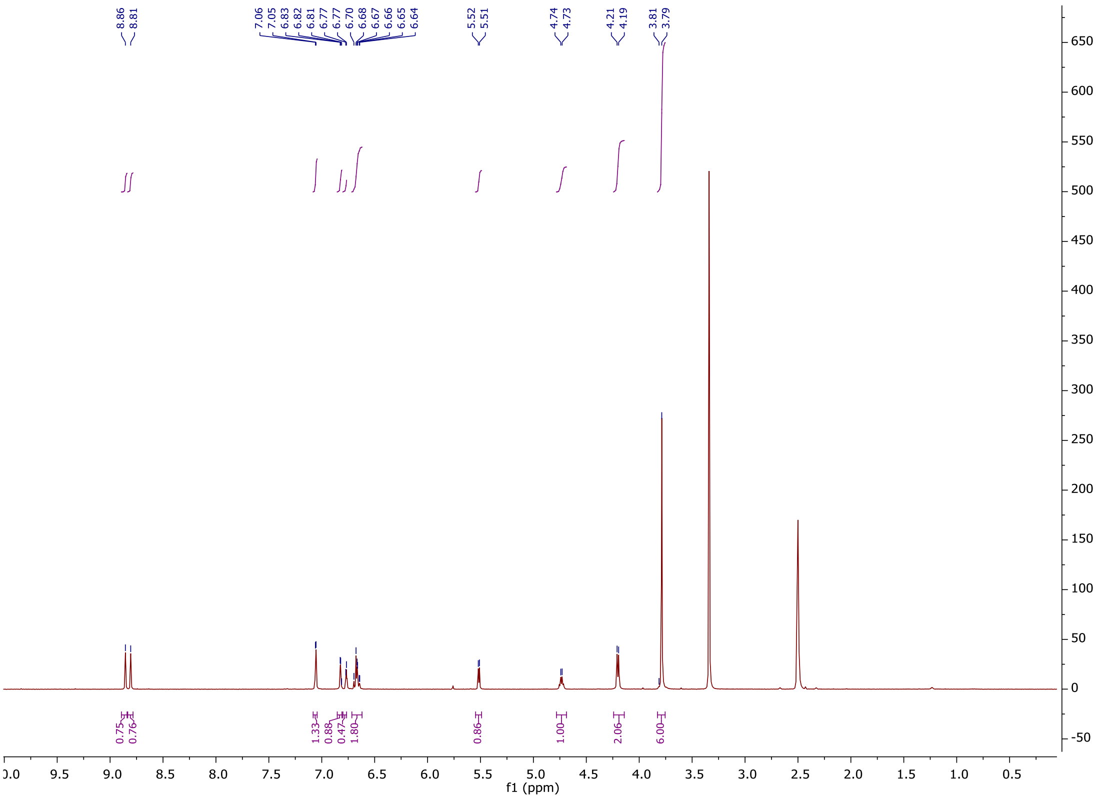


$^{1}$H NMR spectrum of **5b**.

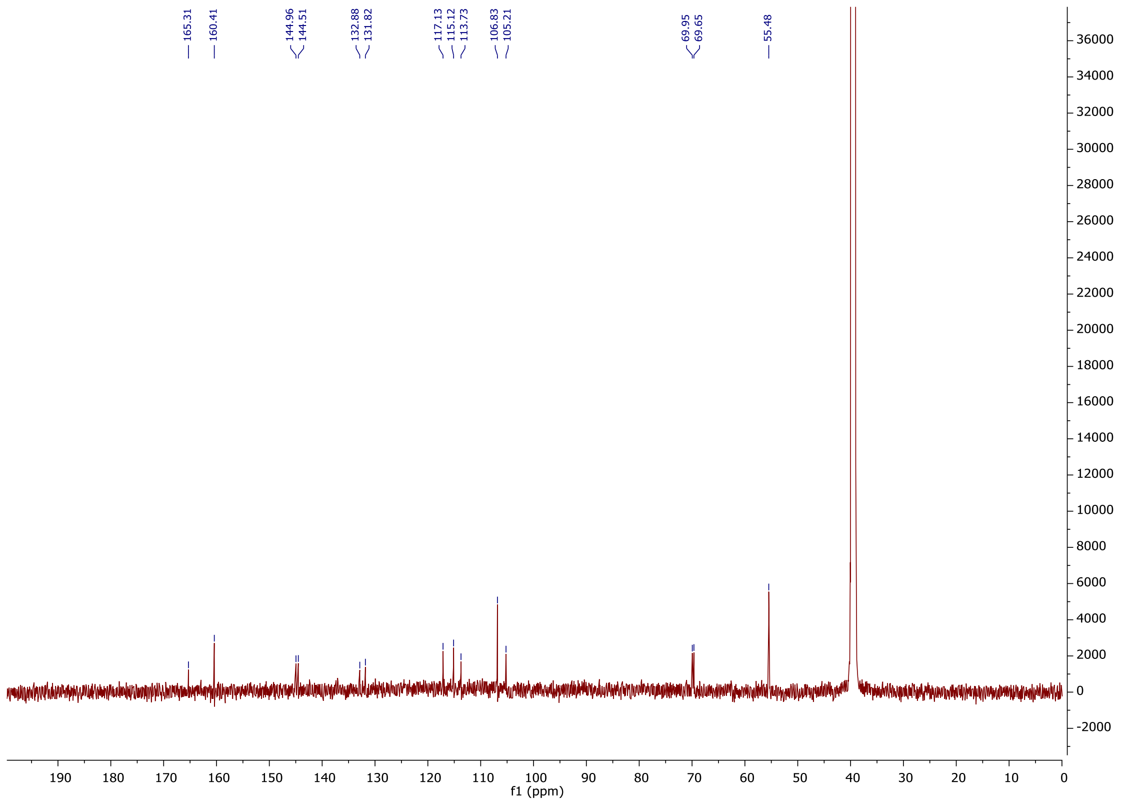


$^{13}$C NMR spectrum of **5b.**

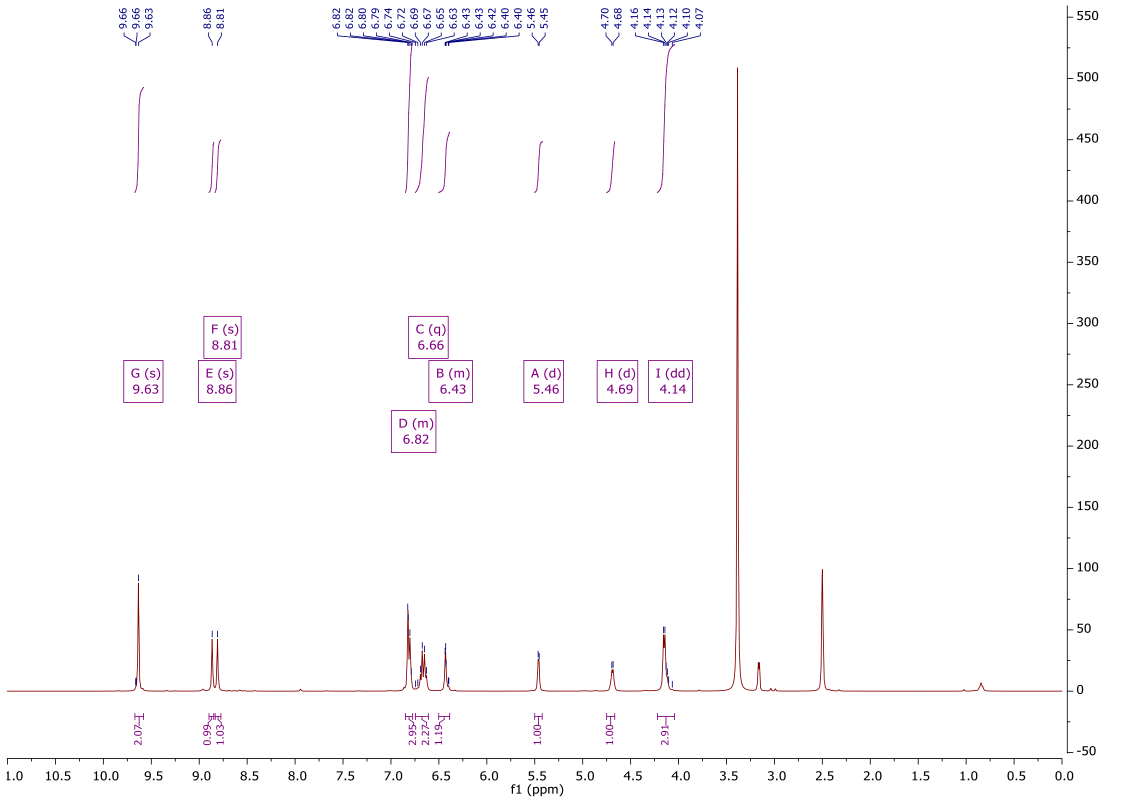


$^{1}$H NMR spectrum of **5c**.

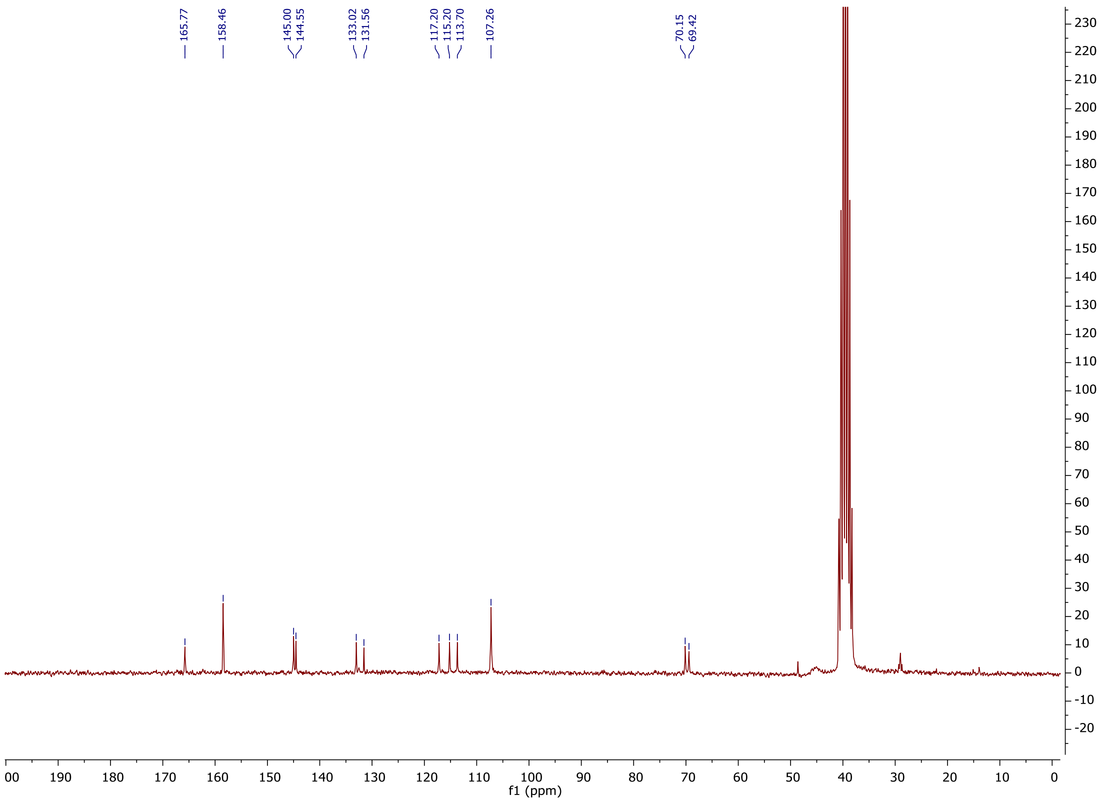


$^{13}$C NMR spectrum of **5c**.

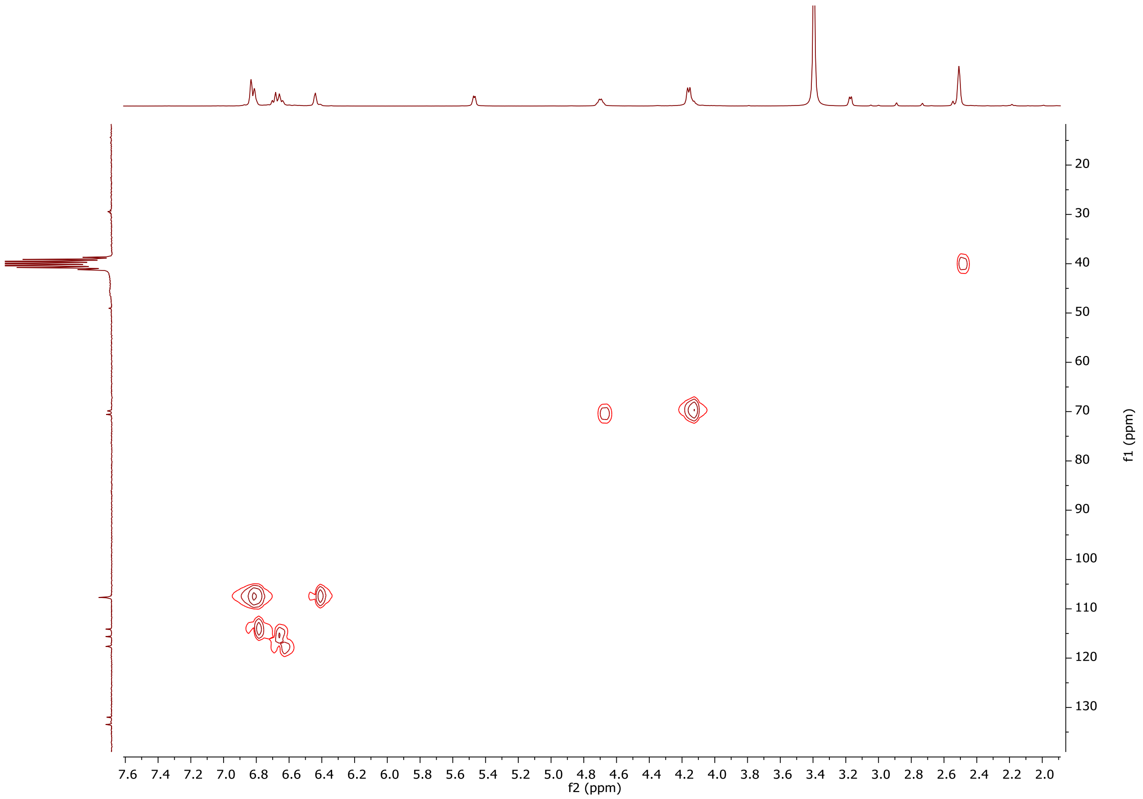


HSQC spectrum of **5c**

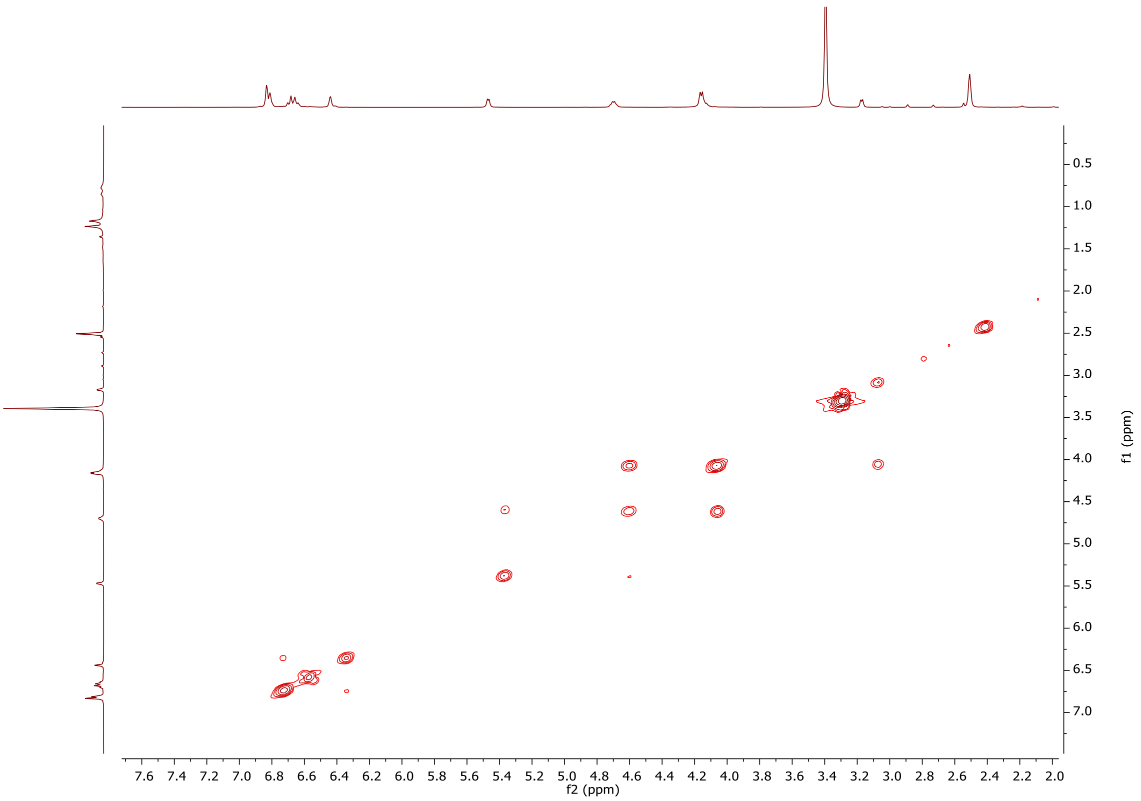


COSY spectrum of **5c**

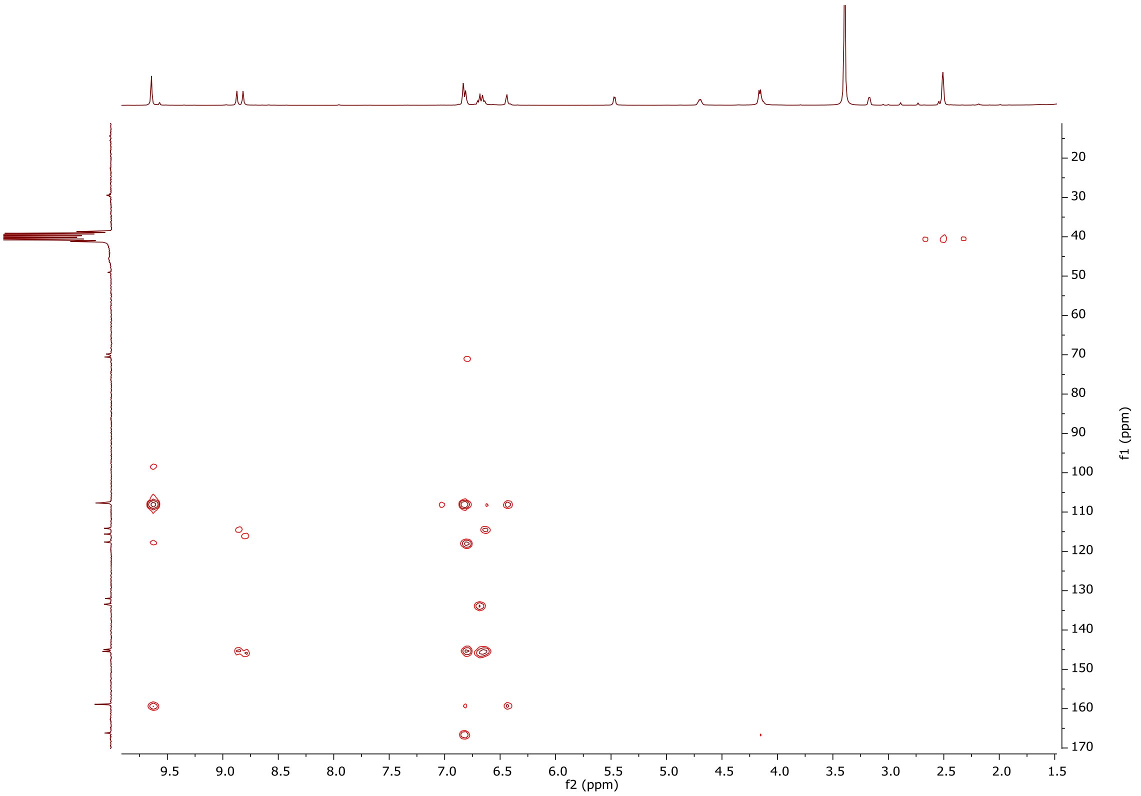


HMBC spectrum of **5c**

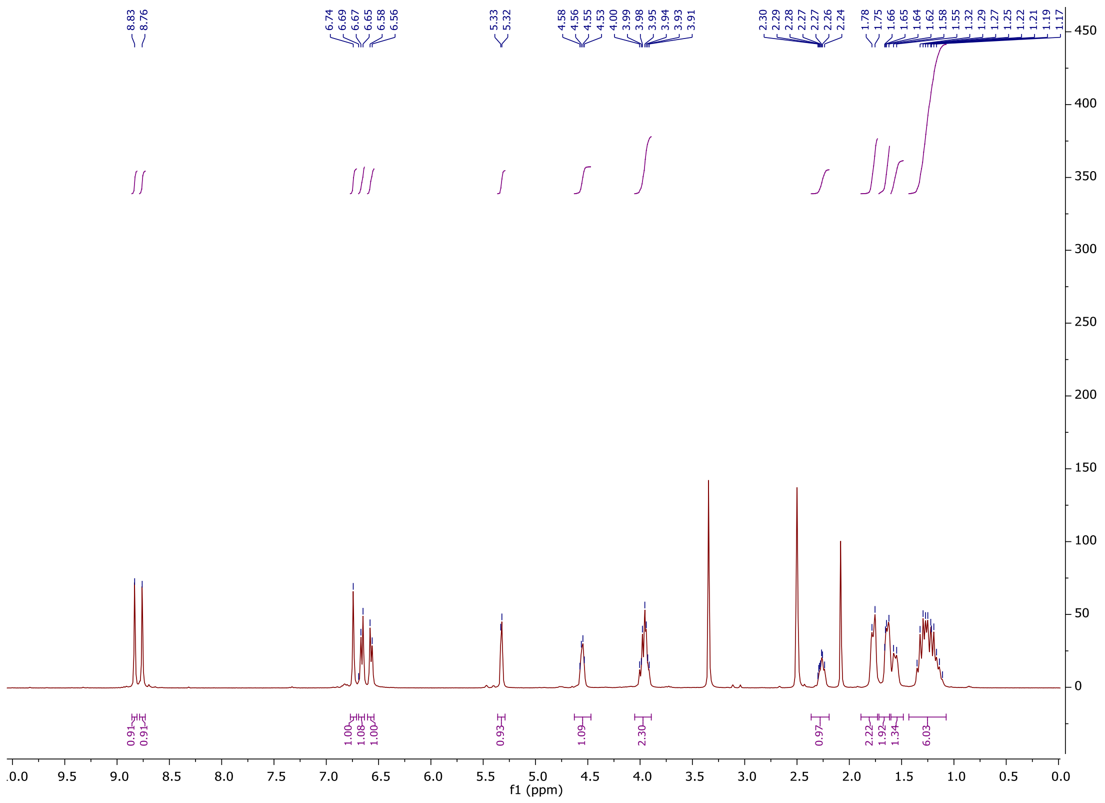


$^{1}$H NMR spectrum of **5d**.

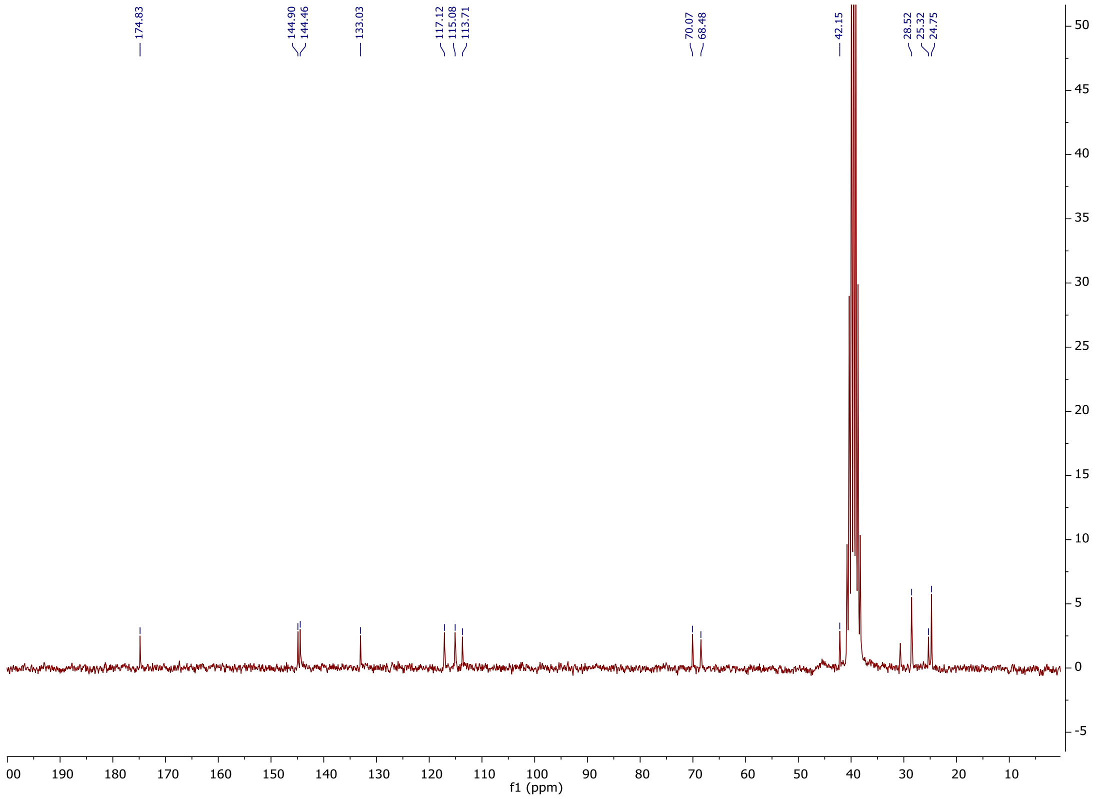


$^{13}$C NMR spectrum of **5d**.

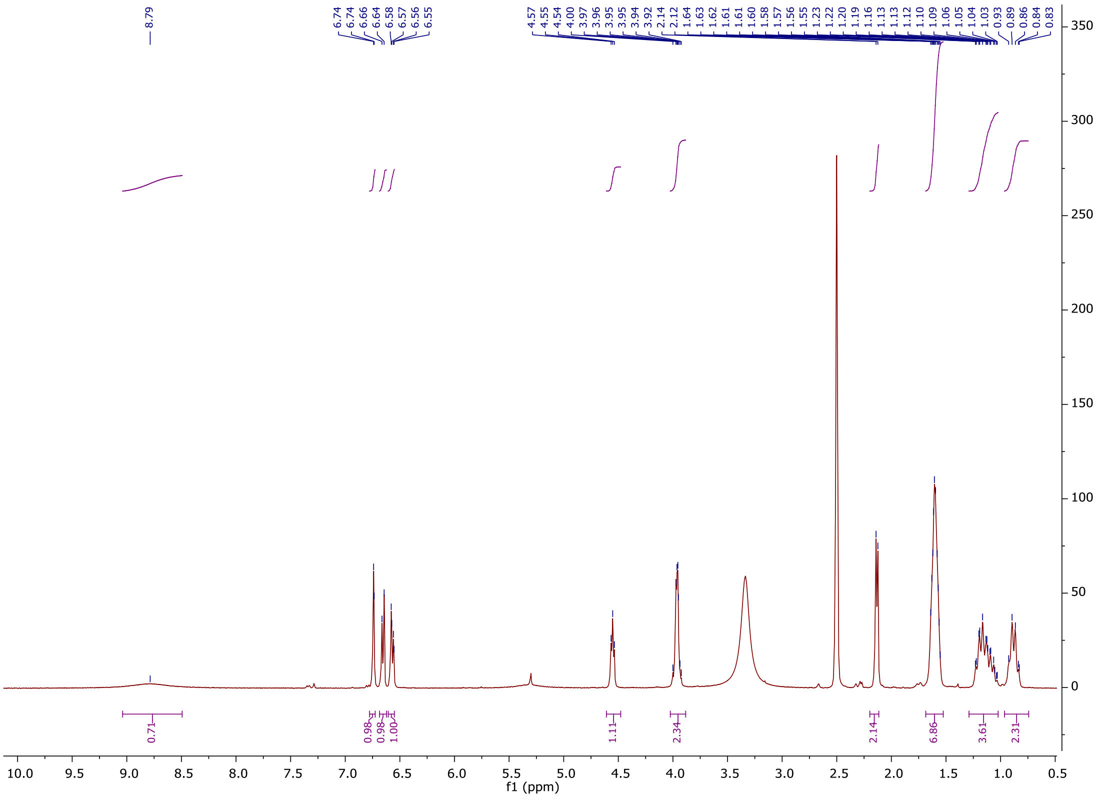

$^{1}$H NMR spectrum of **5e**.

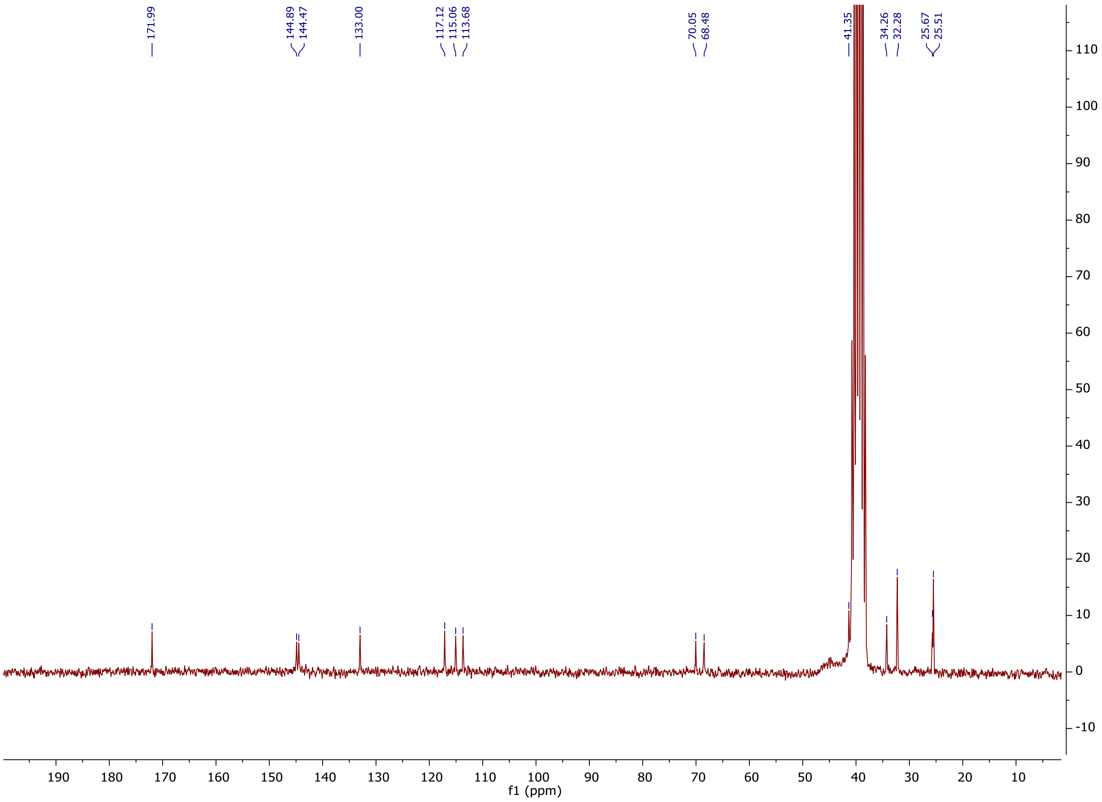

$^{13}$C NMR spectrum of **5e**

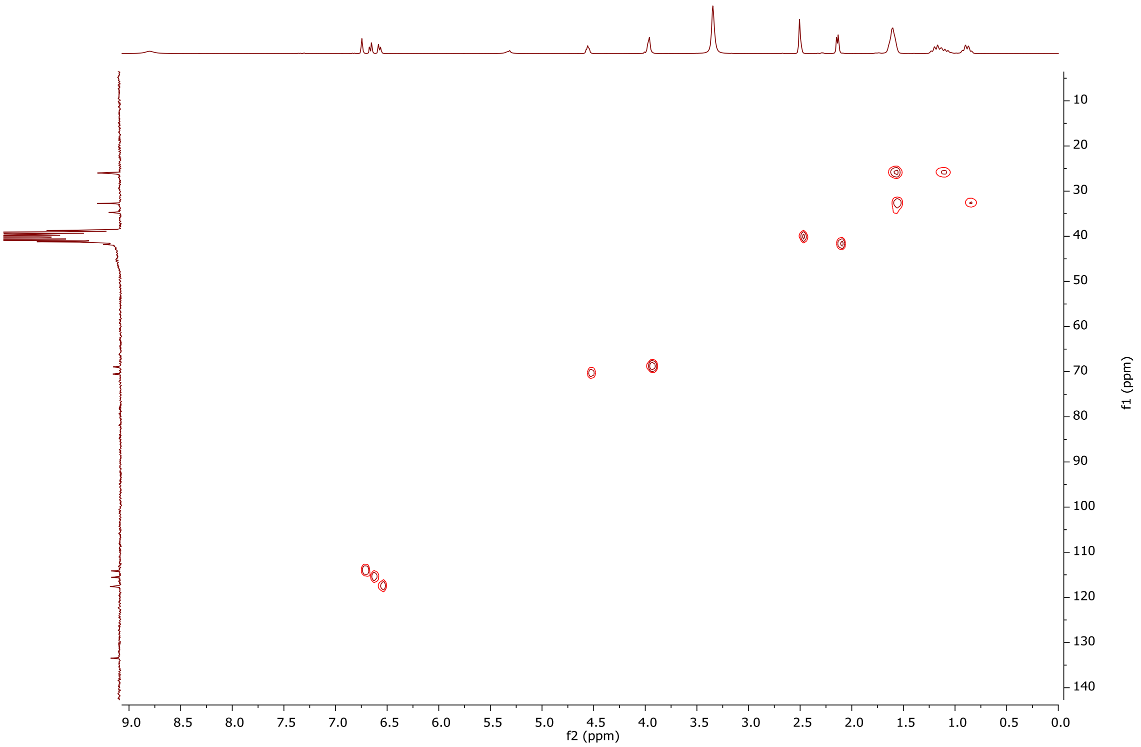


HSQC spectrum of **5e**

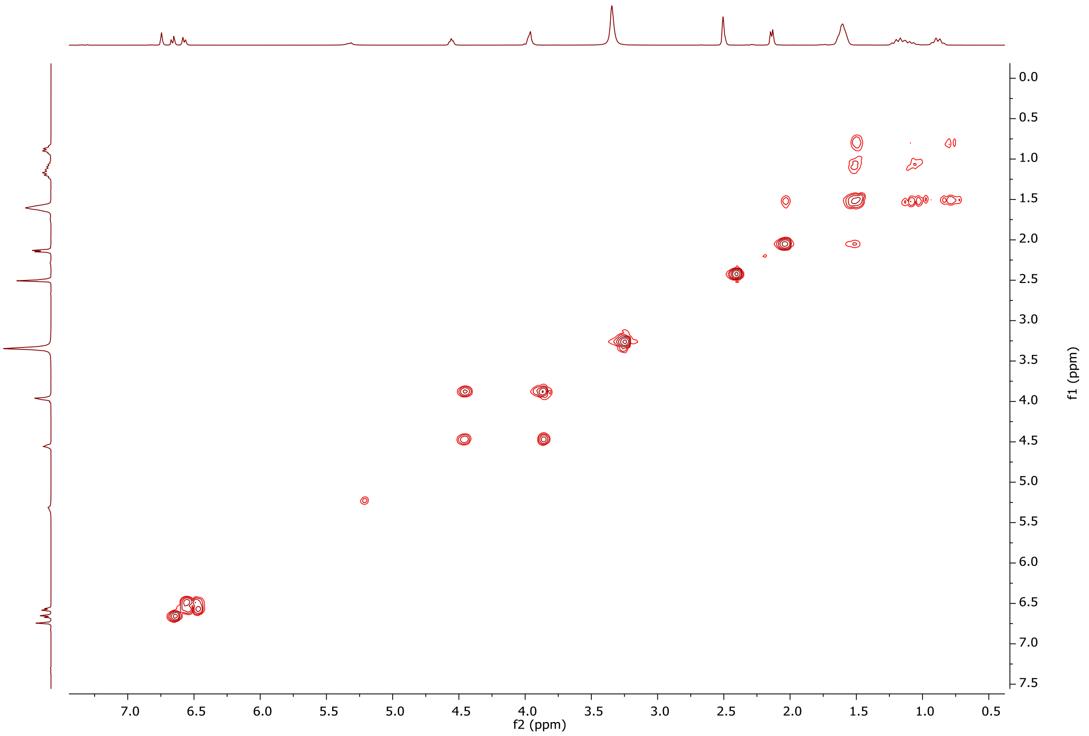


COSY spectrum of **5e**

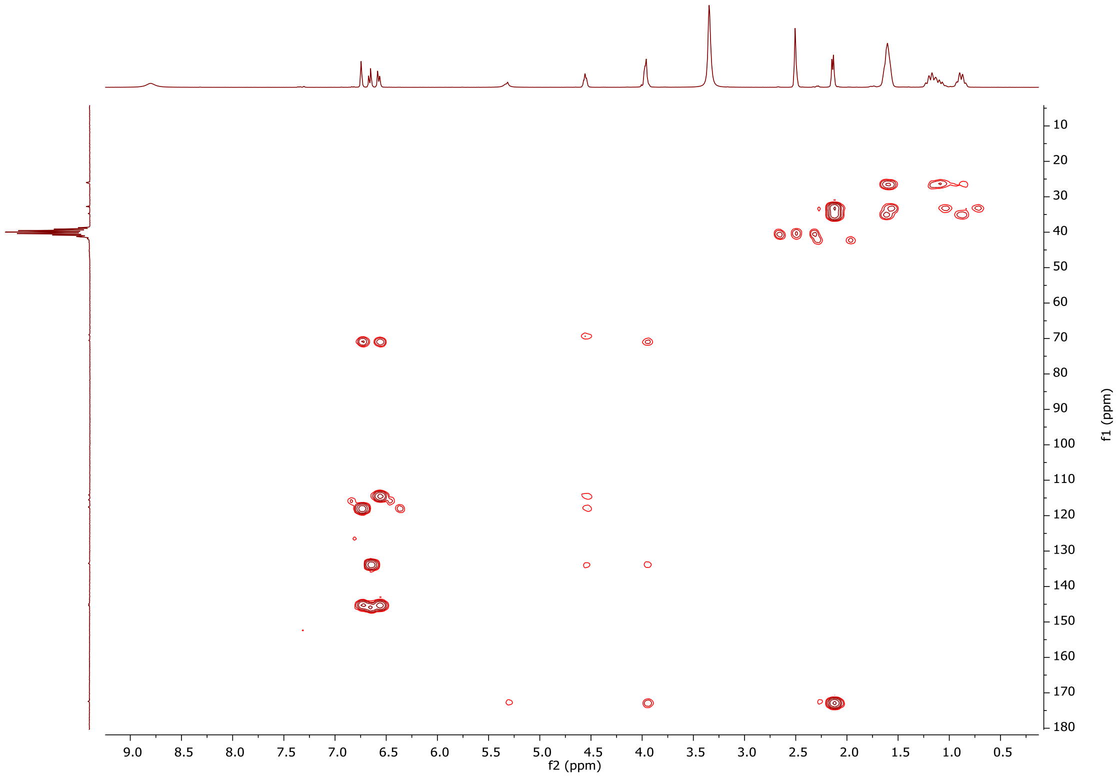

HMBC spectrum of **5e**

.

# Figures

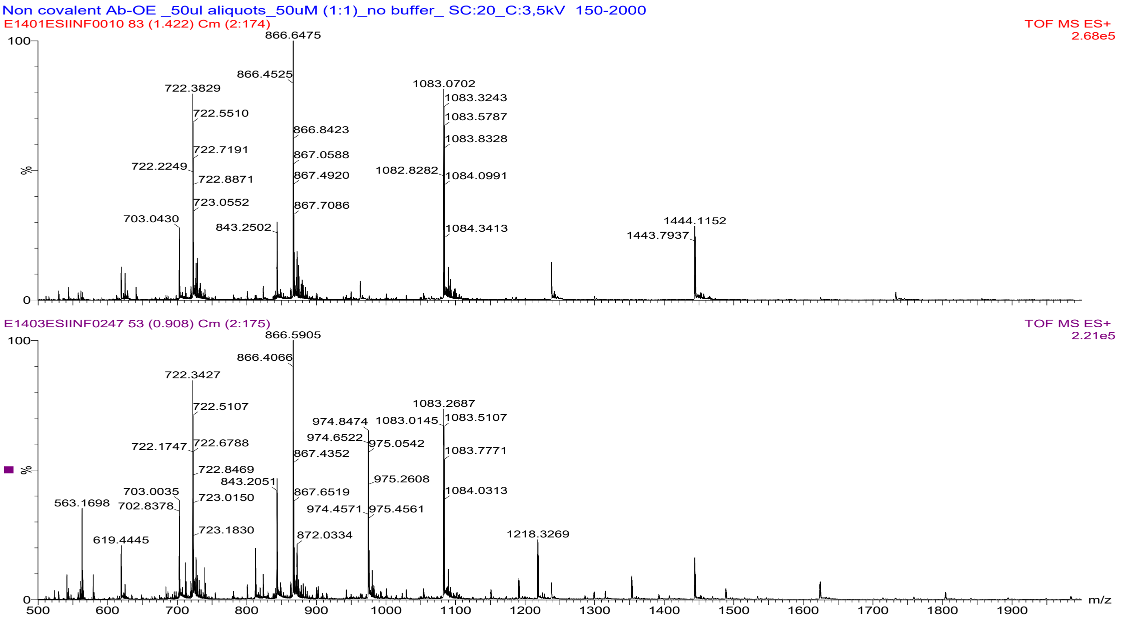


Figure S1: Positive Ionisation ESI Mass Spectrum of A$\beta$(1-40) along with the complex A$\beta$(1-40) and Oleuropein

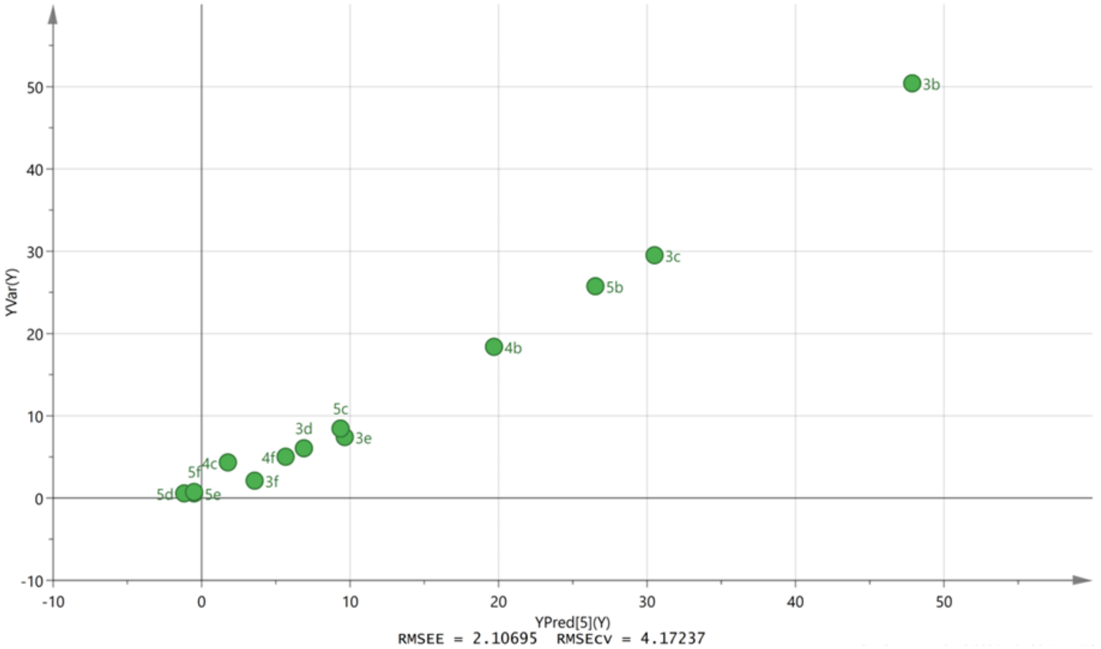


Figure S2: experimental vs calculated values (from the QSAR model) of the analogues complexing activity, based on the reduced model.

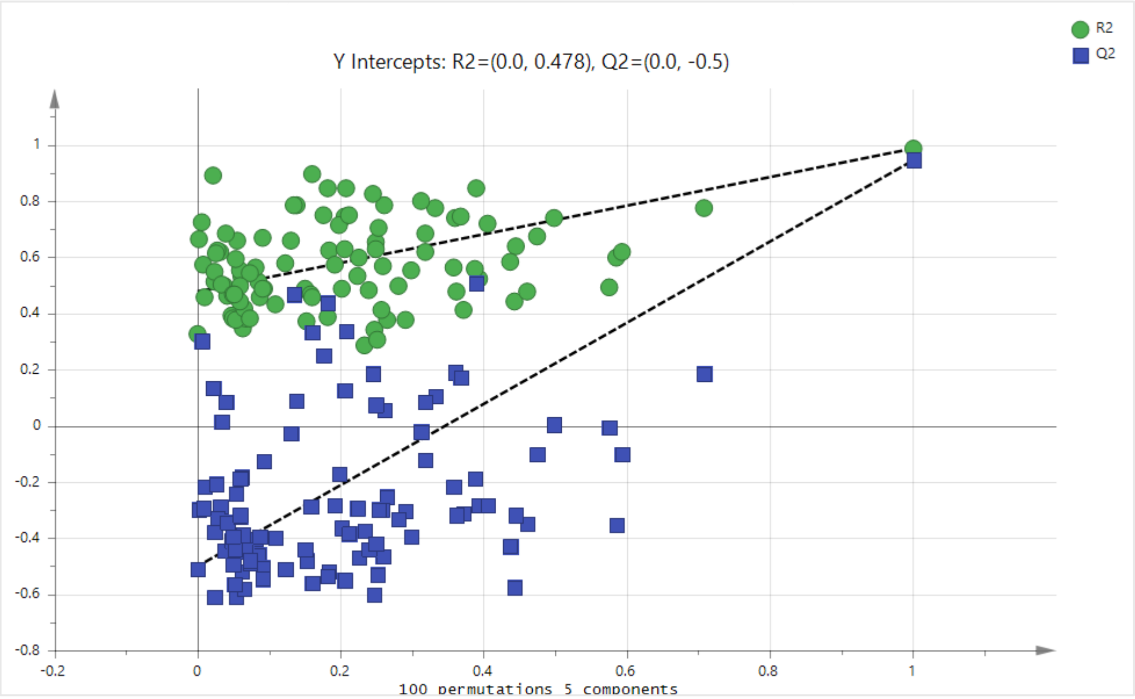


Figure S3: Permutation testing on the generated QSAR reduced model (n=100 permutations)

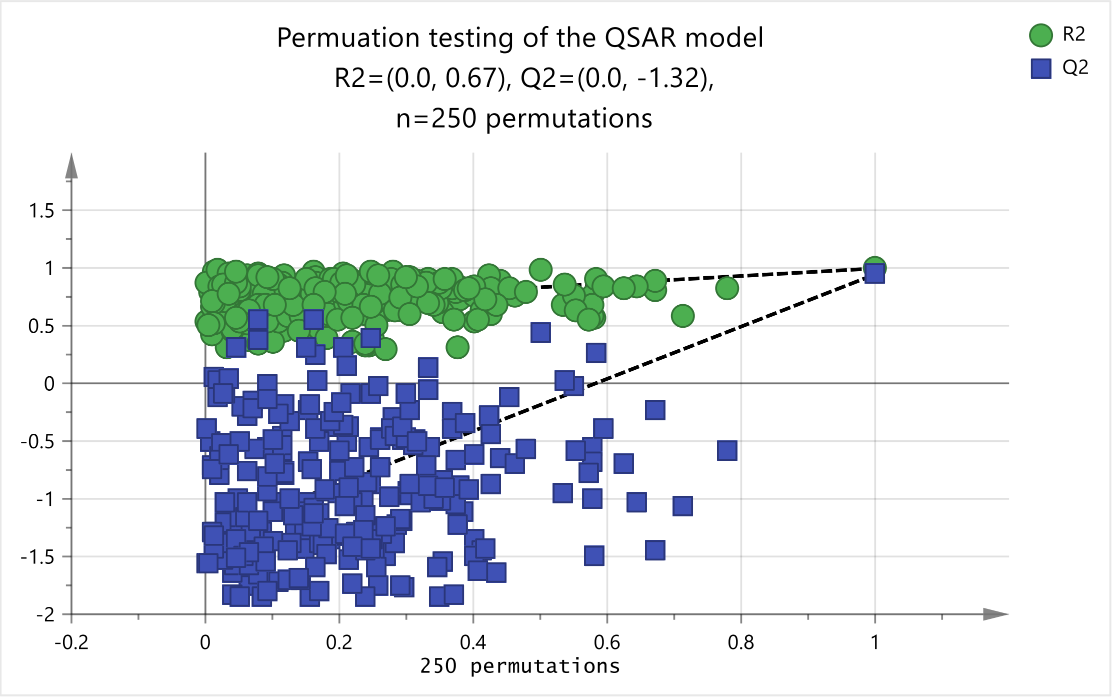


Figure S4: Permutation testing on the generated QSAR reduced model (n=250 permutations)

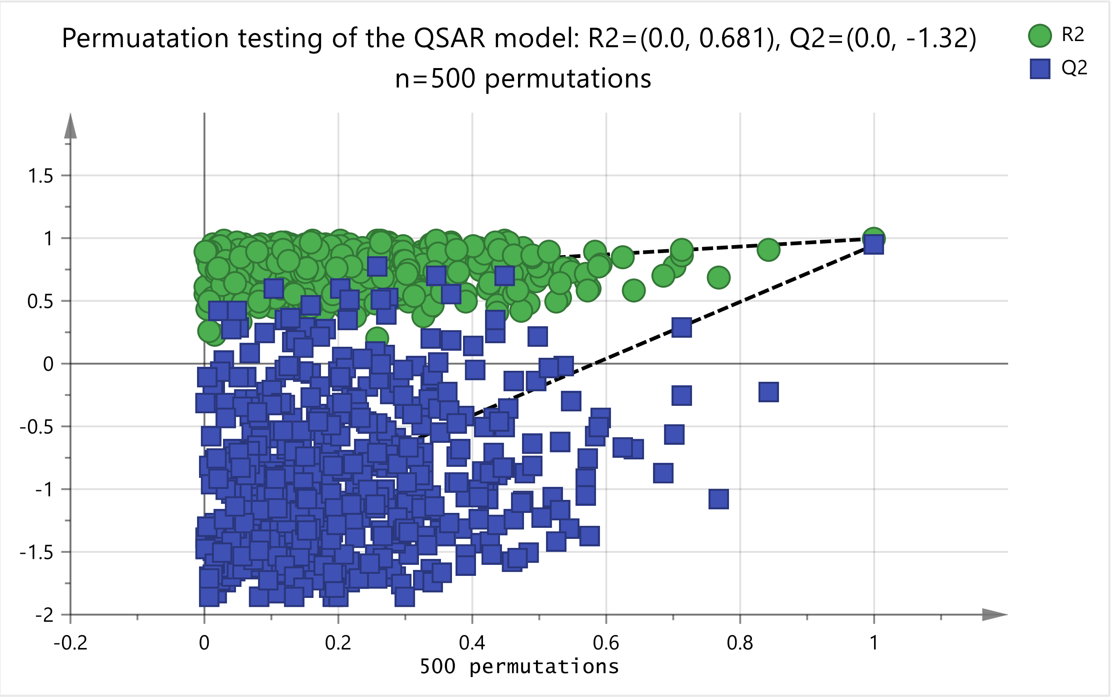


Figure S5: Permutation testing on the generated QSAR reduced model (n=500 permutations)

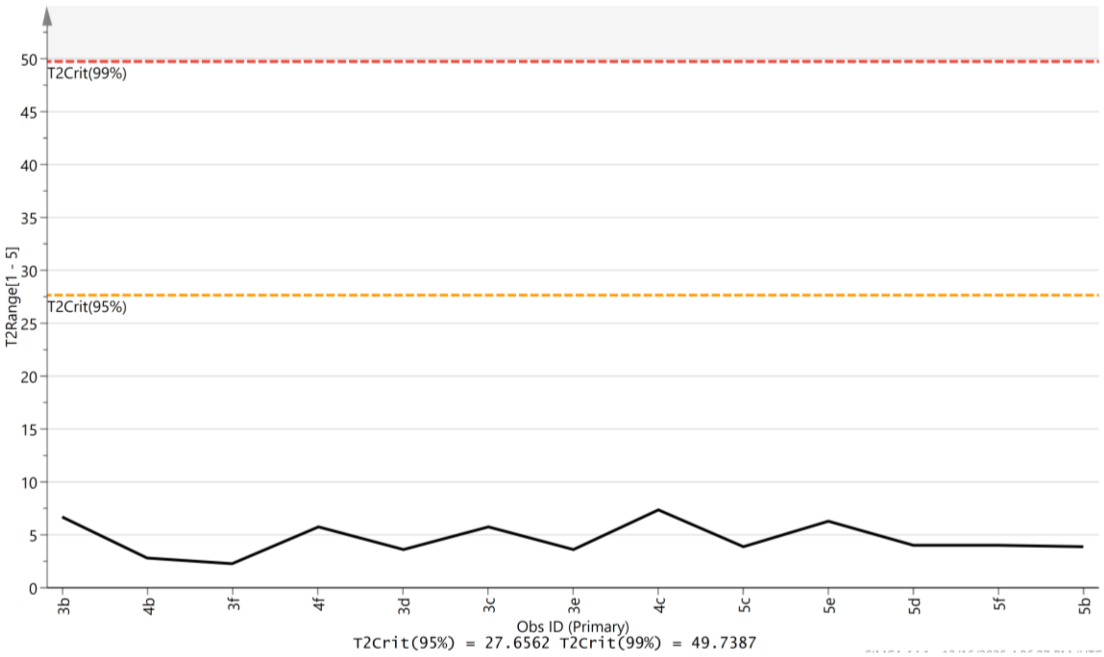


Figure S6: Validation of the QSAR model, T2 Hotteling testing for severe outliers of the reduced model

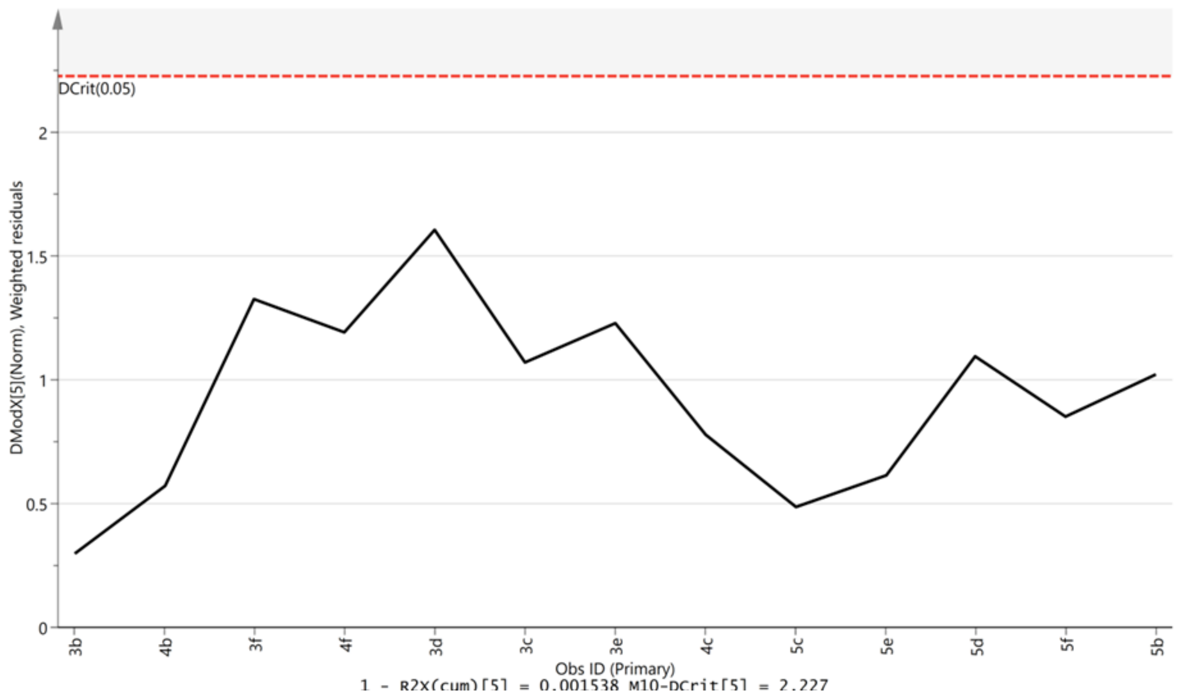


Figure S7: Validation of the QSAR reduced model DModX testing for mild outliers

# Tables

Table S1: Parameters used for the exploration of the non-covalent complexes between the A$\beta$ and the examined molecules

| | |
|---|---|
| Capillary (kV) | 3.5 |
| Source ( $^{O}$C) | 120 |
| L.M. | 4.7 |
| H.M. | 15.0 |
| Collision Energy (V) | 5.0 |
| Sampling Cone (V) | 2.0 |
| Desolvation ($^{O}$C) | 220 |
| Cell Entrance (V) | 2.0 |
| Extraction Cone (V) | 2.0 |
| Cone | Off |
| Ion Energy (V) | 0.5 |
| Cell Exit (V) | -10 |
| Ion Guide: (V) | 3.0 |
| Desolvation (L/hr) | 400 |
| Pre Filter (V) | 2.0 |
| Scan range (m/z) | 150-2000 |
| Detector (V) | 1900 |

Table S2: Cross validation testing

| **Number of groups (k)** | **$R^2$** | **$Q^2$** |
|---|---|---|
| 2 | 0.987 | 0.934 |
| 5 | 0.987 | 0.925 |
| 7 | 0.987 | 0.944 |
| 10 | 0.987 | 0.922 |
| 16 (Leave one out) | 0.996 | 0.935 |
